# Correction: Incidence and prevalence of inflammatory bowel disease in UK primary care: a population-based cohort study

**DOI:** 10.1136/bmjopen-2019-036584corr1

**Published:** 2020-08-27

Pasvol TJ, Horsfall L, Bloom S, *et al* Incidence and prevalence of inflammatory bowel disease in UK primary care: a population-based cohort study *BMJ Open* 2020;10:e036584. doi: 10.1136/bmjopen-2019-036584

This article was previously published with the wrong licence. The correct licence for the paper is CC-BY.

