# Supplementary material for: Incidence and prevalence of inflammatory bowel disease in UK primary care: a population-based cohort study
Source: BMJ Open. 2020 Jul 19;10(7):e036584. doi: 10.1136/bmjopen-2019-036584 (PMC7371214; doi:10.1136/bmjopen-2019-036584)
Supplement: Supplementary data [file bmjopen-2019-036584supp001.pdf]

APPENDIX:

Appendix table.1 Interaction coefficients (adjusted IRR) for age-time interactions*			
ageband (years)	Any IBD (95% CI)	CD (95% CI)	UC (95% CI)
0-9	1.019 (0.993-1.048)	1.032 (0.996-1.070)	1.017 (0.973-1.063)
10-16	1.027 (1.015-1.039)	1.025 (1.009-1.041)	1.024 (1.004-1.044)
17-40	1.003 (0.999-1.008)	0.992 (0.984-0.999)	1.005 (0.998-1.012)
40+	0.978 (0.974-0.982)	0.980 (0.972-0.987)	0.970 (0.965-0.976)
*Adjusted for other variables considered; Sex, ageband, year, region, Townsend quintile, respectively			

Appendix fig.1 Crude incidence estimates of IBD, stratified by calendar year, over the period 2000-2017 (sensitivity analysis)

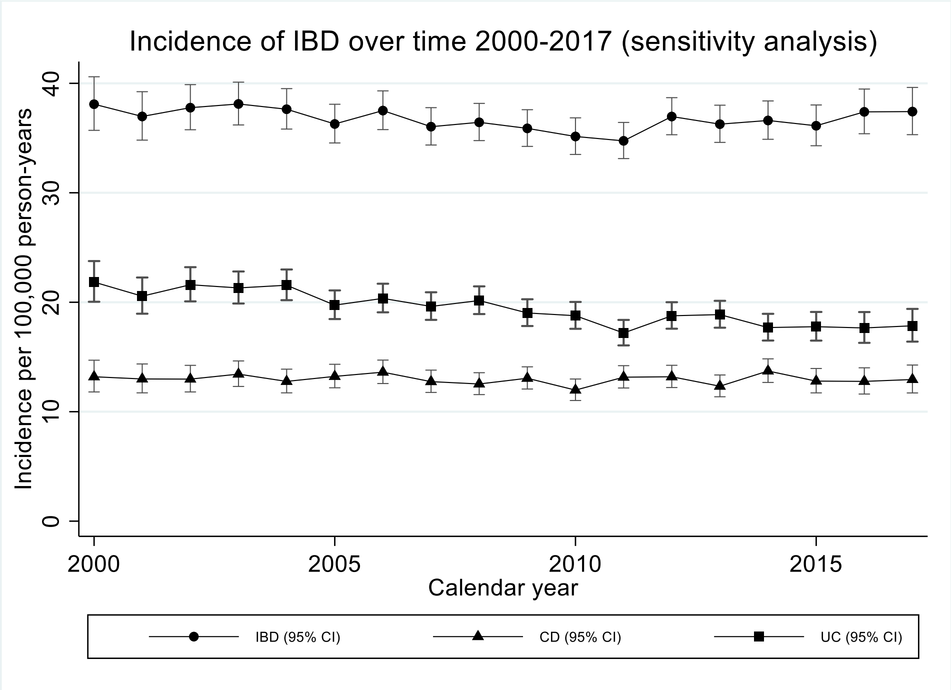

Appendix fig.2 Crude incidence estimates of IBD by five year age bands (primary analysis)

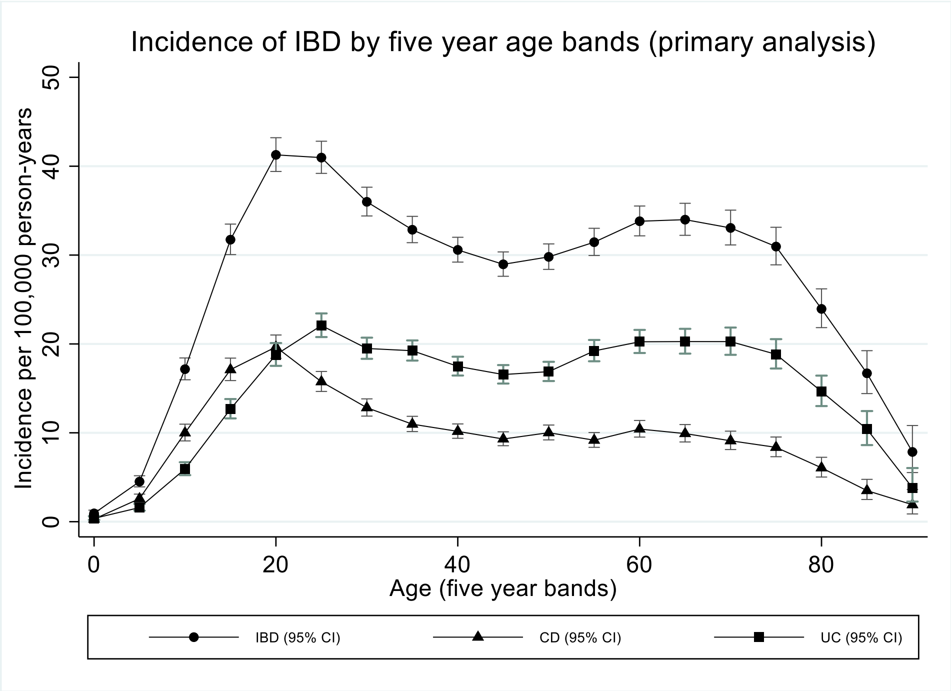

Appendix fig.3 Crude incidence estimates of IBD by five year age bands (sensitivity analysis)

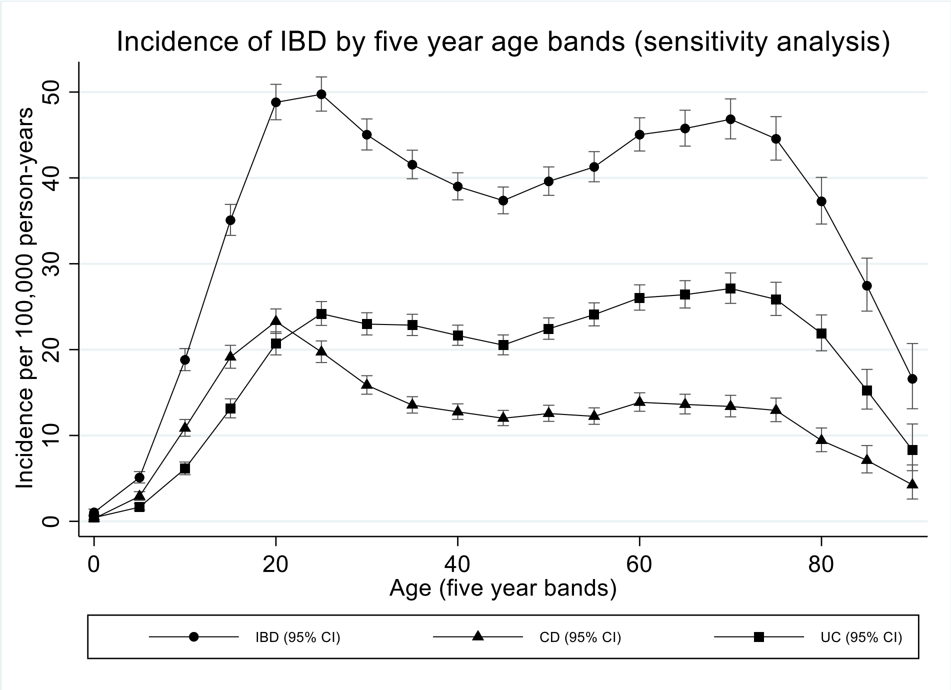

Read code lists:

Any IBD

**medcode**	**description**
14C4.11	H/O: ulcerative colitis
8Cc5.00	Management of inflammatory bowel disease
8Cc5.11	Management of IBD (inflammatory bowel disease)
J08z900	Orofacial Crohn's disease
J4...12	Inflammatory bowel disease
J40..00	Regional enteritis - Crohn's disease
J40..11	Crohn's disease
J400.00	Regional enteritis of the small bowel
J400000	Regional enteritis of the duodenum
J400100	Regional enteritis of the jejunum
J400200	Crohn's disease of the terminal ileum
J400300	Crohn's disease of the ileum unspecified
J400400	Crohn's disease of the ileum NOS
J400500	Exacerbation of Crohn's disease of small intestine

J400z00	Crohn's disease of the small bowel NOS
J401.00	Regional enteritis of the large bowel
J401000	Regional enteritis of the colon
J401100	Regional enteritis of the rectum
J401200	Exacerbation of Crohn's disease of large intestine
J401z00	Crohn's disease of the large bowel NOS
J401z11	Crohn's colitis
J402.00	Regional ileocolitis
J40z.00	Regional enteritis NOS
J40z.11	Crohn's disease NOS
J41..12	Ulcerative colitis and/or proctitis
J410.00	Ulcerative proctocolitis
J410100	Ulcerative colitis
J410200	Ulcerative rectosigmoiditis
J410300	Ulcerative proctitis
J410400	Exacerbation of ulcerative colitis
J410z00	Ulcerative proctocolitis NOS
J413.00	Ulcerative pancolitis
J41y.00	Other idiopathic proctocolitis
J41yz00	Other idiopathic proctocolitis NOS
J41z.00	Idiopathic proctocolitis NOS
J4z6.00	Indeterminate colitis
Jyu4000	[X]Other Crohn's disease
Jyu4100	[X]Other ulcerative colitis

CD

**medcode**	**description**
J08z900	Orofacial Crohn's disease
J40..00	Regional enteritis - Crohn's disease
J40..11	Crohn's disease
J400.00	Regional enteritis of the small bowel

J400000	Regional enteritis of the duodenum
J400100	Regional enteritis of the jejunum
J400200	Crohn's disease of the terminal ileum
J400300	Crohn's disease of the ileum unspecified
J400400	Crohn's disease of the ileum NOS
J400500	Exacerbation of Crohn's disease of small intestine
J400z00	Crohn's disease of the small bowel NOS
J401.00	Regional enteritis of the large bowel
J401000	Regional enteritis of the colon
J401100	Regional enteritis of the rectum
J401200	Exacerbation of Crohn's disease of large intestine
J401z00	Crohn's disease of the large bowel NOS
J401z11	Crohn's colitis
J402.00	Regional ileocolitis
J40z.00	Regional enteritis NOS
J40z.11	Crohn's disease NOS
Jyu4000	[X]Other Crohn's disease

UC

**medcode**	**description**
14C4.11	H/O: ulcerative colitis
J41..12	Ulcerative colitis and/or proctitis
J410.00	Ulcerative proctocolitis
J410100	Ulcerative colitis
J410200	Ulcerative rectosigmoiditis
J410300	Ulcerative proctitis
J410400	Exacerbation of ulcerative colitis
J410z00	Ulcerative proctocolitis NOS
J413.00	Ulcerative pancolitis
J41y.00	Other idiopathic proctocolitis
J41yz00	Other idiopathic proctocolitis NOS

J41z.00	Idiopathic proctocolitis NOS
Jyu4100	[X]Other ulcerative colitis

Drugs related to IBD

**drugcode**	**genericname**
30104978	Adalimumab 40mg/0.4ml solution for injection pre-filled syringes
30105978	Adalimumab 40mg/0.4ml solution for injection pre-filled syringes
30106978	Adalimumab 40mg/0.4ml solution for injection pre-filled disposable devices
53189979	Ciclosporin 50mg capsules
53190979	Ciclosporin 100mg capsules
53191979	Ciclosporin 25mg capsules
53913979	Mesalazine 800mg gastro-resistant tablets
54552979	Mesalazine 400mg gastro-resistant tablets
55164978	Mesalazine 4g modified-release granules sachets sugar free
55165978	Mesalazine 4g modified-release granules sachets sugar free
55193978	Mercaptopurine 75mg tablets
55575979	Ciclosporin 50mg capsules
55576979	Ciclosporin 100mg capsules
55577979	Ciclosporin 25mg capsules
55799978	Mercaptopurine 30mg capsules
56901978	Ciclosporin 100mg capsules
56902978	Ciclosporin 25mg capsules
58121979	Mercaptopurine 25mg tablets
58800979	Mesalazine 1.2g gastro-resistant modified-release tablets
60097979	Prednisolone 20mg/application foam enema
60124979	Ciclosporin 50mg capsules
60584979	Mesalazine 3g gastro-resistant modified-release granules sachets sugar free
60585979	Mesalazine 3g gastro-resistant modified-release granules sachets sugar free
61544979	Adalimumab 40mg/0.8ml solution for injection vials
61545979	Adalimumab 40mg/0.8ml solution for injection vials
62953979	Sulfasalazine 250mg/5ml oral suspension sugar free

64115979	Methotrexate 25mg/3ml solution for injection pre-filled syringes
64868979	Mesalazine 2g modified-release granules sachets sugar free
65098979	Sulfasalazine 500mg/5ml oral suspension
67089979	Mesalazine 1.2g gastro-resistant modified-release tablets
69316979	Azathioprine 125mg/5ml oral suspension
70274978	Azathioprine 50mg tablets
70879979	Adalimumab 40mg prefilled pen
70880979	Adalimumab 40mg/0.8ml solution for injection pre-filled disposable devices
72688978	Ciclosporin 50mg capsules
73065978	Methotrexate 7.5mg/0.15ml solution for injection pre-filled disposable devices
73066978	Methotrexate 7.5mg/0.15ml solution for injection pre-filled disposable devices
73067978	Methotrexate 30mg/0.6ml solution for injection pre-filled disposable devices
73068978	Methotrexate 30mg/0.6ml solution for injection pre-filled disposable devices
73069978	Methotrexate 27.5mg/0.55ml solution for injection pre-filled disposable devices
73071978	Methotrexate 25mg/0.5ml solution for injection pre-filled disposable devices
73072978	Methotrexate 25mg/0.5ml solution for injection pre-filled disposable devices
73073978	Methotrexate 22.5mg/0.45ml solution for injection pre-filled disposable devices
73074978	Methotrexate 22.5mg/0.45ml solution for injection pre-filled disposable devices
73075978	Methotrexate 20mg/0.4ml solution for injection pre-filled disposable devices
73076978	Methotrexate 20mg/0.4ml solution for injection pre-filled disposable devices
73077978	Methotrexate 17.5mg/0.35ml solution for injection pre-filled disposable devices
73078978	Methotrexate 17.5mg/0.35ml solution for injection pre-filled disposable devices
73079978	Methotrexate 15mg/0.3ml solution for injection pre-filled disposable devices
73080978	Methotrexate 15mg/0.3ml solution for injection pre-filled disposable devices
73081978	Methotrexate 12.5mg/0.25ml solution for injection pre-filled disposable devices
73082978	Methotrexate 12.5mg/0.25ml solution for injection pre-filled disposable devices
73083978	Methotrexate 10mg/0.2ml solution for injection pre-filled disposable devices
73084978	Methotrexate 10mg/0.2ml solution for injection pre-filled disposable devices
76424978	Mesalazine 1g modified-release tablets
76878978	Methotrexate 2.5mg tablets
78442979	Methotrexate 50mg/2ml solution for injection vials

78447979	Methotrexate 25mg/1ml solution for injection pre-filled syringes
78449979	Methotrexate 20mg/0.8ml solution for injection pre-filled syringes
78452979	Methotrexate 12.5mg/0.5ml solution for injection pre-filled syringes
79522979	Mercaptopurine 25mg/5ml oral suspension
79524979	Mercaptopurine 20mg/ml oral suspension
79739978	Sulfasalazine 500mg gastro-resistant tablets
79867978	Mesalazine 400mg gastro-resistant tablets
80925979	Azathioprine 25mg/5ml oral suspension
80927979	Azathioprine 25mg/5ml oral solution
80928998	Mesalazine 3g gastro-resistant modified-release granules sachets sugar free
80929979	Azathioprine 20mg/5ml oral suspension
80929998	Mesalazine 3g gastro-resistant modified-release granules sachets sugar free
80961979	Azathioprine 10mg/5ml oral suspension
81193998	Mesalazine 1g modified-release tablets
81194998	Mesalazine 1g modified-release tablets
81282998	Ciclosporin 100mg capsules
81283998	Ciclosporin 50mg capsules
81284998	Ciclosporin 25mg capsules
81490998	Methotrexate 27.5mg/0.55ml prefilled syringes
81491998	Methotrexate 22.5mg/0.45ml prefilled syringes
81492998	Methotrexate 17.5mg/0.35ml prefilled syringes
81493998	Methotrexate 12.5mg/0.25ml prefilled syringes
81494998	Methotrexate 27.5mg/0.55ml solution for injection pre-filled syringes
81495998	Methotrexate 22.5mg/0.45ml solution for injection pre-filled syringes
81496998	Methotrexate 17.5mg/0.35ml solution for injection pre-filled syringes
81498998	Methotrexate 12.5mg/0.25ml solution for injection pre-filled syringes
81638998	Methotrexate 30mg/1.5ml solution for injection pre-filled syringes
81640998	Methotrexate 25mg/1.25ml solution for injection pre-filled syringes
81642998	Methotrexate 20mg/1ml solution for injection pre-filled syringes
81643998	Methotrexate 7.5mg/0.75ml prefilled syringes
81683998	Mesalazine 1g suppositories

81689998	Mesalazine 500mg gastro-resistant tablets
81690998	Mesalazine 500mg gastro-resistant tablets
81771998	Ciclosporin 25mg capsules
81772998	Mesalazine 800mg gastro-resistant tablets
81773998	Ciclosporin 100mg capsules
81774998	Ciclosporin 50mg capsules
81815998	Methotrexate 30mg/0.6ml prefilled syringes
81816998	Methotrexate 30mg/0.6ml solution for injection pre-filled syringes
81868998	Mesalazine 400mg gastro-resistant tablets
82203998	Azathioprine 50mg tablets
82204998	Azathioprine 25mg tablets
82480998	Ciclosporin 100mg capsules
82481998	Ciclosporin 50mg capsules
82482998	Ciclosporin 25mg capsules
82840998	Methotrexate 25mg/0.5ml prefilled syringes
82841998	Methotrexate 20mg/0.4ml prefilled syringes
82842998	Methotrexate 15mg/0.3ml prefilled syringes
82843998	Methotrexate 10mg/0.2ml prefilled syringes
82844998	Methotrexate 7.5mg/0.15ml prefilled syringes
82845998	Methotrexate 25mg/0.5ml solution for injection pre-filled syringes
82846998	Methotrexate 20mg/0.4ml solution for injection pre-filled syringes
82847998	Methotrexate 15mg/0.3ml solution for injection pre-filled syringes
82848998	Methotrexate 10mg/0.2ml solution for injection pre-filled syringes
82849998	Methotrexate 7.5mg/0.15ml solution for injection pre-filled syringes
83228978	Ciclosporin 50mg capsules
83229978	Ciclosporin 100mg capsules
83485998	Azathioprine 10mg capsules
83503998	Mesalazine 1.5g gastro-resistant modified-release granules sachets sugar free
83504998	Mesalazine 1.5g gastro-resistant modified-release granules sachets sugar free
83559978	Mercaptopurine 50mg tablets
83743998	Mesalazine 2g modified-release granules sachets sugar free

83769998	Sulfasalazine 250mg/5ml oral suspension sugar free
83987998	Mesalazine 2g modified-release granules sachets sugar free
84059998	Mesalazine 1g modified-release granules sachets sugar free
84209998	Mesalazine 800mg gastro-resistant tablets
84290998	Mesalazine 1.2g gastro-resistant modified-release tablets
84291998	Mesalazine 1.2g gastro-resistant modified-release tablets
84438998	Methotrexate 2mg/ml oral solution sugar free
84439998	Methotrexate 10mg/5ml oral suspension
84636998	Budesonide 2mg foam enema
84637998	Budesonide 2mg foam enema
84741998	Prednisolone 40mg/100ml enema
84920998	Ciclosporin 250mg/5ml solution for infusion ampoules
84921998	Ciclosporin 50mg/1ml solution for infusion ampoules
84927998	Ciclosporin 250mg/5ml solution for infusion ampoules
84928998	Ciclosporin 50mg/1ml solution for infusion ampoules
85097998	Methotrexate 1g/40ml solution for injection vials
85100998	Methotrexate 7.5mg/5ml oral suspension
85188978	Methotrexate 2.5mg tablets
85252998	Azathioprine 50mg/5ml oral suspension
85556998	Mercaptopurine oral solution
85560998	Mesalazine 800mg gastro-resistant tablets
85639998	Methotrexate 7.5mg/0.75ml prefilled syringes
85640998	Methotrexate 10mg/ml prefilled syringes
85641998	Methotrexate 15mg/1.5ml prefilled syringes
85642998	Methotrexate 20mg/2ml prefilled syringes
85643998	Methotrexate 25mg/2.5ml prefilled syringes
85644998	Methotrexate 7.5mg/0.75ml solution for injection pre-filled syringes
85645998	Methotrexate 10mg/1ml solution for injection pre-filled syringes
85646998	Methotrexate 15mg/1.5ml solution for injection pre-filled syringes
85648998	Methotrexate 20mg/2ml solution for injection pre-filled syringes
85650998	Methotrexate 25mg/2.5ml solution for injection pre-filled syringes

85737998	Methotrexate 5g/50ml solution for infusion vials
85738998	Methotrexate 1g/10ml solution for injection vials
85776998	Methotrexate oral solution
85777998	Methotrexate 12.5mg/5ml oral suspension
86327998	Methotrexate 2.5mg/5ml oral suspension
86339998	Methotrexate 5mg/0.2ml solution for injection pre-filled syringes
86342998	Methotrexate 22.5mg/0.9ml solution for injection pre-filled syringes
86343998	Methotrexate 15mg/0.6ml solution for injection pre-filled syringes
86344998	Methotrexate 12.5mg/0.5ml solution for injection pre-filled syringes
86345998	Methotrexate 10mg/0.4ml solution for injection pre-filled syringes
86427998	Methotrexate 17.5mg/0.7ml solution for injection pre-filled syringes
86434998	Methotrexate 20mg/0.8ml solution for injection pre-filled syringes
86435998	Methotrexate 25mg/1ml solution for injection pre-filled syringes
86436998	Methotrexate 500mg/20ml solution for injection vials
86437998	Methotrexate 50mg/2ml solution for injection vials
86438998	Methotrexate 7.5mg/0.3ml solution for injection pre-filled syringes
86439998	Methotrexate 30mg/1.2ml solution for injection pre-filled syringes
86440998	Methotrexate 27.5mg/1.1ml solution for injection pre-filled syringes
86470998	Azathioprine 250mg/5ml oral solution
86519998	Azathioprine 50mg/5ml oral solution
87675998	Mercaptopurine 10mg capsules
87757998	Mercaptopurine 10mg tablets
87761998	Mesalazine 400mg gastro-resistant tablets
87859998	Adalimumab 40mg prefilled syringes
87862998	Adalimumab 40mg injection
87909998	Mesalazine 1g gastro-resistant modified-release granules sachets sugar free
87910998	Mesalazine 500mg gastro-resistant modified-release granules sachets sugar free
87911998	Mesalazine 500mg gastro-resistant modified-release granules sachets sugar free
88489998	Balsalazide 750mg capsules
88492998	Balsalazide 750mg capsules
88517998	Mesalazine 400mg gastro-resistant tablets

88519998	Sulfasalazine 500mg gastro-resistant tablets
89238998	Budesonide 2mg/100ml enema
89239998	Budesonide 2mg/100ml enema
89244979	Adalimumab 40mg prefilled syringes
89245979	Adalimumab 40mg/0.8ml solution for injection pre-filled syringes
89460998	Hydrocortisone 1% / Pramocaine 1% foam enema
89598997	Sulfasalazine 3g/100ml retention enema
89598998	Sulfasalazine 500mg suppositories
89604997	Sulfasalazine 3g/100ml retention enema
89604998	Sulfasalazine 500mg suppositories
89610997	Sulfasalazine 250mg/5ml oral suspension
89610998	Sulfasalazine 500mg tablets
89616997	Sulfasalazine 250mg/5ml oral suspension sugar free
89616998	Sulfasalazine 500mg gastro-resistant tablets
89651998	Infliximab 100mg powder for solution for infusion vials
89992997	Mesalazine 2g/59ml enema
89992998	Mesalazine 500mg suppositories
90310979	Prednisolone 20mg/application foam enema
91215998	Azathioprine 50mg tablets
91309998	Azathioprine capsules
91373997	Ciclosporin 10mg capsules
91373998	Ciclosporin 100mg/ml oral solution sugar free
91601979	Sulfasalazine 500mg gastro-resistant tablets
92244990	Ciclosporin 100mg capsules
92245990	Ciclosporin 50mg capsules
92246990	Ciclosporin 25mg capsules
92346998	Mesalazine 1g/application foam enema
92347998	Mesalazine 400mg gastro-resistant tablets
92400998	Olsalazine 500mg tablets
92401998	Olsalazine 250mg capsules
92488997	Methotrexate 2.5mg tablets

92488998	Methotrexate 5g/200ml solution for infusion vials
92511998	Infliximab 100mg powder for solution for infusion vials
92544979	Ciclosporin 50mg capsules
92552979	Ciclosporin 100mg capsules
92555979	Ciclosporin 100mg capsules
92558979	Ciclosporin 25mg capsules
92559979	Ciclosporin 25mg capsules
92566979	Azathioprine 25mg tablets
92570979	Azathioprine 25mg tablets
92571979	Azathioprine 25mg tablets
92579979	Azathioprine 50mg tablets
92639979	Methotrexate 5g/200ml solution for infusion vials
92650979	Methotrexate 200mg/8ml solution for injection vials
92655979	Methotrexate 50mg/2ml solution for injection vials
92764997	Mesalazine 1g modified-release granules sachets sugar free
92764998	Mesalazine 500mg modified-release tablets
92930998	Azathioprine 50mg tablets
92989996	Ciclosporin 100mg capsules
92989997	Ciclosporin 50mg capsules
92989998	Ciclosporin 25mg capsules
93074990	Methotrexate 2.5mg tablets
93623996	Mesalazine 250mg suppositories
93623997	Mesalazine 1g/application foam enema
93623998	Mesalazine 1g/100ml enema
93624996	Mesalazine 1g suppositories
93624997	Mesalazine 250mg modified release tablets
93624998	Mesalazine 1g/100ml enema
93728992	Mesalazine 500mg modified-release tablets
94042990	Sulfasalazine 500mg gastro-resistant tablets
94078992	Azathioprine 100 mg tab
94153998	Hydrocortisone 10% foam aerosol enema

94155997	Hydrocortisone retention enema
94155998	Hydrocortisone 10% foam aerosol enema
94308990	Azathioprine 50mg tablets
94417992	Azathioprine 50 mg sus
94437997	Olsalazine 500mg tablets
94437998	Olsalazine 250mg capsules
94438997	Olsalazine 500mg tablets
94438998	Olsalazine 250mg capsules
94451998	Prednisolone 20mg/application foam enema
94452998	Prednisolone 20mg/application foam enema
94564992	Mesalazine 500mg modified-release tablets
94593997	Ciclosporin 10mg capsules
94593998	Ciclosporin 50mg capsules
94600990	Methotrexate 20mg/0.8ml solution for injection pre-filled syringes
94690992	Azathioprine 125 mg tab
94691992	Azathioprine 10 mg tab
94697998	Azathioprine 50mg tablets
94818998	Sulfasalazine 250mg/5ml oral suspension
95041990	Mesalazine 400mg gastro-resistant tablets
95153992	Azathioprine 10mg tablets
95252990	Azathioprine 50mg tablets
95255998	Sulfasalazine 500mg gastro-resistant tablets
95256996	Sulfasalazine 3g/100ml enema
95256997	Sulfasalazine 500mg suppositories
95256998	Sulfasalazine 500mg gastro-resistant tablets
95589998	Sulfasalazine 250mg/5ml oral suspension
95725990	Azathioprine 50mg tablets
95866998	Methotrexate 25mg/ml injection
95867996	Methotrexate 5mg/2ml solution for injection vials
95867997	Methotrexate 10mg tablets
95867998	Methotrexate 2.5mg tablets

95868996	Methotrexate 50mg/3ml Injection
95868997	Methotrexate 5g/50ml solution for infusion vials
95868998	Methotrexate 200mg/8ml solution for injection vials
95869997	Methotrexate 10mg tablets
95869998	Methotrexate 2.5mg tablets
95888997	Mesalazine 250mg gastro-resistant tablets
95888998	Mesalazine 400mg gastro-resistant tablets
95890998	Mercaptopurine 50mg tablets
95891998	Mercaptopurine 50mg tablets
96177998	Hydrocortisone 1% / pramocaine 1% foam enema
96199990	Azathioprine 50mg tablets
96279990	Methotrexate 2.5mg tablets
96580998	Ciclosporin 50mg/1ml solution for infusion ampoules
96581996	Ciclosporin 100mg capsules
96581997	Ciclosporin 25mg capsules
96581998	Ciclosporin 100mg/ml oral solution sugar free
96608996	Mesalazine 2g/59ml enema
96608997	Mesalazine 1g suppositories
96608998	Mesalazine 500mg suppositories
96659996	Mesalazine 1g/application foam enema
96659997	Mesalazine 500mg suppositories
96659998	Mesalazine 250mg suppositories
96752989	Methotrexate 10mg tablets
96752990	Methotrexate 2.5mg tablets
96803990	Sulfasalazine 500mg gastro-resistant tablets
96820988	Methotrexate 5mg/2ml solution for injection vials
96883990	Mesalazine 400mg gastro-resistant tablets
96916992	Mesalazine 500mg modified-release tablets
96922989	Azathioprine 50mg tablets
96922990	Azathioprine 25mg tablets
96932998	Azathioprine 50mg powder for solution for injection vials

96933998	Azathioprine 50mg tablets
96934997	Azathioprine 50mg powder for solution for injection vials
96934998	Azathioprine 25mg tablets
97036997	Azathioprine 10mg tablets
97036998	Azathioprine 50mg tablets
97280998	Sulfasalazine 500mg gastro-resistant tablets
97281996	Sulfasalazine 3g/100ml retention enema
97281997	Sulfasalazine 500mg suppositories
97281998	Sulfasalazine 500mg tablets
97287998	Hydrocortisone acetate & pramocaine foam
97362990	Sulfasalazine 500mg gastro-resistant tablets
97363990	Sulfasalazine 500mg gastro-resistant tablets
97381998	Mesalazine 400mg gastro-resistant tablets
97719989	Sulfasalazine 500mg gastro-resistant tablets
97719990	Sulfasalazine 500mg gastro-resistant tablets
97764998	Mesalazine 250mg gastro-resistant tablets
97785990	Azathioprine 50mg tablets
98001992	Mesalazine 250mg gastro-resistant tablets
98013988	Methotrexate 5g/50ml solution for infusion vials
98211990	Azathioprine 50mg tablets
98238996	Ciclosporin 25mg capsules
98238998	Ciclosporin 100mg/ml oral solution sugar free
98365990	Sulfasalazine 500mg gastro-resistant tablets
98639990	Azathioprine 50mg tablets
98640990	Azathioprine 50mg tablets
98950997	Ciclosporin 50mg capsules
98950998	Ciclosporin 100mg capsules
98958988	Methotrexate 2.5mg tablets
98959989	Methotrexate 10mg tablets
98959990	Methotrexate 2.5mg tablets
99374990	Sulfasalazine 500mg gastro-resistant tablets

99394979	Hydrocortisone 10% foam aerosol enema
99472979	Sulfasalazine 500mg gastro-resistant tablets
99486979	Mesalazine 400mg gastro-resistant tablets
99487979	Mesalazine 400mg gastro-resistant tablets
99488979	Mesalazine 400mg gastro-resistant tablets
99490979	Mesalazine 1g modified-release granules sachets sugar free
99492979	Mesalazine 1g suppositories
99494979	Mesalazine 1g suppositories
99495979	Mesalazine 500mg modified-release tablets
99498979	Mesalazine 500mg modified-release tablets
99583996	Mesalazine 1g modified-release granules sachets sugar free
99583997	Mesalazine 500mg modified-release tablets
99583998	Mesalazine 250mg modified-release tablet
99797989	Azathioprine 25mg tablets
99797990	Azathioprine 50mg tablets
99798990	Azathioprine 50mg tablets
99799990	Azathioprine 50mg tablets
99956998	Methotrexate 5mg/2ml solution for injection vials

IBD symptoms

medcode	description
1625.00	Abnormal weight loss
1625.11	Abnormal weight loss - symptom
1627.00	Unintentional weight loss
196..11	Abdominal pain type
1962.00	Colicky abdominal pain
1963.00	Non-colicky abdominal pain
1969.00	Abdominal pain
196B.00	Painful rectal bleeding
196C.00	Painless rectal bleeding
197..13	Site of abdominal pain

1971.00	Central abdominal pain
197A.00	Generalised abdominal pain
197A.11	General abdominal pain-symptom
197B.00	Upper abdominal pain
197C.00	Lower abdominal pain
19E6.00	Blood in faeces
19E6.11	Blood in faeces symptom
19EH.00	Mucus in faeces
19F..00	Diarrhoea symptoms
19F..11	Diarrhoea
19F..12	Loose stools
19F2.00	Diarrhoea
19F3.00	Spurious (overflow) diarrhoea
19F4.00	Toddlers diarrhoea
19FZ.00	Diarrhoea symptom NOS
19FZ.11	Diarrhoea & vomiting, symptom
19G..00	Diarrhoea and vomiting
1D1A.00	Complaining of weight loss
22A8.00	Weight loss from baseline weight
4737.11	Melaena - O/E of faeces
4762.00	Faeces: fresh blood present
4762.11	Blood in faeces
4763.00	Faeces: mucous present
4764.11	Pus in faeces
A076.11	Viral diarrhoea
A082.00	Infectious diarrhoea
A082.11	Travellers' diarrhoea
A082000	Dysenteric diarrhoea
A082100	Epidemic diarrhoea
A082z00	Infectious diarrhoea NOS
A083.00	Diarrhoea of presumed infectious origin

A083.11	Diarrhoea & vomiting -? infect
Ayu0H00	[X]Diarrhoea+gastroenteritis of presumed infectious origin
E264300	Psychogenic diarrhoea
E264311	Spurious diarrhoea
Eu45317	[X]Psychogenic diarrhoea
J4...13	Noninfective diarrhoea
J432.11	Allergic diarrhoea
J433.11	Dietetic diarrhoea
J43z.11	Chronic diarrhoea
J4z..11	Presumed noninfectious diarrhoea
J4zz.11	Diarrhoea - presumed non-infectious
J521000	Irritable bowel syndrome with diarrhoea
J524000	Diarrhoea after gastrointestinal tract surgery
J525.00	Functional diarrhoea
J573011	Rectal bleeding
J573012	PRB - Rectal bleeding
J681.11	Blood in stool
J681.12	Altered blood in stools
J681.13	Blood in stools altered
L16y500	Abdominal pain in pregnancy
Q46y100	Neonatal diarrhoea
R077100	[D] Stools loose
R090.00	[D]Abdominal pain
R090400	[D]Abdominal cramps
R090E00	[D]Recurrent acute abdominal pain
R090H00	[D]Upper abdominal pain
R090N00	[D]Nonspecific abdominal pain
R090P00	[D]Functional abdominal pain syndrome
R090y00	[D]Other specified abdominal pain
R090z00	[D]Abdominal pain NOS
R121200	[D]Mucus in stool

R121300	[D]Pus in stool
Ryu1100	[X]Other and unspecified abdominal pain
1623.00	Weight decreasing
197..11	Flank pain
197..12	Iliac fossa pain
197..14	Subcostal pain
1972.00	Epigastric pain
1973.00	Left subcostal pain
1974.00	Right subcostal pain
1975.00	Left flank pain
1976.00	Right flank pain
1977.00	Right iliac fossa pain
1978.00	Left iliac fossa pain
1979.00	Suprapubic pain
197D.00	Right upper quadrant pain
19E4.12	C/O - melaena
R090000	[D]Abdominal tenderness
R090500	[D]Epigastric pain
R090600	[D]Umbilical pain
R090700	[D]Hypochondrial pain
R090800	[D]Suprapubic pain
R090900	[D]Pain in right iliac fossa
R090A00	[D]Pain in left iliac fossa
R090J00	[D]Right upper quadrant pain
R090K00	[D]Left upper quadrant pain
R090L00	[D]Left lower quadrant pain
R090M00	[D]Right lower quadrant pain
