# Supplementary material for: Incidence and prevalence of inflammatory bowel disease in UK primary care: a population-based cohort study
Source: BMJ Open. 2020 Jul 19;10(7):e036584. doi: 10.1136/bmjopen-2019-036584 (PMC7371214; doi:10.1136/bmjopen-2019-036584)
Supplement: Author's manuscript [file bmjopen-2019-036584.draft_revisions.pdf]

BMJ Open is committed to open peer review. As part of this commitment we make the peer review history of every article we publish publicly available.

When an article is published we post the peer reviewers' comments and the authors' responses online. We also post the versions of the paper that were used during peer review. These are the versions that the peer review comments apply to.

The versions of the paper that follow are the versions that were submitted during the peer review process. They are not the versions of record or the final published versions. They should not be cited or distributed as the published version of this manuscript.

BMJ Open is an open access journal and the full, final, typeset and author-corrected version of record of the manuscript is available on our site with no access controls, subscription charges or pay-per-view fees (http://bmjopen.bmj.com).

If you have any questions on BMJ Open's open peer review process please email info.bmjopen@bmj.com

BMJ Open

# Incidence and prevalence of inflammatory bowel disease in UK primary care: a population based cohort study

| | |
|---|---|
| Journal: | *BMJ Open* |
| Manuscript ID | bmjopen-2019-036584 |
| Article Type: | Original research |
| Date Submitted by the Author: | 27-Dec-2019 |
| Complete List of Authors: | Pasvol, Thomas; University College London, The Research Department of Primary Care and Population Health<br>Horsfall, Laura; University College London, Research Department of Primary Care and Population Health<br>Bloom, Stuart; University College London Hospitals NHS Foundation Trust, Medicine<br>Segal, Anthony; University College London, Medicine<br>Sabin, Caroline; University College London, Institute for Global Health; NIHR, Health Protection Research Unit in Blood Borne and Sexually Transmitted Infections<br>Field, Nigel; University College London, Centre for Molecular Epidemiology and Translational Research; University College London, Institute for Global Health<br>Rait, Greta; University College London, The Research Department of Primary Care and Population Health; NIHR, Health Protection Research Unit in Blood Borne and Sexually Transmitted Infections |
| Keywords: | Inflammatory bowel disease < GASTROENTEROLOGY, EPIDEMIOLOGY, GASTROENTEROLOGY |
| | |

SCHOLARONE™
Manuscripts

*I, the Submitting Author has the right to grant and does grant on behalf of all authors of the Work (as defined in the below author licence), an exclusive licence and/or a non-exclusive licence for contributions from authors who are: i) UK Crown employees; ii) where BMJ has agreed a CC-BY licence shall apply, and/or iii) in accordance with the terms applicable for US Federal Government officers or employees acting as part of their official duties; on a worldwide, perpetual, irrevocable, royalty-free basis to BMJ Publishing Group Ltd ("BMJ") its licensees and where the relevant Journal is co-owned by BMJ to the co-owners of the Journal, to publish the Work in this journal and any other BMJ products and to exploit all rights, as set out in our licence.*

*The Submitting Author accepts and understands that any supply made under these terms is made by BMJ to the Submitting Author unless you are acting as an employee on behalf of your employer or a postgraduate student of an affiliated institution which is paying any applicable article publishing charge ("APC") for Open Access articles. Where the Submitting Author wishes to make the Work available on an Open Access basis (and intends to pay the relevant APC), the terms of reuse of such Open Access shall be governed by a Creative Commons licence – details of these licences and which Creative Commons licence will apply to this Work are set out in our licence referred to above.*

*Other than as permitted in any relevant BMJ Author's Self Archiving Policies, I confirm this Work has not been accepted for publication elsewhere, is not being considered for publication elsewhere and does not duplicate material already published. I confirm all authors consent to publication of this Work and authorise the granting of this licence.*

For peer review only

**TITLE PAGE:**

| Title | **Incidence and prevalence of inflammatory bowel disease in UK primary care: a population based cohort study** |
|---|---|
| **Authors' names** | Thomas Joshua Pasvol (0000-0002-8334-9931), Laura Horsfall (0000-0002-0868-5660), Stuart Bloom (0000-0002-6361-4662), Anthony Walter Segal (0000-0001-7602-9043), Caroline Anne Sabin (0000-0001-5173-2760), Nigel Field (0000-0002-2825-6652), Greta Rait (0000-0002-7216-7294) |
| **Address for each author**<br>**Authors' names and positions** | Research Department of Primary Care and Population Health, University College London medical school, Royal Free Campus, Rowland Street, London, NW3 2PF, UK<br>Thomas Joshua Pasvol, Senior associate research fellow<br><br>Research Department of Primary Care and Population Health, University College London medical school, Royal Free Campus, Rowland Street, London, NW3 2PF, UK<br>Laura Horsfall, Senior epidemiologist and Wellcome Trust fellow<br><br>University College London Hospitals NHS Foundation Trust, University College Hospital, 253 Euston Road, London, NW1 2BU, UK<br>Stuart Bloom, Consultant physician and gastroenterologist<br><br>Rayne Institute, 5 University Street, London, WC1E 6JF, UK<br>Anthony Walter Segal, Charles Dent professor of medicine<br><br>Institute for Global Health, University College London, Royal Free Campus, Rowland Hill Street, London NW3 2PF, UK<br>Caroline Anne Sabin, Professor of medical statistics and epidemiology<br><br>Institute for Global Health, University College London, London, WC1E 6JB<br>Nigel Field, Infectious disease epidemiologist and public health doctor<br><br>Research Department of Primary Care and Population Health, University College London medical school, Royal Free Campus, Rowland Street, London, NW3 2PF, UK<br>Greta Rait, Professor in Primary care and health services research |
| **Corresponding author** | Correspondence to: T J Pasvol thomas.pasvol@nhs.net, +44 203 002 8787 |
| **Word count, excluding title page, abstract, references, figures and tables** | 3,608 |

For peer review only

**STRUCTURED ABSTRACT:**

**Objectives:** We describe temporal trends in the recorded incidence of inflammatory bowel disease (IBD) in UK primary care patients between 2000 and 2018.
**Design:** A cohort study.
**Setting:** The IQVIA Medical Research data (IMRD) primary care database
**Participants:** All individuals registered with GP practices contributing to IMRD during the period 01/01/2000-31/12/2018.
**Main outcome measures:** The primary outcome was the recorded diagnosis of IBD.
**Results:** 11,325,025 individuals were included and 65,700 IBD cases were identified, of which 22,560 were incident diagnoses made during the study period. Overall, there were 8,077 incident cases of Crohn's disease (CD) and 12,369 incident cases of ulcerative colitis (UC). Crude incidence estimates of 'IBD overall', CD and UC were 28.6 (28.2 to 28.9), 10.2 (10.0 to 10.5) and 15.7 (15.4 to 15.9)/100,000 person-years respectively. No change in IBD incidence was observed for adults aged 17-40 years and children aged 0-9 years. However, for adults aged over 40 years, incidence fell from 37.8 (34.5 to 41.4) to 23.6 (21.3 to 26.0)/100,000 person-years (average decrease 2.3% (1.9 to 2.7)/year ($p<0.0001$)). In adolescents aged 10-16 years, incidence rose from 13.1 (8.4 to 19.5) to 25.4 (19.5 to 32.4)/100,000 person-years (average increase 3.0% (1.7 to 4.3)/year ($p<0.0001$)). Point prevalence estimates on 31/12/2018 for 'IBD overall', CD and UC were 725, 276 and 397 per 100,000 people respectively.
**Conclusions:** This is one of the largest studies ever undertaken to investigate trends in IBD epidemiology. Although we observed stable or falling incidence of IBD in adults, our results are consistent with some of the highest reported global incidence and prevalence rates for IBD, with a 94% rise in incidence in adolescents. Further investigation is required to understand the aetiological drivers.

**KEYWORDS:** Epidemiology, gastroenterology, inflammatory bowel disease

**STRENGTHS AND LIMITATIONS OF THE STUDY:**

- Large sample size and the prospective collection of health care records representative of 'real-life' clinical practice.
- Unlike many previous IBD incidence/prevalence studies that have relied on external data sources to estimate denominator population characteristics, we were able to extract demographics and person-time follow up for all individuals in our cohort.
- Data in IMRD is recorded for patient management purposes rather than medical research. Thus, data will often only reflect those events that are deemed to be relevant to the patient's care.
- As our records were not linked to secondary care, we weren't able to confirm our cases by evidence of radiological, endoscopic or histological findings.

For peer review only

**INTRODUCTION:**

The inflammatory bowel diseases (IBD) (Crohn's disease (CD), ulcerative colitis (UC) and IBD unclassified (IBDU)) are chronic inflammatory conditions of unknown aetiology that affect the gastrointestinal tract.[1, 2] In North America, over 1.5 million individuals are living with IBD and in Europe it is estimated that 2.5-3 million individuals are affected, with an estimated direct healthcare cost of 4.6–5.6 billion Euros/year.[3, 4] Historically, IBD was regarded as a disease of high-income western countries with a substantial rise in incidence observed during the latter half of the 20$^{th}$ century.[5] However, there is evidence that the rate has plateaued in western countries while rising rapidly in newly industrialised countries.[4] Accurate and up to date estimates of trends in incidence and prevalence of IBD are an essential step in preparing services for the delivery of future IBD care.

Studies using local hospital records and secondary care databases have been conducted to describe the epidemiology of IBD in the UK.[6-9] However, patient follow-up is challenging and loss to follow-up may introduce bias, notably where patients do not require hospitalisation and/or move geographical location. More recently, estimates of incidence and prevalence of IBD were reported in a rigorously validated IBD cohort of 10,926 cases in Lothian, Scotland.[10] However, it remains unknown if these findings are generalisable across the UK.

In the UK, over 60,000,000 people are registered with a general practice (GP) (about 91% of the population).[11] Electronic GP health records databases can enable large-scale investigation of relatively rare diagnoses such as IBD.[12] The largest such UK study performed to date was undertaken in Northern England and included 179 incident cases of IBD diagnosed in a population of 135,723 during the period 1984 to 1995.[13]

In the present study, we investigated temporal trends in the incidence of IBD diagnoses from 2000 to the end of 2018 using electronic GP data from the IQVIA Medical Research Data (IMRD) primary care database (formally The Health Improvement Network (THIN) database).

For peer review only

## MATERIALS AND METHODS:

**Data source:**

IMRD is a large longitudinal database currently containing the anonymised electronic medical records of 18.3 million patients collected from 797 general practices throughout the UK; three million of these patients are presently registered with a practice contributing to IMRD and are currently providing data. It is one of the most comprehensive data sources of its kind and is used worldwide for research by academic institutions, government departments and the pharmaceutical industry. Data are based on patient consultation records and include demographics (e.g. gender, age and socio-economic level of deprivation), presenting symptoms and diagnoses, referrals to secondary care, medications, results of investigations, vaccinations and additional health data such as height, blood pressure, weight and smoking status. Data are recorded in IMRD using the Read code hierarchical coding system.[14] The GP practices in IMRD are broadly representative of all primary care practices in the UK in terms of age and sex of patients, practice size, geographical distribution and the prevalence of numerous chronic conditions such as hypertension, diabetes, asthma and epilepsy.[15] A previous validation study using electronic GP records showed that for individuals with a code for IBD, the diagnosis was highly probable or probable for 92% (95% CI 86%-96%) of patients.[12] Although IMRD data are not linked to secondary care records, diagnoses made in secondary care are captured in IMRD; either through letters and communications to the GP or during patient consultations.

**Study population:**

All data included in this study were from time periods after the GP practices had met acceptable computer usage (ACU) and acceptable mortality reporting (AMR) standards.[16, 17] The date of meeting ACU was the date after which the practice was confirmed to have an average of at least one medical record, one additional health record and two prescriptions per person-year. The date of meeting AMR standards was the date after which the practice was confirmed to record mortality at a similar rate to that expected for a population with comparable demographics as per the Office for National Statistics.

All individuals of any age contributing data between the 1st January 2000 and the 31st December 2018 were included. The study was a dynamic cohort with individuals entering and exiting at different times. Cohort entry was defined as the latest date of the following: 01/01/2000; the date of registration with the GP plus nine months to account for prevalent disease being recorded as incident disease when patients register with the practice (this time period was selected using previously published methodology);[18] or the date the practice met predefined quality indicators for electronic data (AMR and ACU). Cohort exit was defined as the earliest date of the following: first diagnosis of IBD; de-registration with the GP practice contributing data; death; or 31/12/2018.

**Main outcome definitions:**

The main outcomes of interest were newly diagnosed CD, UC or any IBD. The any IBD category included specific and general terms for IBD (comprising CD, UC, IBDU and unspecified IBD). Read code lists, adapted from those used in previous literature,[12, 13, 19] were generated for all three main outcomes using published methodology,[20] then subsequently discussed with a panel of experts including gastroenterologists, epidemiologists, a GP and a statistician (Appendix Read code lists). An algorithm was developed to assist in the validation of the diagnosis of IBD in IMRD. This involved checking whether individuals who had been given a medical Read code for IBD had a record of 1) presentation with symptoms suggestive of IBD (abdominal pain, diarrhoea, bloody stools, weight loss); 2) a prescription for a drug commonly used to treat IBD (any aminosalicylate or rectal steroid enema listed in chapter 1.5 of the British National Formulary,[21] azathioprine, mercaptopurine, methotrexate, ciclosporin, infliximab or adalimumab) (Appendix Read code lists).

As a quality filter, individuals were only included in the study as cases if they had at least two IBD Read codes recorded on separate dates or at least one IBD Read code plus at least one prescription for a drug commonly used to treat IBD. As 'incident IBD cases' (who have not been prescribed 'IBD drugs') were required to have their diagnosis verified on a subsequent GP visit, we anticipated that this would result in under ascertainment of those diagnosed in the final months of the study period. Thus, temporal trends in incidence are graphically presented for the period 2000-2017 as opposed to 2000-2018.

The date at which the first recording of any IBD code or IBD drug prescription was made was classified as the incident date. For individuals who had been given a code for both UC and CD in their lifetime, the most recent code recorded was used as their final diagnosis.

For peer review only - http://bmjopen.bmj.com/site/about/guidelines.xhtml

**Covariates:**
The following covariates were included in the analyses: 1) birth gender, 2) age by Montreal/Paris classification,[22, 23] (A classification system for IBD whereby the 'A' variable describes 'age at diagnosis', the levels of which are: A1a (0-9 years); A1b (10-17 years); A2 (17-40 years) and A3 (40+ years), 3) calendar time, 4) Townsend deprivation index (a quintile measurement of social deprivation based on post code linked census data),[24] 5) geographical location of GP practice. This was included at the level of former Strategic Health Authority (a defined region responsible for the management of health services for that particular area) for England and at the level of country for Scotland, Wales and Northern Ireland.

**Statistical analyses:**
Crude incidence estimates per 100,000 person-years at risk were calculated separately for CD, UC and any IBD with 95% confidence intervals estimated assuming a Poisson distribution. Stratified incidence rates were calculated by sex, age, Townsend deprivation index and geographical location. Time period was fitted as both a continuous variable and a categorical variable by calendar year. Mixed multivariable Poisson regression was used to estimate incidence rate ratios (IRRs). Individuals with missing data on Townsend score were included in the analysis using 'missing' as a level to the Townsend variable. General practice was included as a random effect to account for any data clustering by practice. The Wald test was used to test for significance of categorical variables in the regression model and to test for multiplicative interactions.

Point prevalence was calculated by dividing all cases of IBD (both incident and prevalent) by the total number of individuals contributing data to the cohort on the last day of the study period.

Stata 15™ was used for all analyses.

**Sensitivity analysis:**
In the sensitivity analysis we broadened our case definition to include any individual who had a single IBD medical Read code (as opposed to two medical Read codes or one medical Read code plus one relevant prescription).

**Ethics:**
IMRD data collection was approved by the NHS South-East Multicentre Research Ethics Committee in 2003. This study was approved by the Scientific Research Committee (SRC) on 29/09/2018 (SRC reference 18THIN082).

**Patient and public involvement:**
We involved representatives from the University College Hospitals NHS Foundation Trust IBD patient panel in the early stages of protocol design. However, we did not involve PPI representatives in other aspects of study design or analysis due to IMRD licence agreements and the technical computer programming methods that were involved. We intend to involve PPI representatives in writing a plain language summary for dissemination to peers and patient groups.

For peer review only

**RESULTS:**

Among the 11,325,025 individuals (78,985,977 person-years of follow up) in the cohort, we identified 65,700 cases of IBD, including 24,991 cases of CD and 36,705 cases of UC. Among these, 22,560 (8,077 for CD and 12,369 for UC) were incident diagnoses made during study follow up. Overall crude incidence estimates were 28.6 (95% CI 28.2-28.9), 10.2 (95% CI 10.0-10.5) and 15.7 (95% CI 15.4-15.9)/100,000 person-years for 'any IBD', CD and UC respectively. Point prevalence estimates on 31/12/2018 were 725, 276 and 397 per 100,000 people for 'any IBD', CD and UC respectively.

24,173/28,879 (83.7%) individuals given a new code for IBD since entering the study had a record of a prescription for a drug commonly used to treat IBD, comparing to 1.8% for the whole cohort. Additionally, 23,337/28,879 (80.8%) with a code for IBD had a record of presentation to their GP with either diarrhoea, bloody stools, abdominal pain or weight loss, comparing to 33.6% for the whole cohort.

**Table.1 Incidence rates and adjusted IRRs of CD and UC by sex, age, calendar year, geographical location and social deprivation**

| | Incidence of CD | | Incidence of UC | |
|---|---|---|---|---|
| | **Rate per 100,000 person-years (95% CI)** | **Adjusted IRR (95% CI)*** | **Rate per 100,000 person-years (95% CI)** | **Adjusted IRR (95% CI)*** |
| **Overall** | 10.2 (10.0 to 10.5) | | 15.7 (15.4 to 15.9) | |
| **Sex** | | | | |
| Male | 9.3 (0.00 ( to 9.6) | 1 | 16.7 (16.3 to 17.1) | 1 |
| Female | 11.1 (10.8 to 11.5) | 1.20 (1.15 to 1.25) | 14.7 (14.3 to 15.0) | 0.87 (0.84 to 0.90) |
| **Age , years** | | | | |
| 0-9 | 1.6 (1.3 to 1.9) | 0.11 (0.09 to 0.13) | 1.0 (0.8 to 1.3) | 0.05 (0.04 to 0.07) |
| 10-16 | 11.5 (10.7 to 12.4) | 0.77 (0.71 to 0.84) | 7.4 (6.8 to 8.1) | 0.38 (0.35 to 0.42) |
| 17-40 | 14.9 (14.4 to 15.4) | 1 | 19.2 (18.7 to 19.8) | 1 |
| 40+ | 9.2 (8.9 to 9.5) | 0.62 (0.59 to 0.64) | 17.9 (17.5 to 18.3) | 0.92 (0.89 to 0.96) |
| **Year** | | | | |
| 2000 | 10.7 (9.5 to 12.1) | 1 | 18.6 (17.0 to 20.4) | 1 |
| 2001 | 10.3 (9.2 to 11.6) | 0.97 (0.82 to 1.14) | 17.9 (16.4 to 19.5) | 0.96 (0.85 to 1.09) |
| 2002 | 10.8 (9.7 to 11.9) | 1.00 (0.85 to 1.17) | 16.7 (15.4 to 18.1) | 0.89 (0.79 to 1.01) |
| 2003 | 10.3 (9.3 to 11.4) | 0.95 (0.82 to 1.11) | 17.0 (15.8 to 18.4) | 0.91 (0.81 to 1.03) |
| 2004 | 10.2 (9.3 to 11.2) | 0.94 (0.80 to 1.09) | 17.3 (16.1 to 18.6) | 0.93 (0.82 to 1.04) |
| 2005 | 11.6 (10.6 to 12.6) | 1.07 (0.92 to 1.24) | 16.1 (14.9 to 17.3) | 0.86 (0.77 to 0.97) |
| 2006 | 11.7 (10.7 to 12.7) | 1.07 (0.93 to 1.24) | 16.3 (15.1 to 17.5) | 0.87 (0.78 to 0.98) |
| 2007 | 10.5 (9.6 to 11.4) | 0.96 (0.83 to 1.12) | 16.7 (15.5 to 17.9) | 0.89 (0.80 to 1.00) |
| 2008 | 10.6 (9.7 to 11.5) | 0.97 (0.84 to 1.13) | 17.8 (16.6 to 19.0) | 0.95 (0.85 to 1.07) |
| 2009 | 10.8 (9.9 to 11.7) | 0.99 (0.86 to 1.15) | 16.4 (15.3 to 17.6) | 0.88 (0.79 to 0.99) |
| 2010 | 10.3 (9.4 to 11.2) | 0.95 (0.82 to 1.10) | 15.6 (14.5 to 16.8) | 0.84 (0.75 to 0.94) |
| 2011 | 10.6 (9.7 to 11.5) | 0.97 (0.84 to 1.13) | 14.9 (13.8 to 16.0) | 0.80 (0.71 to 0.90) |
| 2012 | 11.1 (10.2 to 12.1) | 1.02 (0.88 to 1.18) | 15.2 (14.2 to 16.3) | 0.82 (0.73 to 0.92) |
| 2013 | 9.1 (8.3 to 10.0) | 0.84 (0.72 to 0.97) | 15.1 (14.0 to 16.2) | 0.82 (0.73 to 0.92) |
| 2014 | 10.5 (9.6 to 11.5) | 0.96 (0.82 to 1.11) | 14.5 (13.5 to 15.7) | 0.79 (0.70 to 0.89) |
| 2015 | 9.3 (8.4 to 10.3) | 0.84 (0.72 to 0.98) | 14.2 (13.0 to 15.4) | 0.77 (0.68 to 0.87) |
| 2016 | 9.0 (8.1 to 10.1) | 0.80 (0.68 to 0.95) | 13.7 (12.5 to 15.0) | 0.74 (0.65 to 0.84) |
| 2017 | 9.0 (8.0 to 10.1) | 0.79 (0.67 to 0.94) | 12.6 (11.4 to 13.9) | 0.68 (0.60 to 0.78) |
| 2018 | 5.9 (5.1 to 6.9) | 0.52 (0.43 to 0.63) | 10.0 (8.9 to 11.2) | 0.54 (0.47 to 0.63) |
| **Region** | | | | |
| East Midlands | 9.6 (8.2 to 11.2) | 1 | 17.5 (15.6 to 19.6) | 1 |
| East of England | 10.9 (10.0 to 11.9) | 1.18 (0.96 to 1.44) | 17.4 (16.2 to 18.6) | 1.01 (0.86 to 1.19) |
| London | 8.9 (8.3 to 9.6) | 0.94 (0.77 to 1.14) | 15.3 (14.5 to 16.2) | 0.94 (0.81 to 1.09) |

| | | | | |
|---|---|---|---|---|
| North East | 9.8 (8.2 to 11.6) | 1.03 (0.80 to 1.34) | 18.1 (16.0 to 20.5) | 1.08 (0.88 to 1.32) |
| North West | 11.9 (11.1 to 12.7) | 1.27 (1.05 to 1.54) | 15.8 (14.9 to 16.7) | 0.93 (0.80 to 1.08) |
| Northern Ireland | 13.1 (12.0 to 14.4) | 1.43 (1.16 to 1.75) | 16.2 (14.9 to 17.5) | 1.02 (0.87 to 1.21) |
| Scotland | 12.3 (11.7 to 12.9) | 1.36 (1.13 to 1.64) | 15.3 (14.7 to 16.0) | 0.97 (0.84 to 1.13) |
| South Central | 9.5 (8.9 to 10.2) | 1.01 (0.83 to 1.23) | 16.1 (15.2 to 17.0) | 0.92 (0.79 to 1.07) |
| South East Coast | 9.4 (8.7 to 10.1) | 1.04 (0.85 to 1.26) | 15.7 (14.9 to 16.6) | 0.95 (0.81 to 1.10) |
| South West | 10.1 (9.3 to 11.0) | 1.08 (0.89 to 1.32) | 15.2 (14.2 to 16.2) | 0.88 (0.75 to 1.03) |
| Wales | 8.9 (8.4 to 9.6) | 0.98 (0.81 to 1.19) | 14.1 (13.4 to 14.9) | 0.87 (0.75 to 1.01) |
| West Midlands | 8.4 (7.7 to 9.1) | 0.89 (0.73 to 1.09) | 16.2 (15.3 to 17.2) | 0.96 (0.82 to 1.12) |
| Yorkshire & Humber | 9.6 (8.2 to 11.2) | 1.00 (0.78 to 1.28) | 15.8 (14.0 to 17.8) | 0.91 (0.74 to 1.10) |
| **Townsend, quintile** | | | | |
| Missing | 10.1 (9.6 to 10.7) | 1.08 (0.99 to 1.17) | 15.1 (14.4 to 15.7) | 0.95 (0.89 to 1.02) |
| 1 | 9.6 (9.1 to 10.1) | 1 | 16.8 (16.2 to 17.5) | 1 |
| 2 | 10.0 (9.5 to 10.5) | 1.02 (0.95 to 1.10) | 16.5 (15.9 to 17.2) | 0.97 (0.92 to 1.03) |
| 3 | 10.7 (10.1 to 11.2) | 1.07 (1.00 to 1.15) | 16.2 (15.5 to 16.8) | 0.96 (0.91 to 1.01) |
| 4 | 10.4 (9.8 to 11.0) | 1.03 (0.96 to 1.12) | 14.8 (14.1 to 15.5) | 0.88 (0.83 to 0.94) |
| 5 | 11.2 (10.5 to 11.9) | 1.08 (0.99 to 1.17) | 13.3 (12.6 to 14.1) | 0.80 (0.74 to 0.86) |

*Adjusted for other variables considered; Sex, ageband, year, region, Townsend quintile, respectively

*Fig.1 Crude incidence estimates for any IBD, stratified by Montreal/Paris age classification and calendar year, over the period 2000-2017.*

For the period 2000-2017, incidence of ‘any IBD’ remained relatively stable for those aged 17-40 years (A2 disease) and those aged 0-9 years (A1a disease). However, for those aged over 40 years (A3 disease), incidence fell at an average rate of 2.3% (95% CI 1.9-2.7) per calendar year (p<0.0001) and for those aged 10-16 years (A1b disease), incidence rose by 3.0% (95% CI 1.7-4.3) per calendar year (p<0.0001) (Fig.1). Using the Wald test, we found an age-time interaction for all three main outcomes (p<0.00001, p=0.0046, p<0.00001 for ‘any IBD’, CD and UC respectively).

*Fig.2 Crude incidence estimates of IBD, stratified by calendar year, over the period 2000-2017.*

**CD incidence:**
During the study period, CD incidence fell slightly from 10.7 (95% CI 9.5-12.1) to 9.0 (95% CI 8.0-10.1)/100,000 person-years at an average rate of 1.0% (95% CI 0.6-1.5) per calendar year (p<0.0001) (Fig. 2). However, in children <17 years, incidence rose from 3.9 (95% CI 2.2-6.2) to 6.9 (95% CI 4.9-9.3)/100,000 person-years at an average rate of 2.9% (95% CI 1.3-4.4) per calendar year (p<0.0001) (Fig .3). Although overall crude incidence was higher for boys than for girls (7.4 (95% CI 6.8-8.0) vs 4.1 (95% CI 3.6-4.6)), a significant rise in incidence was observed for both sexes (average 2.7% (95% CI 0.8-4.6) and 3.3% (95% CI -0.6-6.0) rise per calendar year for boys and girls respectively). No change in incidence was observed for children aged 0-9 years. However, for adolescents aged 10-16 years, incidence rose from 7.6 (95% CI 4.2-12.8) to 13.1 (95% CI 9.0-18.4)/100,000 person-years at an average rate of 2.8% (1.2-4.5) per calendar year (p=0.001).

Incidence of CD was highest in Northern Ireland, Scotland and the North West (13.1 (95% CI 12.0-14.4), 12.3 (95% CI 11.7-12.9) and 11.9 (95% CI 11.1-12.7)/100,000 person-years respectively) and lowest in Wales, London and the West Midlands (8.9 (95% CI 8.4-9.6), 8.9 (95% CI 8.3-9.6) and 8.4(95% CI 7.7-9.1)/100,000 person-years respectively (Fig.4). We observed no association between social deprivation and incidence of CD after adjusting for sex, calendar year, age and geographical location (Table.1).

For peer review only

**UC incidence:**

Incidence of UC dropped to a greater extent than for CD over the study period; from 18.6 (95% CI 17.0-20.4) to 12.6 (95% CI 11.4-13.9)/100,000 person-years at an average rate of 1.6% (95% CI 1.3-2.0%) per calendar year ($p<0.0001$) (Fig.1). The fall in incidence was most pronounced for those aged over 40 years, in whom a 45% drop in incidence was observed, falling from 24.1 (95% CI 21.5-26.9) to 13.3 (95% CI 11.6-15.2)/100,000 person-years (average 3.1% (95% CI 2.6-3.6) decrease per calendar year ($p<0.0001$))

In children aged <17, incidence rose from 2.0 (95% CI 0.9-3.9) to 5.0 (95% CI 3.4-7.2)/100,000 person-years (average 2.5% (95% CI 0.5-4.4) rise per calendar year ($p=0.01$) (Fig.3). The rise in incidence was largely driven by adolescent boys aged 10-16 in whom incidence rose by 3.4% (95% CI 0.8-6.2) per calendar year ($p=0.01$). No significant change in incidence was observed in girls aged 10-16 years or children of either sex aged 0-9 years.

Incidence of UC was highest in the North East, the East of England and the East Midlands (18.1 (95% CI 16.0-20.5), 17.4 (95% CI 16.2-18.6) and 17.5 (95% CI 15.6-19.6)/100,000 person-years respectively) and lowest in Wales, the South West and London (14.1 (95% CI 13.4-14.9), 15.2 (95% CI 14.2-16.2) and 15.3 (95% CI 14.5-16.2)/100,000 person-years respectively) (Fig.4). We observed higher incidence of UC in individuals from the least deprived quintile compared to most deprived (16.8 (95% CI 16.2-17.5) vs 13.3 (95% CI 12.6-14.1)/100,000 person-years, adjusted IRR 0.80 (95% CI 0.74-0.86) (Table.1).

*Fig.3 Crude incidence estimates of IBD in children <17 years, stratified by calendar year, over the period 2000-2017.*

*Fig.4 Map showing overall crude incidence of CD and UC stratified by geographical region.*

**Sensitivity analysis:**

When broadening the case definition to include any individual who had a single IBD medical Read code, we observed overall incidence rates of 36.6 (95% CI 36.2-37.0), 12.9 (95% CI 12.7-13.2) and 19.3 (95% CI 19.0-19.6)/100,000 person-years for 'any IBD', CD and UC respectively. We observed a similar fall in incidence of UC, decreasing from 21.8 (95% CI 20.0-23.8) to 17.9 (95% 16.4-19.4)/100,000 person-years (average decrease 1.3% (95% CI 0.9-1.6) per calendar year). However, no fall in CD incidence was observed (Appendix Fig.1). When stratifying IBD incidence by five year age bands, the peak in incidence later in life was higher and occurred later than in the primary analysis (Appendix Fig.2/Fig.3).

For peer review only

**DISCUSSION:**

This is one of the largest observational studies undertaken to investigate trends in IBD epidemiology. Although incidence of IBD remained relatively stable for those aged 17-40 years and those aged 0-9 years, we observed a 38% fall in incidence for those aged over 40 years and a 94% rise in incidence in the adolescent population. The most recent incidence estimates are in line with some of the highest reported rates of paediatric IBD internationally.[25-27]

Study strengths include the large sample size and the prospective collection of health care records representative of 'real-life' clinical practice. Unlike previous incidence/prevalence studies that have relied on external data sources to estimate denominator population characteristics, we were able to extract demographics and person-time follow up for all individuals in our cohort, including those who did not develop IBD. Additionally, IMRD has been shown to be broadly representative of the UK in terms of age, sex, mortality rates and prevalence of numerous comorbidities,[15] allowing us to draw inferences from our data and relate this to the UK population as a whole. Not only has the diagnosis of IBD been validated in a similar GP database,[12] but we have demonstrated that the majority of individuals coded for IBD in IMRD have been prescribed drugs commonly used to treat IBD and presented with symptoms in keeping with IBD. This would support the argument that IMRD represents an important and useful resource for further epidemiological studies of IBD.

Limitations arise when conducting GP database research, particularly as the primary use of the software that contributes to IMRD is for patient management purposes rather than medical research. Thus, data can be incomplete and will often only reflect those events that are deemed to be relevant to the patient's care. Given that we were also reasonably strict with our case definition this may have resulted in underascertainment of cases. Although we find reason to be confident in the validity of the data, we weren't able to confirm our cases by evidence of radiological, endoscopic or histological findings. Therefore, it is possible that some individuals were misclassified.

Data from a multi-centre European study (including two UK sites in North West London and East Yorkshire) reported site incidence rates of 2.6 and 8.4 /100,000 person-years for CD and 15.9 and 8.2 /100,000 years for UC.[28] However, only a small number of UK cases were included (n=167). Incidence rates of 8.3 (3.4-13.2) and 13.9 (95% CI 7.5-20.3)/100,000 person-years for CD and UC respectively have been reported in North-East England for the period 1990-1994.[13] We report overall incidence rates of 10.2 (95% CI 10.0-10.5) and 15.6 (95% CI 15.3-15.9)/100,000 person-years for CD and UC respectively in a far larger cohort and at a national level.

A Danish study based on nationwide registry data (1995-2012) observed comparable incidence rates: 8.9 (95% CI 8.3-9.5) and 10.3 (95% CI 9.7-11.0)/100,000 person-years for CD and 23.4 (95% CI 22.4-24.5) and 23.2 (95% CI 22.2-24.3)/100,000 person-years for UC in males and females, respectively.[29] In contrast to our results, they observed overall rising incidence rates of IBD, but their study was conducted in a different country over an earlier time period including the 1990s when a rise in IBD incidence was described in many high-income countries. Although they adjusted for age in their analysis, temporal trends in incidence stratified by age group were not reported.

In the sensitivity analysis we observed higher than expected overall incidence rates. Additionally, for UC, the observed peak in incidence for older individuals was higher than the peak in incidence for younger individuals (Appendix Fig.2); this would be unusual in clinical practice. An explanation for this could be that a number of these patients, who perhaps had colitis of a different aetiology, had been misclassified as IBD. On the basis of these findings, one Read code alone was deemed not specific enough for the diagnosis of IBD.

In keeping with other UK studies we observed a rising incidence of paediatric IBD during the early 21st century,[30, 31] and we provide further evidence of male preponderance in paediatric IBD when compared to adult onset disease. Uniquely, in our study we have demonstrated rising incidence of adolescent IBD in the context of stable incidence in those aged 17-40 years and falling incidence in the over 40s. This may represent a general shift towards earlier diagnosis of IBD for all age groups except the very young (age 0-9 years). Earlier diagnosis of IBD might be explained by a number of factors, including improvements in referral pathways and the introduction of new diagnostic tools (e.g. faecal calprotectin testing or capsule endoscopy). However, one would expect step-wise increases in incidence when new diagnostic tools are rolled out, which we did not observe. Moreover, if rising incidence of adolescent IBD is due to improved referral pathways, a corresponding rise in incidence might be expected in very young children as well as adolescents. It could be argued that changes in GP coding practice may be contributing. But again, one would expect comparable changes in younger age groups if this were the case. On the

For peer review only

other hand, if the epidemiological patterns we observed reflect real increases in the incidence of pathology this is of great concern, and could represent earlier manifestation of disease related to environmental exposures in childhood and adolescence.

Our prevalence estimates were very similar to those reported in a well-validated IBD cohort in Lothian, Scotland;[10] our estimate of IBD prevalence for Scotland on 31/08/2018 was 810 per 100,000 compared to 832 per 100,000 reported by their group. Although our study lacked linkage to secondary care records, the similar prevalence estimates would support the argument that few cases were missed. In 2018, 67,150,000 people were estimated to be living in the UK from which we might extrapolate from our data that there were approximately 487,000 people living with IBD in the UK at that time.

The phenomenon of compounding prevalence of IBD has been demonstrated in Canada and in Scotland.[10, 32] This relates to the principle that although IBD incidence may be static or falling, whilst IBD mortality remains very low, prevalence will increase; In Scotland IBD prevalence is estimated to reach 1.0% by 2028. We report rising incidence rates of IBD in younger populations and falling incidence in older age groups. Thus, not only will services need to be attuned to rising IBD prevalence and an ageing demographic, but also to increasing numbers of new diagnoses in young people who will require lifelong care. This is in the context of significant financial challenges to health services.

**CONCLUSION:**

Although we observed a stable or falling incidence of IBD in adults over a 18 year period, our results are consistent with some of the highest reported global incidence and prevalence rates for IBD, with a 94% rise in incidence in adolescents. These findings are concerning and suggest detailed prospective studies are required to understand the aetiological drivers.

For peer review only

**REFERENCES:**

1. Ungaro R, Mehandru S, Allen PB, Peyrin-Biroulet L, Colombel JF. Ulcerative colitis. *Lancet (London, England)*. 2017;389(10080):1756-70.
2. Kalla R, Ventham NT, Satsangi J, Arnott ID. Crohn's disease. *BMJ (Clinical research ed)*. 2014;349:g6670.
3. Burisch J, Jess T, Martinato M, Lakatos PL. The burden of inflammatory bowel disease in Europe. *Journal of Crohn's & colitis*. 2013;7(4):322-37.
4. Ng SC, Shi HY, Hamidi N, et al. Worldwide incidence and prevalence of inflammatory bowel disease in the 21st century: a systematic review of population-based studies. *Lancet (London, England)*. 2018;390(10114):2769-78.
5. Whelan G. Epidemiology of inflammatory bowel disease. *The Medical clinics of North America*. 1990;74(1):1-12.
6. Ashton JJ, Wiskin AE, Ennis S, et al. Rising incidence of paediatric inflammatory bowel disease (PIBD) in Wessex, Southern England. *Archives of disease in childhood*. 2014;99(7):659-64.
7. Steed H, Walsh S, Reynolds N. Crohn's disease incidence in NHS Tayside. *Scottish medical journal*. 2010;55(3):22-5.
8. Stone MA, Mayberry JF, Baker R. Prevalence and management of inflammatory bowel disease: a cross-sectional study from central England. *European journal of gastroenterology & hepatology*. 2003;15(12):1275-80.
9. Hassan K, Cowan FJ, Jenkins HR. The incidence of childhood inflammatory bowel disease in Wales. *European journal of pediatrics*. 2000;159(4):261-3.
10. Jones G-R, Lyons M, Plevris N, et al. IBD prevalence in Lothian, Scotland, derived by capture–recapture methodology. *Gut*. 2019:gutjnl-2019-318936.
11. NHS Digital. Patients Registered at a GP Practice September 2019 2019 [Available from: https://digital.nhs.uk/data-and-information/publications/statistical/patients-registered-at-a-gp-practice/september-2019.
12. Lewis JD, Brensinger C, Bilker WB, Strom BL. Validity and completeness of the General Practice Research Database for studies of inflammatory bowel disease. *Pharmacoepidemiology and drug safety*. 2002;11(3):211-8.
13. Rubin GP, Hungin AP, Kelly PJ, Ling J. Inflammatory bowel disease: epidemiology and management in an English general practice population. *Alimentary pharmacology & therapeutics*. 2000;14(12):1553-9.
14. Benson T. The history of the Read Codes: the inaugural James Read Memorial Lecture 2011. *Informatics in primary care*. 2011;19(3):173-82.
15. Blak BT, Thompson M, Dattani H, Bourke A. Generalisability of The Health Improvement Network (THIN) database: demographics, chronic disease prevalence and mortality rates. *Informatics in primary care*. 2011;19(4):251-5.
16. Horsfall L, Walters K, Petersen I. Identifying periods of acceptable computer usage in primary care research databases. *Pharmacoepidemiology and drug safety*. 2013;22(1):64-9.
17. Maguire A, Blak BT, Thompson M. The importance of defining periods of complete mortality reporting for research using automated data from primary care. *Pharmacoepidemiology and drug safety*. 2009;18(1):76-83.
18. Lewis JD, Bilker WB, Weinstein RB, Strom BL. The relationship between time since registration and measured incidence rates in the General Practice Research Database. *Pharmacoepidemiology and drug safety*. 2005;14(7):443-51.
19. Abrahami D, Douros A, Yin H, et al. Dipeptidyl peptidase-4 inhibitors and incidence of inflammatory bowel disease among patients with type 2 diabetes: population based cohort study. *BMJ (Clinical research ed)*. 2018;360:k872.
20. Dave S, Petersen I. Creating medical and drug code lists to identify cases in primary care databases. *Pharmacoepidemiology and drug safety*. 2009;18(8):704-7.
21. Committee JF. British National Formulary. 76 ed. London: Pharmaceutical Press; 2018.
22. Satsangi J, Silverberg MS, Vermeire S, Colombel JF. The Montreal classification of inflammatory bowel disease: controversies, consensus, and implications. *Gut*. 2006;55(6):749-53.
23. Levine A, Griffiths A, Markowitz J, et al. Pediatric modification of the Montreal classification for inflammatory bowel disease: the Paris classification. *Inflammatory bowel diseases*. 2011;17(6):1314-21.

For peer review only

24. Townsend P PPBA. Health and deprivation. Inequality and the North. London: Croom Helm. *Health Policy*. 1988;10(2):207-6.
25. Malmborg P, Grahnquist L, Lindholm J, Montgomery S, Hildebrand H. Increasing incidence of paediatric inflammatory bowel disease in northern Stockholm County, 2002-2007. *Journal of pediatric gastroenterology and nutrition*. 2013;57(1):29-34.
26. Benchimol EI, Guttmann A, Griffiths AM, et al. Increasing incidence of paediatric inflammatory bowel disease in Ontario, Canada: evidence from health administrative data. *Gut*. 2009;58(11):1490-7.
27. Perminow G, Brackmann S, Lyckander LG, et al. A characterization in childhood inflammatory bowel disease, a new population-based inception cohort from South-Eastern Norway, 2005-07, showing increased incidence in Crohn's disease. *Scandinavian journal of gastroenterology*. 2009;44(4):446-56.
28. Burisch J, Pedersen N, Cukovic-Cavka S, et al. East-West gradient in the incidence of inflammatory bowel disease in Europe: the ECCO-EpiCom inception cohort. *Gut*. 2014;63(4):588-97.
29. Norgard BM, Nielsen J, Fonager K, et al. The incidence of ulcerative colitis (1995-2011) and Crohn's disease (1995-2012) - based on nationwide Danish registry data. *Journal of Crohn's & colitis*. 2014;8(10):1274-80.
30. Ashton JJ, Cullen M, Afzal NA, et al. Is the incidence of paediatric inflammatory bowel disease still increasing? *Archives of disease in childhood*. 2018;103(11):1093-4.
31. Henderson P, Hansen R, Cameron FL, et al. Rising incidence of pediatric inflammatory bowel disease in Scotland. *Inflammatory bowel diseases*. 2012;18(6):999-1005.
32. Kaplan GG, Bernstein CN, Coward S, et al. The Impact of Inflammatory Bowel Disease in Canada 2018: Epidemiology. *Journal of the Canadian Association of Gastroenterology*. 2019;2(Suppl 1):S6-s16.

For peer review only

**FUNDING:**
This work was supported by The Charles Wolfson Charitable Trust [grant number 539234] and The Harbour Foundation [grant number 549321]. Neither funder had any role in study design; in the collection, analysis, and interpretation of data; in the writing of the report; and in the decision to submit the article for publication. The lead author confirms the independence of researchers from funders and that all authors, external and internal, had full access to all of the data (including statistical reports and tables) in the study and can take responsibility for the integrity of the data and the accuracy of the data analysis.

**TRANSPARENCY STATEMENT:**
The lead author affirms that this manuscript is an honest, accurate, and transparent account of the study being reported; that no important aspects of the study have been omitted; and that any discrepancies from the study as originally planned have been explained.

**COMPETING INTERESTS DECLARATION:**
All authors have completed the ICMJE uniform disclosure form at www.icmje.org/coi_disclosure.pdf and declare: TJP and AWS report research grants from The Charles Wolfson Charitable Trust and The Harbour Foundation for the submitted work; no financial relationships with any organisations that might have an interest in the submitted work in the previous three years; no other relationships or activities that could appear to have influenced the submitted work.

**CONTRIBUTOR AND GUARANTOR INFORMATION:**
**T J Pasvol** - study concept and design; analysis and interpretation of data; drafting of the manuscript; statistical analysis; obtained funding.
**L Horsfall** - study concept and design; analysis and interpretation of data; critical revision of the manuscript for important intellectual content; statistical analysis.
**S Bloom** - study concept and design; critical revision of the manuscript for important intellectual content.
**A W Segal** - study concept and design; critical revision of the manuscript for important intellectual content; obtained funding.
**C A Sabin** - study concept and design; critical revision of the manuscript for important intellectual content; statistical analysis.
**N Field** - study concept and design; critical revision of the manuscript for important intellectual content.
**G Rait** - study concept and design; analysis and interpretation of data; critical revision of the manuscript for important intellectual content; study supervision.

The corresponding author attests that all listed authors meet authorship criteria and that no others meeting the criteria have been omitted.

**COPYRIGHT/LICENSE FOR PUBLICATION:**
The Corresponding Author has the right to grant on behalf of all authors and does grant on behalf of all authors, a worldwide licence to the Publishers and its licensees in perpetuity, in all forms, formats and media (whether known now or created in the future), to i) publish, reproduce, distribute, display and store the Contribution, ii) translate the Contribution into other languages, create adaptations, reprints, include within collections and create summaries, extracts and/or, abstracts of the Contribution, iii) create any other derivative work(s) based on the Contribution, iv) to exploit all subsidiary rights in the Contribution, v) the inclusion of electronic links from the Contribution to third party material where-ever it may be located; and, vi) licence any third party to do any or all of the above.

**DATA SHARING:**
Due to licence agreements with IQVIA, we are unable to share patient level data from the IQVIA Medical Research Database. However, we are happy to share our data extractions upon reasonable request. Data requesters should email the corresponding author to request the relevant data.

For peer review only

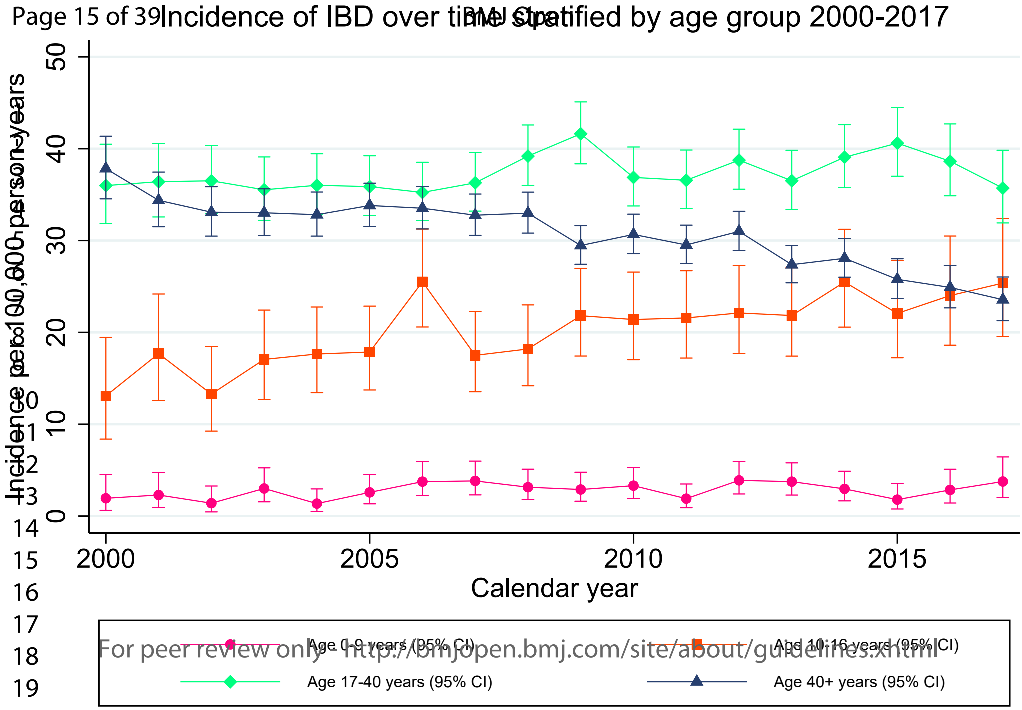


For peer review only - http://bmjopen.bmj.com/site/about/guidelines.xhtml

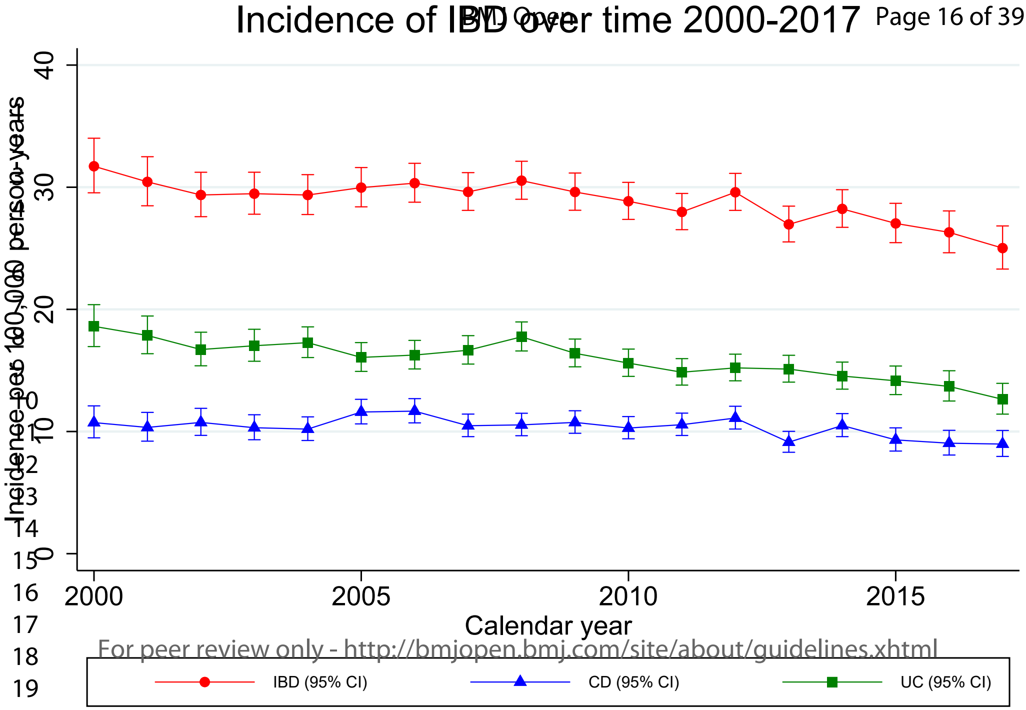


For peer review only - http://bmjopen.bmj.com/site/about/guidelines.xhtml

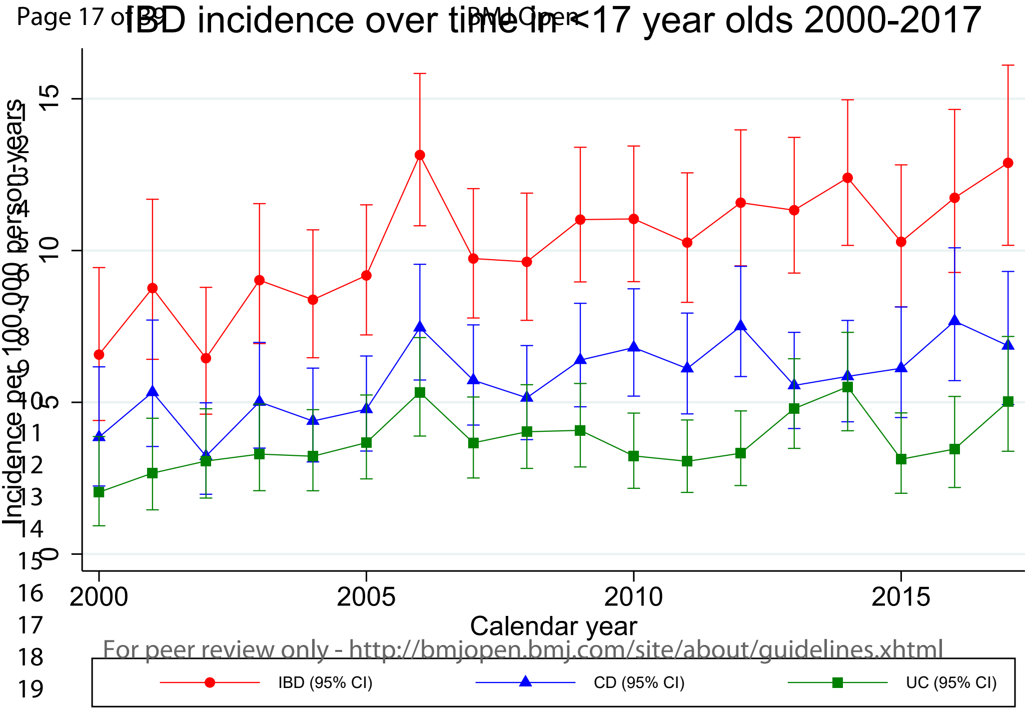


For peer review only - http://bmjopen.bmj.com/site/about/guidelines.xhtml

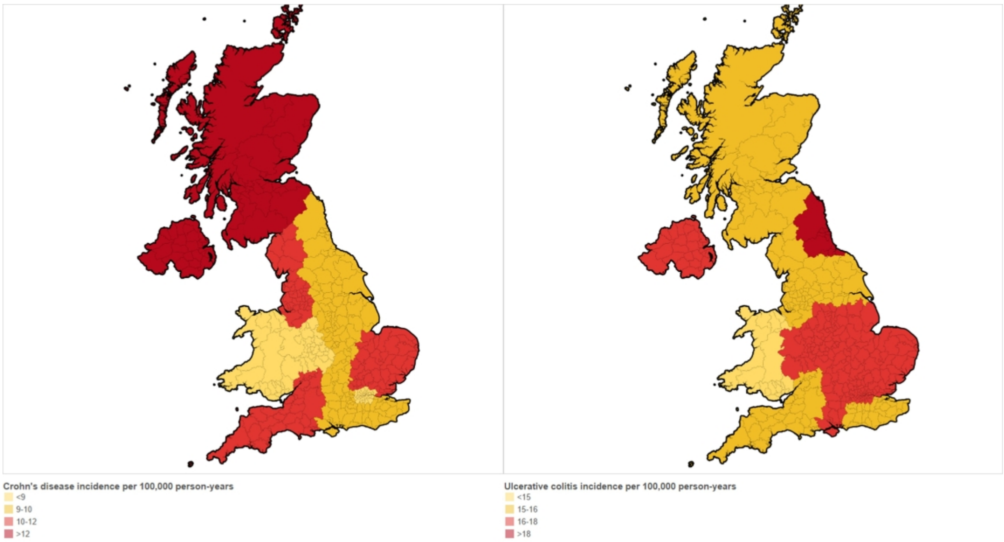


Fig.4 Map showing overall crude incidence of CD and UC stratified by geographical region

338x190mm (300 x 300 DPI)

**APPENDIX:**

*Appendix fig.1 Crude incidence estimates of IBD, stratified by calendar year, over the period 2000-2017 (sensitivity analysis)*

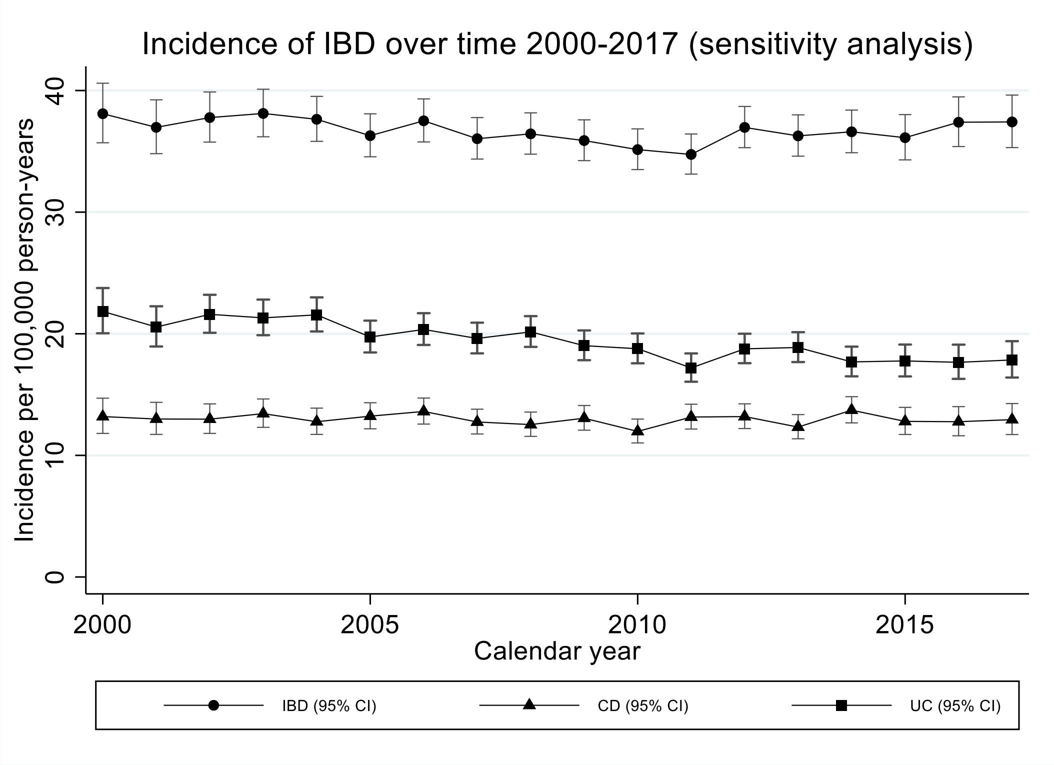

*Appendix fig.2 Crude incidence estimates of IBD by five year age bands (primary analysis)*

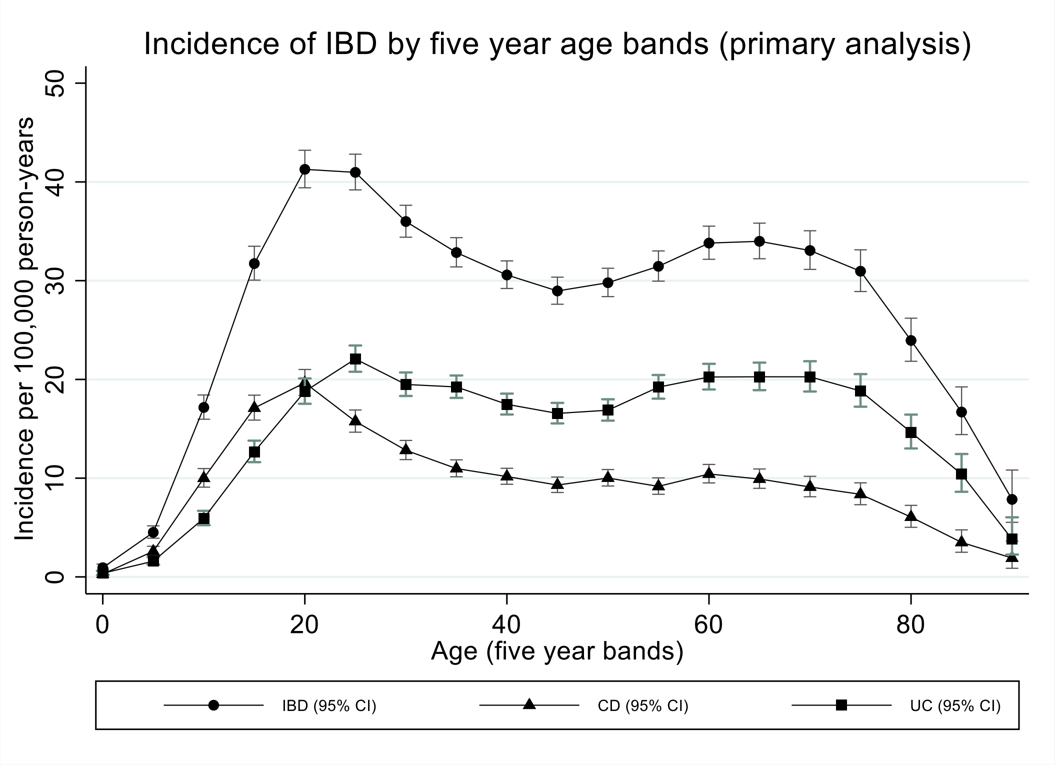


*Appendix fig.3 Crude incidence estimates of IBD by five year age bands (sensitivity analysis)*

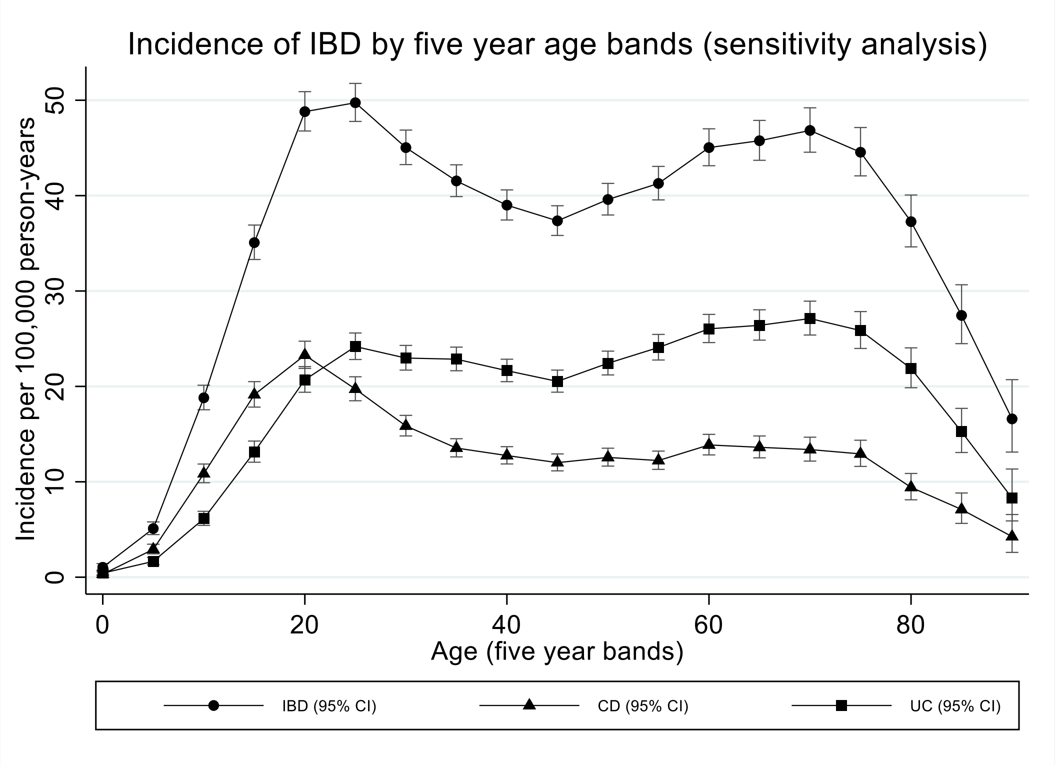


Read code lists:

*Any IBD*

| medcode | description |
|---|---|
| 14C4.11 | H/O: ulcerative colitis |
| 8Cc5.00 | Management of inflammatory bowel disease |
| 8Cc5.11 | Management of IBD (inflammatory bowel disease) |
| J08z900 | Orofacial Crohn's disease |
| J4...12 | Inflammatory bowel disease |
| J40..00 | Regional enteritis - Crohn's disease |
| J40..11 | Crohn's disease |
| J400.00 | Regional enteritis of the small bowel |
| J400000 | Regional enteritis of the duodenum |
| J400100 | Regional enteritis of the jejunum |
| J400200 | Crohn's disease of the terminal ileum |
| J400300 | Crohn's disease of the ileum unspecified |
| J400400 | Crohn's disease of the ileum NOS |
| J400500 | Exacerbation of Crohn's disease of small intestine |
| J400z00 | Crohn's disease of the small bowel NOS |
| J401.00 | Regional enteritis of the large bowel |
| J401000 | Regional enteritis of the colon |
| J401100 | Regional enteritis of the rectum |
| J401200 | Exacerbation of Crohn's disease of large intestine |
| J401z00 | Crohn's disease of the large bowel NOS |
| J401z11 | Crohn's colitis |
| J402.00 | Regional ileocolitis |
| J40z.00 | Regional enteritis NOS |
| J40z.11 | Crohn's disease NOS |
| J41..12 | Ulcerative colitis and/or proctitis |
| J410.00 | Ulcerative proctocolitis |
| J410100 | Ulcerative colitis |
| J410200 | Ulcerative rectosigmoiditis |
| J410300 | Ulcerative proctitis |
| J410400 | Exacerbation of ulcerative colitis |

For peer review only

| | |
|---|---|
| J410z00 | Ulcerative proctocolitis NOS |
| J413.00 | Ulcerative pancolitis |
| J41y.00 | Other idiopathic proctocolitis |
| J41yz00 | Other idiopathic proctocolitis NOS |
| J41z.00 | Idiopathic proctocolitis NOS |
| J4z6.00 | Indeterminate colitis |
| Jyu4000 | [X]Other Crohn's disease |
| Jyu4100 | [X]Other ulcerative colitis |

*CD*

| **medcode** | **description** |
|---|---|
| J08z900 | Orofacial Crohn's disease |
| J40..00 | Regional enteritis - Crohn's disease |
| J40..11 | Crohn's disease |
| J400.00 | Regional enteritis of the small bowel |
| J400000 | Regional enteritis of the duodenum |
| J400100 | Regional enteritis of the jejunum |
| J400200 | Crohn's disease of the terminal ileum |
| J400300 | Crohn's disease of the ileum unspecified |
| J400400 | Crohn's disease of the ileum NOS |
| J400500 | Exacerbation of Crohn's disease of small intestine |
| J400z00 | Crohn's disease of the small bowel NOS |
| J401.00 | Regional enteritis of the large bowel |
| J401000 | Regional enteritis of the colon |
| J401100 | Regional enteritis of the rectum |
| J401200 | Exacerbation of Crohn's disease of large intestine |
| J401z00 | Crohn's disease of the large bowel NOS |
| J401z11 | Crohn's colitis |
| J402.00 | Regional ileocolitis |
| J40z.00 | Regional enteritis NOS |
| J40z.11 | Crohn's disease NOS |

For peer review only

| | |
|---|---|
| Jyu4000 | [X]Other Crohn's disease |

*UC*

| **medcode** | **description** |
|---|---|
| 14C4.11 | H/O: ulcerative colitis |
| J41..12 | Ulcerative colitis and/or proctitis |
| J410.00 | Ulcerative proctocolitis |
| J410100 | Ulcerative colitis |
| J410200 | Ulcerative rectosigmoiditis |
| J410300 | Ulcerative proctitis |
| J410400 | Exacerbation of ulcerative colitis |
| J410z00 | Ulcerative proctocolitis NOS |
| J413.00 | Ulcerative pancolitis |
| J41y.00 | Other idiopathic proctocolitis |
| J41yz00 | Other idiopathic proctocolitis NOS |
| J41z.00 | Idiopathic proctocolitis NOS |
| Jyu4100 | [X]Other ulcerative colitis |

*Drugs related to IBD*

| **drugcode** | **genericname** |
|---|---|
| 30104978 | Adalimumab 40mg/0.4ml solution for injection pre-filled syringes |
| 30105978 | Adalimumab 40mg/0.4ml solution for injection pre-filled syringes |
| 30106978 | Adalimumab 40mg/0.4ml solution for injection pre-filled disposable devices |
| 53189979 | Ciclosporin 50mg capsules |
| 53190979 | Ciclosporin 100mg capsules |
| 53191979 | Ciclosporin 25mg capsules |
| 53913979 | Mesalazine 800mg gastro-resistant tablets |
| 54552979 | Mesalazine 400mg gastro-resistant tablets |
| 55164978 | Mesalazine 4g modified-release granules sachets sugar free |
| 55165978 | Mesalazine 4g modified-release granules sachets sugar free |
| 55193978 | Mercaptopurine 75mg tablets |

For peer review only

| | |
|---|---|
| 55575979 | Ciclosporin 50mg capsules |
| 55576979 | Ciclosporin 100mg capsules |
| 55577979 | Ciclosporin 25mg capsules |
| 55799978 | Mercaptopurine 30mg capsules |
| 56901978 | Ciclosporin 100mg capsules |
| 56902978 | Ciclosporin 25mg capsules |
| 58121979 | Mercaptopurine 25mg tablets |
| 58800979 | Mesalazine 1.2g gastro-resistant modified-release tablets |
| 60097979 | Prednisolone 20mg/application foam enema |
| 60124979 | Ciclosporin 50mg capsules |
| 60584979 | Mesalazine 3g gastro-resistant modified-release granules sachets sugar free |
| 60585979 | Mesalazine 3g gastro-resistant modified-release granules sachets sugar free |
| 61544979 | Adalimumab 40mg/0.8ml solution for injection vials |
| 61545979 | Adalimumab 40mg/0.8ml solution for injection vials |
| 62953979 | Sulfasalazine 250mg/5ml oral suspension sugar free |
| 64115979 | Methotrexate 25mg/3ml solution for injection pre-filled syringes |
| 64868979 | Mesalazine 2g modified-release granules sachets sugar free |
| 65098979 | Sulfasalazine 500mg/5ml oral suspension |
| 67089979 | Mesalazine 1.2g gastro-resistant modified-release tablets |
| 69316979 | Azathioprine 125mg/5ml oral suspension |
| 70274978 | Azathioprine 50mg tablets |
| 70879979 | Adalimumab 40mg prefilled pen |
| 70880979 | Adalimumab 40mg/0.8ml solution for injection pre-filled disposable devices |
| 72688978 | Ciclosporin 50mg capsules |
| 73065978 | Methotrexate 7.5mg/0.15ml solution for injection pre-filled disposable devices |
| 73066978 | Methotrexate 7.5mg/0.15ml solution for injection pre-filled disposable devices |
| 73067978 | Methotrexate 30mg/0.6ml solution for injection pre-filled disposable devices |
| 73068978 | Methotrexate 30mg/0.6ml solution for injection pre-filled disposable devices |
| 73069978 | Methotrexate 27.5mg/0.55ml solution for injection pre-filled disposable devices |
| 73071978 | Methotrexate 25mg/0.5ml solution for injection pre-filled disposable devices |
| 73072978 | Methotrexate 25mg/0.5ml solution for injection pre-filled disposable devices |

For peer review only

| | |
|---|---|
| 73073978 | Methotrexate 22.5mg/0.45ml solution for injection pre-filled disposable devices |
| 73074978 | Methotrexate 22.5mg/0.45ml solution for injection pre-filled disposable devices |
| 73075978 | Methotrexate 20mg/0.4ml solution for injection pre-filled disposable devices |
| 73076978 | Methotrexate 20mg/0.4ml solution for injection pre-filled disposable devices |
| 73077978 | Methotrexate 17.5mg/0.35ml solution for injection pre-filled disposable devices |
| 73078978 | Methotrexate 17.5mg/0.35ml solution for injection pre-filled disposable devices |
| 73079978 | Methotrexate 15mg/0.3ml solution for injection pre-filled disposable devices |
| 73080978 | Methotrexate 15mg/0.3ml solution for injection pre-filled disposable devices |
| 73081978 | Methotrexate 12.5mg/0.25ml solution for injection pre-filled disposable devices |
| 73082978 | Methotrexate 12.5mg/0.25ml solution for injection pre-filled disposable devices |
| 73083978 | Methotrexate 10mg/0.2ml solution for injection pre-filled disposable devices |
| 73084978 | Methotrexate 10mg/0.2ml solution for injection pre-filled disposable devices |
| 76424978 | Mesalazine 1g modified-release tablets |
| 76878978 | Methotrexate 2.5mg tablets |
| 78442979 | Methotrexate 50mg/2ml solution for injection vials |
| 78447979 | Methotrexate 25mg/1ml solution for injection pre-filled syringes |
| 78449979 | Methotrexate 20mg/0.8ml solution for injection pre-filled syringes |
| 78452979 | Methotrexate 12.5mg/0.5ml solution for injection pre-filled syringes |
| 79522979 | Mercaptopurine 25mg/5ml oral suspension |
| 79524979 | Mercaptopurine 20mg/ml oral suspension |
| 79739978 | Sulfasalazine 500mg gastro-resistant tablets |
| 79867978 | Mesalazine 400mg gastro-resistant tablets |
| 80925979 | Azathioprine 25mg/5ml oral suspension |
| 80927979 | Azathioprine 25mg/5ml oral solution |
| 80928998 | Mesalazine 3g gastro-resistant modified-release granules sachets sugar free |
| 80929979 | Azathioprine 20mg/5ml oral suspension |
| 80929998 | Mesalazine 3g gastro-resistant modified-release granules sachets sugar free |
| 80961979 | Azathioprine 10mg/5ml oral suspension |
| 81193998 | Mesalazine 1g modified-release tablets |
| 81194998 | Mesalazine 1g modified-release tablets |
| 81282998 | Ciclosporin 100mg capsules |

For peer review only

| | |
|---|---|
| 81283998 | Ciclosporin 50mg capsules |
| 81284998 | Ciclosporin 25mg capsules |
| 81490998 | Methotrexate 27.5mg/0.55ml prefilled syringes |
| 81491998 | Methotrexate 22.5mg/0.45ml prefilled syringes |
| 81492998 | Methotrexate 17.5mg/0.35ml prefilled syringes |
| 81493998 | Methotrexate 12.5mg/0.25ml prefilled syringes |
| 81494998 | Methotrexate 27.5mg/0.55ml solution for injection pre-filled syringes |
| 81495998 | Methotrexate 22.5mg/0.45ml solution for injection pre-filled syringes |
| 81496998 | Methotrexate 17.5mg/0.35ml solution for injection pre-filled syringes |
| 81498998 | Methotrexate 12.5mg/0.25ml solution for injection pre-filled syringes |
| 81638998 | Methotrexate 30mg/1.5ml solution for injection pre-filled syringes |
| 81640998 | Methotrexate 25mg/1.25ml solution for injection pre-filled syringes |
| 81642998 | Methotrexate 20mg/1ml solution for injection pre-filled syringes |
| 81643998 | Methotrexate 7.5mg/0.75ml prefilled syringes |
| 81683998 | Mesalazine 1g suppositories |
| 81689998 | Mesalazine 500mg gastro-resistant tablets |
| 81690998 | Mesalazine 500mg gastro-resistant tablets |
| 81771998 | Ciclosporin 25mg capsules |
| 81772998 | Mesalazine 800mg gastro-resistant tablets |
| 81773998 | Ciclosporin 100mg capsules |
| 81774998 | Ciclosporin 50mg capsules |
| 81815998 | Methotrexate 30mg/0.6ml prefilled syringes |
| 81816998 | Methotrexate 30mg/0.6ml solution for injection pre-filled syringes |
| 81868998 | Mesalazine 400mg gastro-resistant tablets |
| 82203998 | Azathioprine 50mg tablets |
| 82204998 | Azathioprine 25mg tablets |
| 82480998 | Ciclosporin 100mg capsules |
| 82481998 | Ciclosporin 50mg capsules |
| 82482998 | Ciclosporin 25mg capsules |
| 82840998 | Methotrexate 25mg/0.5ml prefilled syringes |
| 82841998 | Methotrexate 20mg/0.4ml prefilled syringes |

For peer review only

| | |
|---|---|
| 82842998 | Methotrexate 15mg/0.3ml prefilled syringes |
| 82843998 | Methotrexate 10mg/0.2ml prefilled syringes |
| 82844998 | Methotrexate 7.5mg/0.15ml prefilled syringes |
| 82845998 | Methotrexate 25mg/0.5ml solution for injection pre-filled syringes |
| 82846998 | Methotrexate 20mg/0.4ml solution for injection pre-filled syringes |
| 82847998 | Methotrexate 15mg/0.3ml solution for injection pre-filled syringes |
| 82848998 | Methotrexate 10mg/0.2ml solution for injection pre-filled syringes |
| 82849998 | Methotrexate 7.5mg/0.15ml solution for injection pre-filled syringes |
| 83228978 | Ciclosporin 50mg capsules |
| 83229978 | Ciclosporin 100mg capsules |
| 83485998 | Azathioprine 10mg capsules |
| 83503998 | Mesalazine 1.5g gastro-resistant modified-release granules sachets sugar free |
| 83504998 | Mesalazine 1.5g gastro-resistant modified-release granules sachets sugar free |
| 83559978 | Mercaptopurine 50mg tablets |
| 83743998 | Mesalazine 2g modified-release granules sachets sugar free |
| 83769998 | Sulfasalazine 250mg/5ml oral suspension sugar free |
| 83987998 | Mesalazine 2g modified-release granules sachets sugar free |
| 84059998 | Mesalazine 1g modified-release granules sachets sugar free |
| 84209998 | Mesalazine 800mg gastro-resistant tablets |
| 84290998 | Mesalazine 1.2g gastro-resistant modified-release tablets |
| 84291998 | Mesalazine 1.2g gastro-resistant modified-release tablets |
| 84438998 | Methotrexate 2mg/ml oral solution sugar free |
| 84439998 | Methotrexate 10mg/5ml oral suspension |
| 84636998 | Budesonide 2mg foam enema |
| 84637998 | Budesonide 2mg foam enema |
| 84741998 | Prednisolone 40mg/100ml enema |
| 84920998 | Ciclosporin 250mg/5ml solution for infusion ampoules |
| 84921998 | Ciclosporin 50mg/1ml solution for infusion ampoules |
| 84927998 | Ciclosporin 250mg/5ml solution for infusion ampoules |
| 84928998 | Ciclosporin 50mg/1ml solution for infusion ampoules |
| 85097998 | Methotrexate 1g/40ml solution for injection vials |

For peer review only

| | |
|---|---|
| 85100998 | Methotrexate 7.5mg/5ml oral suspension |
| 85188978 | Methotrexate 2.5mg tablets |
| 85252998 | Azathioprine 50mg/5ml oral suspension |
| 85556998 | Mercaptopurine oral solution |
| 85560998 | Mesalazine 800mg gastro-resistant tablets |
| 85639998 | Methotrexate 7.5mg/0.75ml prefilled syringes |
| 85640998 | Methotrexate 10mg/ml prefilled syringes |
| 85641998 | Methotrexate 15mg/1.5ml prefilled syringes |
| 85642998 | Methotrexate 20mg/2ml prefilled syringes |
| 85643998 | Methotrexate 25mg/2.5ml prefilled syringes |
| 85644998 | Methotrexate 7.5mg/0.75ml solution for injection pre-filled syringes |
| 85645998 | Methotrexate 10mg/1ml solution for injection pre-filled syringes |
| 85646998 | Methotrexate 15mg/1.5ml solution for injection pre-filled syringes |
| 85648998 | Methotrexate 20mg/2ml solution for injection pre-filled syringes |
| 85650998 | Methotrexate 25mg/2.5ml solution for injection pre-filled syringes |
| 85737998 | Methotrexate 5g/50ml solution for infusion vials |
| 85738998 | Methotrexate 1g/10ml solution for injection vials |
| 85776998 | Methotrexate oral solution |
| 85777998 | Methotrexate 12.5mg/5ml oral suspension |
| 86327998 | Methotrexate 2.5mg/5ml oral suspension |
| 86339998 | Methotrexate 5mg/0.2ml solution for injection pre-filled syringes |
| 86342998 | Methotrexate 22.5mg/0.9ml solution for injection pre-filled syringes |
| 86343998 | Methotrexate 15mg/0.6ml solution for injection pre-filled syringes |
| 86344998 | Methotrexate 12.5mg/0.5ml solution for injection pre-filled syringes |
| 86345998 | Methotrexate 10mg/0.4ml solution for injection pre-filled syringes |
| 86427998 | Methotrexate 17.5mg/0.7ml solution for injection pre-filled syringes |
| 86434998 | Methotrexate 20mg/0.8ml solution for injection pre-filled syringes |
| 86435998 | Methotrexate 25mg/1ml solution for injection pre-filled syringes |
| 86436998 | Methotrexate 500mg/20ml solution for injection vials |
| 86437998 | Methotrexate 50mg/2ml solution for injection vials |
| 86438998 | Methotrexate 7.5mg/0.3ml solution for injection pre-filled syringes |

For peer review only

| | |
|---|---|
| 86439998 | Methotrexate 30mg/1.2ml solution for injection pre-filled syringes |
| 86440998 | Methotrexate 27.5mg/1.1ml solution for injection pre-filled syringes |
| 86470998 | Azathioprine 250mg/5ml oral solution |
| 86519998 | Azathioprine 50mg/5ml oral solution |
| 87675998 | Mercaptopurine 10mg capsules |
| 87757998 | Mercaptopurine 10mg tablets |
| 87761998 | Mesalazine 400mg gastro-resistant tablets |
| 87859998 | Adalimumab 40mg prefilled syringes |
| 87862998 | Adalimumab 40mg injection |
| 87909998 | Mesalazine 1g gastro-resistant modified-release granules sachets sugar free |
| 87910998 | Mesalazine 500mg gastro-resistant modified-release granules sachets sugar free |
| 87911998 | Mesalazine 500mg gastro-resistant modified-release granules sachets sugar free |
| 88489998 | Balsalazide 750mg capsules |
| 88492998 | Balsalazide 750mg capsules |
| 88517998 | Mesalazine 400mg gastro-resistant tablets |
| 88519998 | Sulfasalazine 500mg gastro-resistant tablets |
| 89238998 | Budesonide 2mg/100ml enema |
| 89239998 | Budesonide 2mg/100ml enema |
| 89244979 | Adalimumab 40mg prefilled syringes |
| 89245979 | Adalimumab 40mg/0.8ml solution for injection pre-filled syringes |
| 89460998 | Hydrocortisone 1% / Pramocaine 1% foam enema |
| 89598997 | Sulfasalazine 3g/100ml retention enema |
| 89598998 | Sulfasalazine 500mg suppositories |
| 89604997 | Sulfasalazine 3g/100ml retention enema |
| 89604998 | Sulfasalazine 500mg suppositories |
| 89610997 | Sulfasalazine 250mg/5ml oral suspension |
| 89610998 | Sulfasalazine 500mg tablets |
| 89616997 | Sulfasalazine 250mg/5ml oral suspension sugar free |
| 89616998 | Sulfasalazine 500mg gastro-resistant tablets |
| 89651998 | Infliximab 100mg powder for solution for infusion vials |
| 89992997 | Mesalazine 2g/59ml enema |

For peer review only

| | |
|---|---|
| 89992998 | Mesalazine 500mg suppositories |
| 90310979 | Prednisolone 20mg/application foam enema |
| 91215998 | Azathioprine 50mg tablets |
| 91309998 | Azathioprine capsules |
| 91373997 | Ciclosporin 10mg capsules |
| 91373998 | Ciclosporin 100mg/ml oral solution sugar free |
| 91601979 | Sulfasalazine 500mg gastro-resistant tablets |
| 92244990 | Ciclosporin 100mg capsules |
| 92245990 | Ciclosporin 50mg capsules |
| 92246990 | Ciclosporin 25mg capsules |
| 92346998 | Mesalazine 1g/application foam enema |
| 92347998 | Mesalazine 400mg gastro-resistant tablets |
| 92400998 | Olsalazine 500mg tablets |
| 92401998 | Olsalazine 250mg capsules |
| 92488997 | Methotrexate 2.5mg tablets |
| 92488998 | Methotrexate 5g/200ml solution for infusion vials |
| 92511998 | Infliximab 100mg powder for solution for infusion vials |
| 92544979 | Ciclosporin 50mg capsules |
| 92552979 | Ciclosporin 100mg capsules |
| 92555979 | Ciclosporin 100mg capsules |
| 92558979 | Ciclosporin 25mg capsules |
| 92559979 | Ciclosporin 25mg capsules |
| 92566979 | Azathioprine 25mg tablets |
| 92570979 | Azathioprine 25mg tablets |
| 92571979 | Azathioprine 25mg tablets |
| 92579979 | Azathioprine 50mg tablets |
| 92639979 | Methotrexate 5g/200ml solution for infusion vials |
| 92650979 | Methotrexate 200mg/8ml solution for injection vials |
| 92655979 | Methotrexate 50mg/2ml solution for injection vials |
| 92764997 | Mesalazine 1g modified-release granules sachets sugar free |
| 92764998 | Mesalazine 500mg modified-release tablets |

For peer review only

| | |
|---|---|
| 92930998 | Azathioprine 50mg tablets |
| 92989996 | Ciclosporin 100mg capsules |
| 92989997 | Ciclosporin 50mg capsules |
| 92989998 | Ciclosporin 25mg capsules |
| 93074990 | Methotrexate 2.5mg tablets |
| 93623996 | Mesalazine 250mg suppositories |
| 93623997 | Mesalazine 1g/application foam enema |
| 93623998 | Mesalazine 1g/100ml enema |
| 93624996 | Mesalazine 1g suppositories |
| 93624997 | Mesalazine 250mg modified release tablets |
| 93624998 | Mesalazine 1g/100ml enema |
| 93728992 | Mesalazine 500mg modified-release tablets |
| 94042990 | Sulfasalazine 500mg gastro-resistant tablets |
| 94078992 | Azathioprine 100 mg tab |
| 94153998 | Hydrocortisone 10% foam aerosol enema |
| 94155997 | Hydrocortisone retention enema |
| 94155998 | Hydrocortisone 10% foam aerosol enema |
| 94308990 | Azathioprine 50mg tablets |
| 94417992 | Azathioprine 50 mg sus |
| 94437997 | Olsalazine 500mg tablets |
| 94437998 | Olsalazine 250mg capsules |
| 94438997 | Olsalazine 500mg tablets |
| 94438998 | Olsalazine 250mg capsules |
| 94451998 | Prednisolone 20mg/application foam enema |
| 94452998 | Prednisolone 20mg/application foam enema |
| 94564992 | Mesalazine 500mg modified-release tablets |
| 94593997 | Ciclosporin 10mg capsules |
| 94593998 | Ciclosporin 50mg capsules |
| 94600990 | Methotrexate 20mg/0.8ml solution for injection pre-filled syringes |
| 94690992 | Azathioprine 125 mg tab |
| 94691992 | Azathioprine 10 mg tab |

For peer review only

| | |
|---|---|
| 94697998 | Azathioprine 50mg tablets |
| 94818998 | Sulfasalazine 250mg/5ml oral suspension |
| 95041990 | Mesalazine 400mg gastro-resistant tablets |
| 95153992 | Azathioprine 10mg tablets |
| 95252990 | Azathioprine 50mg tablets |
| 95255998 | Sulfasalazine 500mg gastro-resistant tablets |
| 95256996 | Sulfasalazine 3g/100ml enema |
| 95256997 | Sulfasalazine 500mg suppositories |
| 95256998 | Sulfasalazine 500mg gastro-resistant tablets |
| 95589998 | Sulfasalazine 250mg/5ml oral suspension |
| 95725990 | Azathioprine 50mg tablets |
| 95866998 | Methotrexate 25mg/ml injection |
| 95867996 | Methotrexate 5mg/2ml solution for injection vials |
| 95867997 | Methotrexate 10mg tablets |
| 95867998 | Methotrexate 2.5mg tablets |
| 95868996 | Methotrexate 50mg/3ml Injection |
| 95868997 | Methotrexate 5g/50ml solution for infusion vials |
| 95868998 | Methotrexate 200mg/8ml solution for injection vials |
| 95869997 | Methotrexate 10mg tablets |
| 95869998 | Methotrexate 2.5mg tablets |
| 95888997 | Mesalazine 250mg gastro-resistant tablets |
| 95888998 | Mesalazine 400mg gastro-resistant tablets |
| 95890998 | Mercaptopurine 50mg tablets |
| 95891998 | Mercaptopurine 50mg tablets |
| 96177998 | Hydrocortisone 1% / pramocaine 1% foam enema |
| 96199990 | Azathioprine 50mg tablets |
| 96279990 | Methotrexate 2.5mg tablets |
| 96580998 | Ciclosporin 50mg/1ml solution for infusion ampoules |
| 96581996 | Ciclosporin 100mg capsules |
| 96581997 | Ciclosporin 25mg capsules |
| 96581998 | Ciclosporin 100mg/ml oral solution sugar free |

For peer review only

| | |
|---|---|
| 96608996 | Mesalazine 2g/59ml enema |
| 96608997 | Mesalazine 1g suppositories |
| 96608998 | Mesalazine 500mg suppositories |
| 96659996 | Mesalazine 1g/application foam enema |
| 96659997 | Mesalazine 500mg suppositories |
| 96659998 | Mesalazine 250mg suppositories |
| 96752989 | Methotrexate 10mg tablets |
| 96752990 | Methotrexate 2.5mg tablets |
| 96803990 | Sulfasalazine 500mg gastro-resistant tablets |
| 96820988 | Methotrexate 5mg/2ml solution for injection vials |
| 96883990 | Mesalazine 400mg gastro-resistant tablets |
| 96916992 | Mesalazine 500mg modified-release tablets |
| 96922989 | Azathioprine 50mg tablets |
| 96922990 | Azathioprine 25mg tablets |
| 96932998 | Azathioprine 50mg powder for solution for injection vials |
| 96933998 | Azathioprine 50mg tablets |
| 96934997 | Azathioprine 50mg powder for solution for injection vials |
| 96934998 | Azathioprine 25mg tablets |
| 97036997 | Azathioprine 10mg tablets |
| 97036998 | Azathioprine 50mg tablets |
| 97280998 | Sulfasalazine 500mg gastro-resistant tablets |
| 97281996 | Sulfasalazine 3g/100ml retention enema |
| 97281997 | Sulfasalazine 500mg suppositories |
| 97281998 | Sulfasalazine 500mg tablets |
| 97287998 | Hydrocortisone acetate & pramocaine foam |
| 97362990 | Sulfasalazine 500mg gastro-resistant tablets |
| 97363990 | Sulfasalazine 500mg gastro-resistant tablets |
| 97381998 | Mesalazine 400mg gastro-resistant tablets |
| 97719989 | Sulfasalazine 500mg gastro-resistant tablets |
| 97719990 | Sulfasalazine 500mg gastro-resistant tablets |
| 97764998 | Mesalazine 250mg gastro-resistant tablets |

For peer review only

| | |
|---|---|
| 97785990 | Azathioprine 50mg tablets |
| 98001992 | Mesalazine 250mg gastro-resistant tablets |
| 98013988 | Methotrexate 5g/50ml solution for infusion vials |
| 98211990 | Azathioprine 50mg tablets |
| 98238996 | Ciclosporin 25mg capsules |
| 98238998 | Ciclosporin 100mg/ml oral solution sugar free |
| 98365990 | Sulfasalazine 500mg gastro-resistant tablets |
| 98639990 | Azathioprine 50mg tablets |
| 98640990 | Azathioprine 50mg tablets |
| 98950997 | Ciclosporin 50mg capsules |
| 98950998 | Ciclosporin 100mg capsules |
| 98958988 | Methotrexate 2.5mg tablets |
| 98959989 | Methotrexate 10mg tablets |
| 98959990 | Methotrexate 2.5mg tablets |
| 99374990 | Sulfasalazine 500mg gastro-resistant tablets |
| 99394979 | Hydrocortisone 10% foam aerosol enema |
| 99472979 | Sulfasalazine 500mg gastro-resistant tablets |
| 99486979 | Mesalazine 400mg gastro-resistant tablets |
| 99487979 | Mesalazine 400mg gastro-resistant tablets |
| 99488979 | Mesalazine 400mg gastro-resistant tablets |
| 99490979 | Mesalazine 1g modified-release granules sachets sugar free |
| 99492979 | Mesalazine 1g suppositories |
| 99494979 | Mesalazine 1g suppositories |
| 99495979 | Mesalazine 500mg modified-release tablets |
| 99498979 | Mesalazine 500mg modified-release tablets |
| 99583996 | Mesalazine 1g modified-release granules sachets sugar free |
| 99583997 | Mesalazine 500mg modified-release tablets |
| 99583998 | Mesalazine 250mg modified-release tablet |
| 99797989 | Azathioprine 25mg tablets |
| 99797990 | Azathioprine 50mg tablets |
| 99798990 | Azathioprine 50mg tablets |

For peer review only

| | |
|---|---|
| 99799990 | Azathioprine 50mg tablets |
| 99956998 | Methotrexate 5mg/2ml solution for injection vials |

*IBD symptoms*

| **medcode** | **description** |
|---|---|
| 1625.00 | Abnormal weight loss |
| 1625.11 | Abnormal weight loss - symptom |
| 1627.00 | Unintentional weight loss |
| 196..11 | Abdominal pain type |
| 1962.00 | Colicky abdominal pain |
| 1963.00 | Non-colicky abdominal pain |
| 1969.00 | Abdominal pain |
| 196B.00 | Painful rectal bleeding |
| 196C.00 | Painless rectal bleeding |
| 197..13 | Site of abdominal pain |
| 1971.00 | Central abdominal pain |
| 197A.00 | Generalised abdominal pain |
| 197A.11 | General abdominal pain-symptom |
| 197B.00 | Upper abdominal pain |
| 197C.00 | Lower abdominal pain |
| 19E6.00 | Blood in faeces |
| 19E6.11 | Blood in faeces symptom |
| 19EH.00 | Mucus in faeces |
| 19F..00 | Diarrhoea symptoms |
| 19F..11 | Diarrhoea |
| 19F..12 | Loose stools |
| 19F2.00 | Diarrhoea |
| 19F3.00 | Spurious (overflow) diarrhoea |
| 19F4.00 | Toddlers diarrhoea |
| 19FZ.00 | Diarrhoea symptom NOS |
| 19FZ.11 | Diarrhoea & vomiting, symptom |

For peer review only

| | |
|---|---|
| 19G..00 | Diarrhoea and vomiting |
| 1D1A.00 | Complaining of weight loss |
| 22A8.00 | Weight loss from baseline weight |
| 4737.11 | Melaena - O/E of faeces |
| 4762.00 | Faeces: fresh blood present |
| 4762.11 | Blood in faeces |
| 4763.00 | Faeces: mucous present |
| 4764.11 | Pus in faeces |
| A076.11 | Viral diarrhoea |
| A082.00 | Infectious diarrhoea |
| A082.11 | Travellers' diarrhoea |
| A082000 | Dysenteric diarrhoea |
| A082100 | Epidemic diarrhoea |
| A082z00 | Infectious diarrhoea NOS |
| A083.00 | Diarrhoea of presumed infectious origin |
| A083.11 | Diarrhoea & vomiting -? infect |
| Ayu0H00 | [X]Diarrhoea+gastroenteritis of presumed infectious origin |
| E264300 | Psychogenic diarrhoea |
| E264311 | Spurious diarrhoea |
| Eu45317 | [X]Psychogenic diarrhoea |
| J4...13 | Noninfective diarrhoea |
| J432.11 | Allergic diarrhoea |
| J433.11 | Dietetic diarrhoea |
| J43z.11 | Chronic diarrhoea |
| J4z..11 | Presumed noninfectious diarrhoea |
| J4zz.11 | Diarrhoea - presumed non-infectious |
| J521000 | Irritable bowel syndrome with diarrhoea |
| J524000 | Diarrhoea after gastrointestinal tract surgery |
| J525.00 | Functional diarrhoea |
| J573011 | Rectal bleeding |
| J573012 | PRB - Rectal bleeding |

For peer review only

| | |
|---|---|
| J681.11 | Blood in stool |
| J681.12 | Altered blood in stools |
| J681.13 | Blood in stools altered |
| L16y500 | Abdominal pain in pregnancy |
| Q46y100 | Neonatal diarrhoea |
| R077100 | [D] Stools loose |
| R090.00 | [D]Abdominal pain |
| R090400 | [D]Abdominal cramps |
| R090E00 | [D]Recurrent acute abdominal pain |
| R090H00 | [D]Upper abdominal pain |
| R090N00 | [D]Nonspecific abdominal pain |
| R090P00 | [D]Functional abdominal pain syndrome |
| R090y00 | [D]Other specified abdominal pain |
| R090z00 | [D]Abdominal pain NOS |
| R121200 | [D]Mucus in stool |
| R121300 | [D]Pus in stool |
| Ryu1100 | [X]Other and unspecified abdominal pain |
| 1623.00 | Weight decreasing |
| 197..11 | Flank pain |
| 197..12 | Iliac fossa pain |
| 197..14 | Subcostal pain |
| 1972.00 | Epigastric pain |
| 1973.00 | Left subcostal pain |
| 1974.00 | Right subcostal pain |
| 1975.00 | Left flank pain |
| 1976.00 | Right flank pain |
| 1977.00 | Right iliac fossa pain |
| 1978.00 | Left iliac fossa pain |
| 1979.00 | Suprapubic pain |
| 197D.00 | Right upper quadrant pain |
| 19E4.12 | C/O - melaena |

For peer review only

| | |
|---|---|
| R090000 | [D]Abdominal tenderness |
| R090500 | [D]Epigastric pain |
| R090600 | [D]Umbilical pain |
| R090700 | [D]Hypochondrial pain |
| R090800 | [D]Suprapubic pain |
| R090900 | [D]Pain in right iliac fossa |
| R090A00 | [D]Pain in left iliac fossa |
| R090J00 | [D]Right upper quadrant pain |
| R090K00 | [D]Left upper quadrant pain |
| R090L00 | [D]Left lower quadrant pain |
| R090M00 | [D]Right lower quadrant pain |

For peer review only

STROBE Statement—Checklist of items that should be included in reports of ***cohort studies***

| | Item No | Recommendation | Page No |
|---|---|---|---|
| **Title and abstract** | 1 | (*a*) Indicate the study's design with a commonly used term in the title or the abstract<br>(*b*) Provide in the abstract an informative and balanced summary of what was done and what was found | 1,5 |
| **Introduction** | | | |
| Background/rationale | 2 | Explain the scientific background and rationale for the investigation being reported | 6 |
| Objectives | 3 | State specific objectives, including any prespecified hypotheses | 6 |
| **Methods** | | | |
| Study design | 4 | Present key elements of study design early in the paper | 7 |
| Setting | 5 | Describe the setting, locations, and relevant dates, including periods of recruitment, exposure, follow-up, and data collection | 7 |
| Participants | 6 | (*a*) Give the eligibility criteria, and the sources and methods of selection of participants. Describe methods of follow-up<br>(*b*) For matched studies, give matching criteria and number of exposed and unexposed | 7 |
| Variables | 7 | Clearly define all outcomes, exposures, predictors, potential confounders, and effect modifiers. Give diagnostic criteria, if applicable | 7,8 |
| Data sources/ measurement | 8* | For each variable of interest, give sources of data and details of methods of assessment (measurement). Describe comparability of assessment methods if there is more than one group | 7 |
| Bias | 9 | Describe any efforts to address potential sources of bias | 8 |
| Study size | 10 | Explain how the study size was arrived at | 7 |
| Quantitative variables | 11 | Explain how quantitative variables were handled in the analyses. If applicable, describe which groupings were chosen and why | 8 |
| Statistical methods | 12 | (*a*) Describe all statistical methods, including those used to control for confounding<br>(*b*) Describe any methods used to examine subgroups and interactions<br>(*c*) Explain how missing data were addressed<br>(*d*) If applicable, explain how loss to follow-up was addressed<br>(*e*) Describe any sensitivity analyses | 8 |
| **Results** | | | |
| Participants | 13* | (a) Report numbers of individuals at each stage of study—eg numbers potentially eligible, examined for eligibility, confirmed eligible, included in the study, completing follow-up, and analysed<br>(b) Give reasons for non-participation at each stage<br>(c) Consider use of a flow diagram | 9 |
| Descriptive data | 14* | (a) Give characteristics of study participants (eg demographic, clinical, social) and information on exposures and potential confounders<br>(b) Indicate number of participants with missing data for each variable of interest<br>(c) Summarise follow-up time (eg, average and total amount) | 9 |
| Outcome data | 15* | Report numbers of outcome events or summary measures over time | 9,10,11 |

For peer review only


For peer review only - http://bmjopen.bmj.com/site/about/guidelines.xhtml

| | | | |
|---|---|---|---|
| Main results | 16 | (*a*) Give unadjusted estimates and, if applicable, confounder-adjusted estimates and their precision (eg, 95% confidence interval). Make clear which confounders were adjusted for and why they were included<br>(*b*) Report category boundaries when continuous variables were categorized<br>(*c*) If relevant, consider translating estimates of relative risk into absolute risk for a meaningful time period | 9,10,11 |
| Other analyses | 17 | Report other analyses done—eg analyses of subgroups and interactions, and sensitivity analyses | 11 |
| **Discussion** | | | |
| Key results | 18 | Summarise key results with reference to study objectives | 12 |
| Limitations | 19 | Discuss limitations of the study, taking into account sources of potential bias or imprecision. Discuss both direction and magnitude of any potential bias | 12 |
| Interpretation | 20 | Give a cautious overall interpretation of results considering objectives, limitations, multiplicity of analyses, results from similar studies, and other relevant evidence | 12,13 |
| Generalisability | 21 | Discuss the generalisability (external validity) of the study results | 12 |
| **Other information** | | | |
| Funding | 22 | Give the source of funding and the role of the funders for the present study and, if applicable, for the original study on which the present article is based | 14 |

*Give information separately for exposed and unexposed groups.

**Note:** An Explanation and Elaboration article discusses each checklist item and gives methodological background and published examples of transparent reporting. The STROBE checklist is best used in conjunction with this article (freely available on the Web sites of PLoS Medicine at http://www.plosmedicine.org/, Annals of Internal Medicine at http://www.annals.org/, and Epidemiology at http://www.epidem.com/). Information on the STROBE Initiative is available at http://www.strobe-statement.org.

For peer review only

BMJ Open

# Incidence and prevalence of inflammatory bowel disease in UK primary care: a population based cohort study

| | |
|---|---|
| Journal: | *BMJ Open* |
| Manuscript ID | bmjopen-2019-036584.R1 |
| Article Type: | Original research |
| Date Submitted by the Author: | 20-May-2020 |
| Complete List of Authors: | Pasvol, Thomas; University College London, The Research Department of Primary Care and Population Health<br>Horsfall, Laura; University College London, Research Department of Primary Care and Population Health<br>Bloom, Stuart; University College London Hospitals NHS Foundation Trust, Medicine<br>Segal, Anthony; University College London, Medicine<br>Sabin, Caroline; University College London, Institute for Global Health; NIHR, Health Protection Research Unit in Blood Borne and Sexually Transmitted Infections<br>Field, Nigel; University College London, Centre for Molecular Epidemiology and Translational Research; University College London, Institute for Global Health<br>Rait, Greta; University College London, The Research Department of Primary Care and Population Health; NIHR, Health Protection Research Unit in Blood Borne and Sexually Transmitted Infections |
| <b>Primary Subject Heading</b>: | Gastroenterology and hepatology |
| Secondary Subject Heading: | Epidemiology |
| Keywords: | Inflammatory bowel disease < GASTROENTEROLOGY, EPIDEMIOLOGY, GASTROENTEROLOGY |
| | |

SCHOLARONE™
Manuscripts

*I, the Submitting Author has the right to grant and does grant on behalf of all authors of the Work (as defined in the below author licence), an exclusive licence and/or a non-exclusive licence for contributions from authors who are: i) UK Crown employees; ii) where BMJ has agreed a CC-BY licence shall apply, and/or iii) in accordance with the terms applicable for US Federal Government officers or employees acting as part of their official duties; on a worldwide, perpetual, irrevocable, royalty-free basis to BMJ Publishing Group Ltd ("BMJ") its licensees and where the relevant Journal is co-owned by BMJ to the co-owners of the Journal, to publish the Work in this journal and any other BMJ products and to exploit all rights, as set out in our licence.*

*The Submitting Author accepts and understands that any supply made under these terms is made by BMJ to the Submitting Author unless you are acting as an employee on behalf of your employer or a postgraduate student of an affiliated institution which is paying any applicable article publishing charge ("APC") for Open Access articles. Where the Submitting Author wishes to make the Work available on an Open Access basis (and intends to pay the relevant APC), the terms of reuse of such Open Access shall be governed by a Creative Commons licence – details of these licences and which Creative Commons licence will apply to this Work are set out in our licence referred to above.*

*Other than as permitted in any relevant BMJ Author's Self Archiving Policies, I confirm this Work has not been accepted for publication elsewhere, is not being considered for publication elsewhere and does not duplicate material already published. I confirm all authors consent to publication of this Work and authorise the granting of this licence.*

For peer review only

**TITLE PAGE:**

| | |
|---|---|
| **Title** | **Incidence and prevalence of inflammatory bowel disease in UK primary care: a population based cohort study** |
| **Authors' names** | Thomas Joshua Pasvol (0000-0002-8334-9931), Laura Horsfall (0000-0002-0868-5660), Stuart Bloom (0000-0002-6361-4662), Anthony Walter Segal (0000-0001-7602-9043), Caroline Anne Sabin (0000-0001-5173-2760), Nigel Field (0000-0002-2825-6652), Greta Rait (0000-0002-7216-7294) |
| **Address for each author**<br>**Authors' names and positions** | Research Department of Primary Care and Population Health, University College London medical school, Royal Free Campus, Rowland Street, London, NW3 2PF, UK<br>Thomas Joshua Pasvol, Senior associate research fellow<br><br>Research Department of Primary Care and Population Health, University College London medical school, Royal Free Campus, Rowland Street, London, NW3 2PF, UK<br>Laura Horsfall, Senior epidemiologist and Wellcome Trust fellow<br><br>University College London Hospitals NHS Foundation Trust, University College Hospital, 253 Euston Road, London, NW1 2BU, UK<br>Stuart Bloom, Consultant physician and gastroenterologist<br><br>Rayne Institute, 5 University Street, London, WC1E 6JF, UK<br>Anthony Walter Segal, Charles Dent professor of medicine<br><br>Institute for Global Health, University College London, Royal Free Campus, Rowland Hill Street, London NW3 2PF, UK<br>Caroline Anne Sabin, Professor of medical statistics and epidemiology<br><br>Institute for Global Health, University College London, London, WC1E 6JB<br>Nigel Field, Infectious disease epidemiologist and public health doctor<br><br>Research Department of Primary Care and Population Health, University College London medical school, Royal Free Campus, Rowland Street, London, NW3 2PF, UK<br>Greta Rait, Professor in Primary care and health services research |
| **Corresponding author** | Correspondence to: T J Pasvol thomas.pasvol@nhs.net, +44 203 002 8787 |
| **Word count, excluding title page, abstract, references, figures and tables** | 3,608 |

For peer review only

**STRUCTURED ABSTRACT:**

**Objectives:** We describe temporal trends in the recorded incidence of inflammatory bowel disease (IBD) in UK primary care patients between 2000 and 2018.
**Design:** A cohort study.
**Setting:** The IQVIA Medical Research data (IMRD) primary care database
**Participants:** All individuals registered with GP practices contributing to IMRD during the period 01/01/2000-31/12/2018.
**Main outcome measures:** The primary outcome was the recorded diagnosis of IBD.
**Results:** 11,325,025 individuals were included and 65,700 IBD cases were identified, of which 22,560 were incident diagnoses made during the study period. Overall, there were 8,077 incident cases of Crohn's disease (CD) and 12,369 incident cases of ulcerative colitis (UC). Crude incidence estimates of 'IBD overall', CD and UC were 28.6 (28.2 to 28.9), 10.2 (10.0 to 10.5) and 15.7 (15.4 to 15.9)/100,000 person-years respectively. No change in IBD incidence was observed for adults aged 17-40 years and children aged 0-9 years. However, for adults aged over 40 years, incidence fell from 37.8 (34.5 to 41.4) to 23.6 (21.3 to 26.0)/100,000 person-years (average decrease 2.3% (1.9 to 2.7)/year ($p<0.0001$)). In adolescents aged 10-16 years, incidence rose from 13.1 (8.4 to 19.5) to 25.4 (19.5 to 32.4)/100,000 person-years (average increase 3.0% (1.7 to 4.3)/year ($p<0.0001$)). Point prevalence estimates on 31/12/2018 for 'IBD overall', CD and UC were 725, 276 and 397 per 100,000 people respectively.
**Conclusions:** This is one of the largest studies ever undertaken to investigate trends in IBD epidemiology. Although we observed stable or falling incidence of IBD in adults, our results are consistent with some of the highest reported global incidence and prevalence rates for IBD, with a 94% rise in incidence in adolescents. Further investigation is required to understand the aetiological drivers.

**KEYWORDS:** Epidemiology, gastroenterology, inflammatory bowel disease

**STRENGTHS AND LIMITATIONS OF THE STUDY:**

- We used a large sample size
- The data source was prospectively collected health care records representative of 'real-life' clinical practice.
- Unlike many previous IBD incidence/prevalence studies that have relied on external data sources to estimate denominator population characteristics, we were able to extract demographics and person-time follow up for all individuals in our cohort.
- Data in IMRD is recorded for patient management purposes rather than medical research.
- As our records were not linked to secondary care, we weren't able to confirm our cases by evidence of radiological, endoscopic or histological findings.

For peer review only

## INTRODUCTION:

The inflammatory bowel diseases (IBD) (Crohn's disease (CD), ulcerative colitis (UC) and IBD unclassified (IBDU)) are chronic inflammatory conditions of unknown aetiology that affect the gastrointestinal tract.[1, 2] In North America, over 1.5 million individuals are living with IBD and in Europe it is estimated that 2.5-3 million individuals are affected, with an estimated direct healthcare cost of 4.6–5.6 billion Euros/year.[3, 4] Historically, IBD was regarded as a disease of high-income western countries with a substantial rise in incidence observed during the latter half of the 20$^{th}$ century.[5] However, there is evidence that the rate has plateaued in western countries while rising rapidly in newly industrialised countries.[4] Accurate and up to date estimates of trends in incidence and prevalence of IBD are an essential step in preparing services for the delivery of future IBD care.

Studies using local hospital records and secondary care databases have been conducted to describe the epidemiology of IBD in the UK.[6-9] However, patient follow-up is challenging and loss to follow-up may introduce bias, notably where patients do not require hospitalisation and/or move geographical location. More recently, estimates of incidence and prevalence of IBD were reported in a rigorously validated IBD cohort of 10,926 cases in Lothian, Scotland.[10] However, it remains unknown if these findings are generalisable across the UK.

In the UK, over 60,000,000 people are registered with a general practice (GP) (about 91% of the population).[11] Electronic GP health records databases can enable large-scale investigation of relatively rare diagnoses such as IBD.[12] The largest such UK study performed to date was undertaken in Northern England and included 179 incident cases of IBD diagnosed in a population of 135,723 during the period 1984 to 1995.[13]

In the present study, we investigated temporal trends in the incidence of IBD diagnoses from 2000 to the end of 2018 using electronic GP data from the IQVIA Medical Research Data (IMRD) primary care database (formally The Health Improvement Network (THIN) database).

For peer review only

**MATERIALS AND METHODS:**

**Data source:**

IMRD is a large longitudinal database currently containing the anonymised electronic medical records of 18.3 million patients collected from 797 general practices throughout the UK; three million of these patients are presently registered with a practice contributing to IMRD and are currently providing data. It is one of the most comprehensive data sources of its kind and is used worldwide for research by academic institutions, government departments and the pharmaceutical industry. Data are based on patient consultation records and include demographics (e.g. gender, age and socio-economic level of deprivation), presenting symptoms and diagnoses, referrals to secondary care, medications, results of investigations, vaccinations and additional health data such as height, blood pressure, weight and smoking status. Data are recorded in IMRD using the Read code hierarchical coding system.[14] No other coding system was used in IMRD for the duration of the observation period. The GP practices in IMRD are broadly representative of all primary care practices in the UK in terms of age and sex of patients, practice size, geographical distribution and the prevalence of numerous chronic conditions such as hypertension, diabetes, asthma and epilepsy.[15] A previous validation study using electronic GP records showed that for individuals with a code for IBD, the diagnosis was highly probable or probable for 92% (95% CI 86%-96%) of patients.[12] Although IMRD data are not linked to secondary care records, diagnoses made in secondary care are captured in IMRD; either through letters and communications to the GP or during patient consultations.

**Study population:**

All data included in this study were from time periods after the GP practices had met acceptable computer usage (ACU) and acceptable mortality reporting (AMR) standards.[16, 17] The date of meeting ACU was the date after which the practice was confirmed to have electronically logged an average of at least one medical record, one additional health record and two prescriptions per person-year. The date of meeting AMR standards was the date after which the practice was confirmed to record mortality at a similar rate to that expected for a population with comparable demographics as per the Office for National Statistics.

All individuals of any age contributing data between the 1st January 2000 and the 31st December 2018 were included. The study was a dynamic cohort with individuals entering and exiting at different times. Cohort entry was defined as the latest date of the following: 01/01/2000; the date of registration with the GP plus nine months to account for prevalent disease being recorded as incident disease when patients register with the practice (this time period was selected using previously published methodology);[18] or the date the practice met predefined quality indicators for electronic data (AMR and ACU). Cohort exit was defined as the earliest date of the following: first diagnosis of IBD; de-registration with the GP practice contributing data; death; or 31/12/2018.

**Main outcome definitions:**

The main outcomes of interest were newly diagnosed CD, UC or any IBD. The any IBD category included specific and general terms for IBD (comprising CD, UC, IBDU and unspecified IBD). Read code lists, adapted from those used in previous literature,[12, 13, 19] were generated for all three main outcomes using published methodology,[20] then subsequently discussed with a panel of experts including gastroenterologists, epidemiologists, a GP and a statistician (Appendix Read code lists).

As a quality filter, individuals were only included in the study as cases if they had at least two IBD Read codes recorded on separate dates or at least one IBD Read code plus at least one prescription for a drug commonly used to treat IBD (any aminosalicylate or rectal steroid enema listed in chapter 1.5 of the British National Formulary,[21] azathioprine, mercaptopurine, methotrexate, ciclosporin, infliximab or adalimumab) (Appendix Read code lists). As 'incident IBD cases' (who have not been prescribed 'IBD drugs') were required to have their diagnosis verified on a subsequent GP visit, we anticipated that this would result in under ascertainment of those diagnosed in the final months of the study period. Thus, temporal trends in incidence are graphically presented for the period 2000-2017 as opposed to 2000-2018.The date at which the first recording of any IBD code or IBD drug prescription was made was classified as the incident date. For individuals who had been given a code for both UC and CD in their lifetime, the most recent code recorded was used as their final diagnosis.

A separate algorithm was developed to explore the validity of the diagnosis of IBD in IMRD. This involved checking whether individuals who had ever been given a medical Read code for IBD had a record of 1) presentation with

For peer review only - http://bmjopen.bmj.com/site/about/guidelines.xhtml

symptoms suggestive of IBD (abdominal pain, diarrhoea, bloody stools, weight loss); 2) a prescription for a drug commonly used to treat IBD. 'Incident cases' were not required to meet these criteria to be included in the analysis..

**Covariates:**

The following covariates were included in the analyses: 1) birth gender, 2) age by Montreal/Paris classification,[22, 23] (A classification system for IBD whereby the 'A' variable describes 'age at diagnosis', the levels of which are: A1a (0-9 years); A1b (10-17 years); A2 (17-40 years) and A3 (40+ years), 3) calendar time, 4) Townsend deprivation index (a quintile measurement of social deprivation based on post code linked census data),[24] 5) geographical location of GP practice. This was included at the level of former Strategic Health Authority (a defined region responsible for the management of health services for that particular area) for England and at the level of country for Scotland, Wales and Northern Ireland.

**Statistical analyses:**

Crude incidence estimates per 100,000 person-years at risk were calculated by dividing the total number of cases by the total number of person-years of follow up then multiplying by 100,000. This was done separately for CD, UC and any IBD with 95% confidence intervals estimated assuming a Poisson distribution. Stratified incidence rates were calculated by sex, age, Townsend deprivation index and geographical location. Time period was fitted as both a continuous variable and a categorical variable by calendar year. Mixed multivariable Poisson regression was used to estimate incidence rate ratios (IRRs). Individuals with missing data on Townsend score were included in the analysis using 'missing' as a level to the Townsend variable. General practice was included as a random effect to account for any data clustering by practice, the other covariates were included as fixed effects. The Wald test was used to test for significance of categorical variables in the regression model and to test for multiplicative interactions.

Point prevalence was calculated by dividing all cases of IBD (both incident and prevalent) by the total number of individuals contributing data to the cohort on the last day of the study period.

StataCorp. 2017. *Stata Statistical Software: Release 15*. College Station, TX: StataCorp LLC was used for all analyses.

**Sensitivity analysis:**

In the sensitivity analysis we broadened our case definition to include any individual who had a single IBD medical Read code (as opposed to two medical Read codes or one medical Read code plus one relevant prescription).

**Ethics:**

IMRD data collection was approved by the NHS South-East Multicentre Research Ethics Committee in 2003. This study was approved by the Scientific Research Committee (SRC) on 29/09/2018 (SRC reference 18THIN082).

**Patient and public involvement:**

We involved representatives from the University College Hospitals NHS Foundation Trust IBD patient panel in the early stages of protocol design. However, we did not involve PPI representatives in other aspects of study design or analysis due to IMRD licence agreements and the technical computer programming methods that were involved. We intend to involve PPI representatives in writing a plain language summary for dissemination to peers and patient groups.**RESULTS:**

11,325,025 individuals (78,985,977 person-years of follow up) were included in the cohort. 5,541,508 (48.9%) were male. 7,944,975 (70.0%) were registered with a GP practice in England, 1,690,503 (14.9%) Scotland, 1,285,722 (11.4%) Wales and 403,825 (3.6%) Northern Ireland. Mean (SD) age at cohort entry was 34 (22.8) years and median (IQR) follow up was 5.4 (2.0-11.6) years. we identified 65,700 cases of IBD, including 24,991 cases of CD and 36,705 cases of UC. Among these, 22,560 (8,077 for CD and 12,369 for UC) were incident diagnoses made during study follow up. Overall crude incidence estimates were 28.6 (95% CI 28.2-28.9), 10.2 (95% CI 10.0-10.5) and 15.7 (95% CI 15.4-15.9)/100,000 person-years for 'any IBD', CD and UC respectively. Point prevalence estimates on 31/12/2018 were 725, 276 and 397 per 100,000 people for 'any IBD', CD and UC respectively.

24,173/28,879 (83.7%) individuals given a new code for IBD since entering the study had a record of a prescription for a drug commonly used to treat IBD, comparing to 1.8% for the whole cohort. Additionally, 23,337/28,879 (80.8%) with a code for IBD had a record of presentation to their GP with either diarrhoea, bloody stools, abdominal pain or weight loss, comparing to 33.6% for the whole cohort.

**Table.1 Incidence rates and adjusted IRRs of CD and UC by sex, age, calendar year, geographical location and social deprivation**

For peer review only

| | Incidence of CD | | Incidence of UC | |
|---|---|---|---|---|
| | Rate per 100,000 person-years (95% CI) | Adjusted IRR (95% CI)* | Rate per 100,000 person-years (95% CI) | Adjusted IRR (95% CI)* |
| **Overall** | 10.2 (10.0 to 10.5) | | 15.7 (15.4 to 15.9) | |
| **Sex** | | | | |
| Male | 9.3 (0.00 ( to 9.6) | 1 | 16.7 (16.3 to 17.1) | 1 |
| Female | 11.1 (10.8 to 11.5) | 1.20 (1.15 to 1.25) | 14.7 (14.3 to 15.0) | 0.87 (0.84 to 0.90) |
| **Age , years** | | | | |
| 0-9 | 1.6 (1.3 to 1.9) | 0.11 (0.09 to 0.13) | 1.0 (0.8 to 1.3) | 0.05 (0.04 to 0.07) |
| 10-16 | 11.5 (10.7 to 12.4) | 0.77 (0.71 to 0.84) | 7.4 (6.8 to 8.1) | 0.38 (0.35 to 0.42) |
| 17-40 | 14.9 (14.4 to 15.4) | 1 | 19.2 (18.7 to 19.8) | 1 |
| 40+ | 9.2 (8.9 to 9.5) | 0.62 (0.59 to 0.64) | 17.9 (17.5 to 18.3) | 0.92 (0.89 to 0.96) |
| **Year (linear change)** | | 0.99 (0.98 to 0.99) | | 0.98 (0.98-0.99) |
| **Year (categorical variable)** | | | | |
| 2000 | 10.7 (9.5 to 12.1) | 1 | 18.6 (17.0 to 20.4) | 1 |
| 2001 | 10.3 (9.2 to 11.6) | 0.97 (0.82 to 1.14) | 17.9 (16.4 to 19.5) | 0.96 (0.85 to 1.09) |
| 2002 | 10.8 (9.7 to 11.9) | 1.00 (0.85 to 1.17) | 16.7 (15.4 to 18.1) | 0.89 (0.79 to 1.01) |
| 2003 | 10.3 (9.3 to 11.4) | 0.95 (0.82 to 1.11) | 17.0 (15.8 to 18.4) | 0.91 (0.81 to 1.03) |
| 2004 | 10.2 (9.3 to 11.2) | 0.94 (0.80 to 1.09) | 17.3 (16.1 to 18.6) | 0.93 (0.82 to 1.04) |
| 2005 | 11.6 (10.6 to 12.6) | 1.07 (0.92 to 1.24) | 16.1 (14.9 to 17.3) | 0.86 (0.77 to 0.97) |
| 2006 | 11.7 (10.7 to 12.7) | 1.07 (0.93 to 1.24) | 16.3 (15.1 to 17.5) | 0.87 (0.78 to 0.98) |
| 2007 | 10.5 (9.6 to 11.4) | 0.96 (0.83 to 1.12) | 16.7 (15.5 to 17.9) | 0.89 (0.80 to 1.00) |
| 2008 | 10.6 (9.7 to 11.5) | 0.97 (0.84 to 1.13) | 17.8 (16.6 to 19.0) | 0.95 (0.85 to 1.07) |
| 2009 | 10.8 (9.9 to 11.7) | 0.99 (0.86 to 1.15) | 16.4 (15.3 to 17.6) | 0.88 (0.79 to 0.99) |
| 2010 | 10.3 (9.4 to 11.2) | 0.95 (0.82 to 1.10) | 15.6 (14.5 to 16.8) | 0.84 (0.75 to 0.94) |
| 2011 | 10.6 (9.7 to 11.5) | 0.97 (0.84 to 1.13) | 14.9 (13.8 to 16.0) | 0.80 (0.71 to 0.90) |
| 2012 | 11.1 (10.2 to 12.1) | 1.02 (0.88 to 1.18) | 15.2 (14.2 to 16.3) | 0.82 (0.73 to 0.92) |
| 2013 | 9.1 (8.3 to 10.0) | 0.84 (0.72 to 0.97) | 15.1 (14.0 to 16.2) | 0.82 (0.73 to 0.92) |
| 2014 | 10.5 (9.6 to 11.5) | 0.96 (0.82 to 1.11) | 14.5 (13.5 to 15.7) | 0.79 (0.70 to 0.89) |
| 2015 | 9.3 (8.4 to 10.3) | 0.84 (0.72 to 0.98) | 14.2 (13.0 to 15.4) | 0.77 (0.68 to 0.87) |
| 2016 | 9.0 (8.1 to 10.1) | 0.80 (0.68 to 0.95) | 13.7 (12.5 to 15.0) | 0.74 (0.65 to 0.84) |
| 2017 | 9.0 (8.0 to 10.1) | 0.79 (0.67 to 0.94) | 12.6 (11.4 to 13.9) | 0.68 (0.60 to 0.78) |
| 2018 | 5.9 (5.1 to 6.9) | 0.52 (0.43 to 0.63) | 10.0 (8.9 to 11.2) | 0.54 (0.47 to 0.63) |
| **Region** | | | | |
| East Midlands | 9.6 (8.2 to 11.2) | 1 | 17.5 (15.6 to 19.6) | 1 |
| East of England | 10.9 (10.0 to 11.9) | 1.18 (0.96 to 1.44) | 17.4 (16.2 to 18.6) | 1.01 (0.86 to 1.19) |
| London | 8.9 (8.3 to 9.6) | 0.94 (0.77 to 1.14) | 15.3 (14.5 to 16.2) | 0.94 (0.81 to 1.09) |
| North East | 9.8 (8.2 to 11.6) | 1.03 (0.80 to 1.34) | 18.1 (16.0 to 20.5) | 1.08 (0.88 to 1.32) |
| North West | 11.9 (11.1 to 12.7) | 1.27 (1.05 to 1.54) | 15.8 (14.9 to 16.7) | 0.93 (0.80 to 1.08) |
| Northern Ireland | 13.1 (12.0 to 14.4) | 1.43 (1.16 to 1.75) | 16.2 (14.9 to 17.5) | 1.02 (0.87 to 1.21) |
| Scotland | 12.3 (11.7 to 12.9) | 1.36 (1.13 to 1.64) | 15.3 (14.7 to 16.0) | 0.97 (0.84 to 1.13) |
| South Central | 9.5 (8.9 to 10.2) | 1.01 (0.83 to 1.23) | 16.1 (15.2 to 17.0) | 0.92 (0.79 to 1.07) |
| South East Coast | 9.4 (8.7 to 10.1) | 1.04 (0.85 to 1.26) | 15.7 (14.9 to 16.6) | 0.95 (0.81 to 1.10) |
| South West | 10.1 (9.3 to 11.0) | 1.08 (0.89 to 1.32) | 15.2 (14.2 to 16.2) | 0.88 (0.75 to 1.03) |
| Wales | 8.9 (8.4 to 9.6) | 0.98 (0.81 to 1.19) | 14.1 (13.4 to 14.9) | 0.87 (0.75 to 1.01) |
| West Midlands | 8.4 (7.7 to 9.1) | 0.89 (0.73 to 1.09) | 16.2 (15.3 to 17.2) | 0.96 (0.82 to 1.12) |
| Yorkshire & | 9.6 (8.2 to 11.2) | 1.00 (0.78 to 1.28) | 15.8 (14.0 to 17.8) | 0.91 (0.74 to 1.10) |

| | | | | |
|---|---|---|---|---|
| Humber | | | | |
| **Townsend, quintile** | | | | |
| Missing | 10.1 (9.6 to 10.7) | 1.08 (0.99 to 1.17) | 15.1 (14.4 to 15.7) | 0.95 (0.89 to 1.02) |
| 1 | 9.6 (9.1 to 10.1) | 1 | 16.8 (16.2 to 17.5) | 1 |
| 2 | 10.0 (9.5 to 10.5) | 1.02 (0.95 to 1.10) | 16.5 (15.9 to 17.2) | 0.97 (0.92 to 1.03) |
| 3 | 10.7 (10.1 to 11.2) | 1.07 (1.00 to 1.15) | 16.2 (15.5 to 16.8) | 0.96 (0.91 to 1.01) |
| 4 | 10.4 (9.8 to 11.0) | 1.03 (0.96 to 1.12) | 14.8 (14.1 to 15.5) | 0.88 (0.83 to 0.94) |
| 5 | 11.2 (10.5 to 11.9) | 1.08 (0.99 to 1.17) | 13.3 (12.6 to 14.1) | 0.80 (0.74 to 0.86) |

*Adjusted for other variables considered; Sex, ageband, year, region, Townsend quintile, respectively
*IRRs compared to the reference group for each categorical variable

*Fig.1 Crude incidence estimates for any IBD, stratified by Montreal/Paris age classification and calendar year, over the period 2000-2017.*

For the period 2000-2017, incidence of 'any IBD' remained relatively stable for those aged 17-40 years (A2 disease) and those aged 0-9 years (A1a disease). However, for those aged over 40 years (A3 disease), crude incidence fell from 37.8 (95% CI 34.5-41.4) to 23.6 (21.3-26.0) at an average rate of 2.3% (95% CI 1.9-2.7) per calendar year ($p<0.0001$) and for those aged 10-16 years (A1b disease), incidence rose from 13.1 (95% CI 8.3-19.5) to 25.4 (95% CI 19.5-32.4) at an average rate of 3.0% (95% CI 1.7-4.3) per calendar year ($p<0.0001$) (Fig.1). When adding an age-time interaction term to the model, we found an interaction for all three main outcomes ($p<0.00001$, $p=0.0046$, $p<0.00001$ for 'any IBD', CD and UC respectively). Ageband specific age-time interaction coefficients confirmed increasing incidence in adolescents ages 10-16, decreasing incidence in those aged 40+ years and stable incidence in age groups 0-9 and 17-40 years (Appendix table 1)

*Fig.2 Crude incidence estimates of IBD, stratified by calendar year, over the period 2000-2017.*

**CD incidence:**
During the study period, CD incidence fell slightly from 10.7 (95% CI 9.5-12.1) to 9.0 (95% CI 8.0-10.1)/100,000 person-years at an average rate of 1.0% (95% CI 0.6-1.5) per calendar year ($p<0.0001$) (Fig. 2). However, in children <17 years, incidence rose from 3.9 (95% CI 2.2-6.2) to 6.9 (95% CI 4.9-9.3)/100,000 person-years at an average rate of 2.9% (95% CI 1.3-4.4) per calendar year ($p<0.0001$) (Fig .3). Although overall crude incidence was higher for boys than for girls (7.4 (95% CI 6.8-8.0) vs 4.1 (95% CI 3.6-4.6)), a significant rise in incidence was observed for both sexes (average 2.7% (95% CI 0.8-4.6) and 3.3% (95% CI -0.6-6.0) rise per calendar year for boys and girls respectively). No change in incidence was observed for children aged 0-9 years. However, for adolescents aged 10-16 years, incidence rose from 7.6 (95% CI 4.2-12.8) to 13.1 (95% CI 9.0-18.4)/100,000 person-years at an average rate of 2.8% (1.2-4.5) per calendar year ($p=0.001$).

Incidence of CD was highest in Northern Ireland, Scotland and the North West (13.1 (95% CI 12.0-14.4), 12.3 (95% CI 11.7-12.9) and 11.9 (95% CI 11.1-12.7)/100,000 person-years respectively) and lowest in Wales, London and the West Midlands (8.9 (95% CI 8.4-9.6), 8.9 (95% CI 8.3-9.6) and 8.4(95% CI 7.7-9.1)/100,000 person-years respectively (Fig.4). We observed no association between social deprivation and incidence of CD after adjusting for sex, calendar year, age and geographical location (Table.1).

**UC incidence:**
Incidence of UC dropped to a greater extent than for CD over the study period; from 18.6 (95% CI 17.0-20.4) to 12.6 (95% CI 11.4-13.9)/100,000 person-years at an average rate of 1.6% (95% CI 1.3-2.0%) per calendar year ($p<0.0001$) (Fig.2). The fall in incidence was most pronounced for those aged over 40 years, in whom a 45% drop in incidence was observed, falling from 24.1 (95% CI 21.5-26.9) to 13.3 (95% CI 11.6-15.2)/100,000 person-years (average 3.1% (95% CI 2.6-3.6) decrease per calendar year ($p<0.0001$))

For peer review only

In children aged <17, incidence rose from 2.0 (95% CI 0.9-3.9) to 5.0 (95% CI 3.4-7.2)/100,000 person-years (average 2.5% (95% CI 0.5-4.4) rise per calendar year (p=0.01) (Fig.3). The rise in incidence was largely driven by adolescent boys aged 10-16 in whom incidence rose by 3.4% (95% CI 0.8-6.2) per calendar year (p=0.01). No significant change in incidence was observed in girls aged 10-16 years or children of either sex aged 0-9 years.

Incidence of UC was highest in the North East, the East of England and the East Midlands (18.1 (95% CI 16.0-20.5), 17.4 (95% CI 16.2-18.6) and 17.5 (95% CI 15.6-19.6)/100,000 person-years respectively) and lowest in Wales, the South West and London (14.1 (95% CI 13.4-14.9), 15.2 (95% CI 14.2-16.2) and 15.3 (95% CI 14.5-16.2)/100,000 person-years respectively) (Fig.4). We observed higher incidence of UC in individuals from the least deprived quintile compared to most deprived (16.8 (95% CI 16.2-17.5) vs 13.3 (95% CI 12.6-14.1)/100,000 person-years, adjusted IRR 0.80 (95% CI 0.74-0.86) (Table.1).

*Fig.3 Crude incidence estimates of IBD in children <17 years, stratified by calendar year, over the period 2000-2017.*

*Fig.4 Map showing overall crude incidence of CD and UC stratified by geographical region.*

**Sensitivity analysis:**
When broadening the case definition to include any individual who had a single IBD medical Read code, we observed overall incidence rates of 36.6 (95% CI 36.2-37.0), 12.9 (95% CI 12.7-13.2) and 19.3 (95% CI 19.0-19.6)/100,000 person-years for 'any IBD', CD and UC respectively. We observed a similar fall in incidence of UC, decreasing from 21.8 (95% CI 20.0-23.8) to 17.9 (95% 16.4-19.4)/100,000 person-years (average decrease 1.3% (95% CI 0.9-1.6) per calendar year). However, no fall in CD incidence was observed (Appendix Fig.1). When stratifying IBD incidence by five year age bands, the peak in incidence later in life was higher and occurred later than in the primary analysis (Appendix Fig.2/Appendix Fig.3).

For peer review only

**DISCUSSION:**

This is one of the largest observational studies undertaken to investigate trends in IBD epidemiology. Although incidence of IBD remained relatively stable for those aged 17-40 years and those aged 0-9 years, we observed a 38% fall in incidence for those aged over 40 years and a 94% rise in incidence in the adolescent population. The most recent incidence estimates are in line with some of the highest reported rates of paediatric IBD internationally.[25-27]

Study strengths include the large sample size and the prospective collection of health care records representative of 'real-life' clinical practice. Unlike previous incidence/prevalence studies that have relied on external data sources to estimate denominator population characteristics, we were able to extract demographics and person-time follow up for all individuals in our cohort, including those who did not develop IBD. Additionally, IMRD has been shown to be broadly representative of the UK in terms of age, sex, mortality rates and prevalence of numerous comorbidities,[15] allowing us to draw inferences from our data and relate this to the UK population as a whole. Not only has the diagnosis of IBD been validated in a similar GP database,[12] but we have demonstrated that the majority of individuals coded for IBD in IMRD have been prescribed drugs commonly used to treat IBD and presented with symptoms in keeping with IBD. This would support the argument that IMRD represents an important and useful resource for further epidemiological studies of IBD.

Limitations arise when conducting GP database research, particularly as the primary use of the software that contributes to IMRD is for patient management purposes rather than medical research. Thus, data can be incomplete and will often only reflect those events that are deemed to be relevant to the patient's care. Given that we were also reasonably strict with our case definition this may have resulted in underascertainment of cases. Although we find reason to be confident in the validity of the data, we weren't able to confirm our cases by evidence of radiological, endoscopic or histological findings. Therefore, it is possible that some individuals were misclassified. There was a small risk of duplication of medical records. This could occur if a patient de-registered with one practice contributing to IMRD then subsequently registered with another IMRD practice during the observation period. This is likely to be the case for a very small number of individuals as IMRD only covered 5-6% of UK GP practices during the study period. Although the total number of individuals contributing may be a slight overestimate, this would have no effect on incidence or prevalence rates. This is for two reasons; 1) Duplicated records would cover different time periods during the study without overlap 2) We took steps to ensure that prevalent cases of IBD newly transferring to practices were not counted as incident cases [18] Therefore, incident cases were not counted twice.

Data from a multi-centre European study (including two UK sites in North West London and East Yorkshire) reported site incidence rates of 2.6 and 8.4 /100,000 person-years for CD and 15.9 and 8.2 /100,000 years for UC.[28] However, only a small number of UK cases were included (n=167). Incidence rates of 8.3 (3.4-13.2) and 13.9 (95% CI 7.5-20.3)/100,000 person-years for CD and UC respectively have been reported in North-East England for the period 1990-1994.[13] We report overall incidence rates of 10.2 (95% CI 10.0-10.5) and 15.6 (95% CI 15.3-15.9)/100,000 person-years for CD and UC respectively in a far larger cohort and at a national level. We report considerable geographical variation in IBD incidence across the UK with notably high CD incidence in Scotland and Northern Ireland and high UC incidence in the East of England. This may reflect variation in lifestyle factors such as dietary habits and importantly smoking (it is estimated that 14.4% adults in England smoke compared to 15.9% in Scotland and 16.3% in Northern Ireland [29].

A Danish study based on nationwide registry data (1995-2012) observed comparable incidence rates: 8.9 (95% CI 8.3-9.5) and 10.3 (95% CI 9.7-11.0)/100,000 person-years for CD and 23.4 (95% CI 22.4-24.5) and 23.2 (95% CI 22.2-24.3)/100,000 person-years for UC in males and females, respectively.[30] In contrast to our results, they observed overall rising incidence rates of IBD, but their study was conducted in a different country over an earlier time period including the 1990s when a rise in IBD incidence was described in many high-income countries. Although they adjusted for age in their analysis, temporal trends in incidence stratified by age group were not reported.

In the sensitivity analysis we observed higher than expected overall incidence rates. Additionally, for UC, the observed peak in incidence for older individuals was higher than the peak in incidence for younger individuals (Appendix Fig.2); this would be unusual in clinical practice. An explanation for this could be that a number of these patients, who perhaps had colitis of a different aetiology, had been misclassified as IBD. On the basis of these findings, one Read code alone was deemed not specific enough for the diagnosis of IBD.

For peer review only

In keeping with published literature we observed a rising incidence of paediatric IBD during the early 21st century[31] and we provide further evidence of male preponderance in paediatric IBD when compared to adult onset disease.[32, 33] Uniquely, in our study we have demonstrated rising incidence of adolescent IBD in the context of stable incidence in those aged 17-40 years and falling incidence in the over 40s. This may represent a general shift towards earlier diagnosis of IBD for all age groups except the very young (age 0-9 years). Given that IBD most commonly presents in the second to fourth decade of life [1, 2]rising incidence in adolescents might be explained by a number of factors, including improvements in referral pathways and the introduction of new diagnostic tools (e.g. faecal calprotectin testing or capsule endoscopy) resulting in cases being picked up earlier. However, one would expect stepwise increases in incidence when new diagnostic tools are rolled out, which we did not observe. Moreover, if rising incidence of adolescent IBD is due to improved referral pathways, a corresponding rise in incidence might be expected in very young children as well as adolescents. It could be argued that changes in GP coding practice may be contributing. But again, one would expect comparable changes in younger age groups if this were the case. On the other hand, if the epidemiological patterns we observed reflect real increases in the incidence of pathology this is of great concern and could represent earlier manifestation of disease related to environmental exposures in childhood and adolescence.

Our prevalence estimates were very similar to those reported in a well-validated IBD cohort in Lothian, Scotland;[10] our estimate of IBD prevalence for Scotland on 31/08/2018 was 810 per 100,000 compared to 832 per 100,000 reported by their group. Although our study lacked linkage to secondary care records, the similar prevalence estimates would support the argument that few cases were missed. In 2018, 67,150,000 people were estimated to be living in the UK from which we might extrapolate from our data that there were approximately 487,000 people living with IBD in the UK at that time.

Compounding prevalence of IBD has been demonstrated in Canada and in Scotland.[10, 34] This relates to the principle that although IBD incidence may be static or falling, whilst IBD mortality remains very low, overall prevalence will increase (more people are being diagnosed than are dying); In Scotland IBD prevalence is estimated to reach 1.0% by 2028. We report rising incidence rates of IBD in younger populations and falling incidence in older age groups. Thus, not only will services need to be attuned to rising IBD prevalence and an ageing demographic, but also to increasing numbers of new diagnoses in young people who will require lifelong care. This is in the context of significant financial challenges to health services.

**CONCLUSION:**

Although we observed a stable or falling incidence of IBD in adults over a 18 year period, our results are consistent with some of the highest reported global incidence and prevalence rates for IBD, with a 94% rise in incidence in adolescents. These findings are concerning and suggest detailed prospective studies are required to understand the aetiological drivers.

For peer review only

**REFERENCES:**

1. Ungaro R, Mehandru S, Allen PB, et al. Ulcerative colitis. *Lancet (London, England)*. 2017;389(10080):1756-70.
2. Kalla R, Ventham NT, Satsangi J, et al. Crohn's disease. *BMJ (Clinical research ed)*. 2014;349:g6670.
3. Burisch J, Jess T, Martinato M, et al. The burden of inflammatory bowel disease in Europe. *Journal of Crohn's & colitis*. 2013;7(4):322-37.
4. Ng SC, Shi HY, Hamidi N, et al. Worldwide incidence and prevalence of inflammatory bowel disease in the 21st century: a systematic review of population-based studies. *Lancet (London, England)*. 2018;390(10114):2769-78.
5. Whelan G. Epidemiology of inflammatory bowel disease. *The Medical clinics of North America*. 1990;74(1):1-12.
6. Ashton JJ, Wiskin AE, Ennis S, et al. Rising incidence of paediatric inflammatory bowel disease (PIBD) in Wessex, Southern England. *Archives of disease in childhood*. 2014;99(7):659-64.
7. Steed H, Walsh S, Reynolds N. Crohn's disease incidence in NHS Tayside. *Scottish medical journal*. 2010;55(3):22-5.
8. Stone MA, Mayberry JF, Baker R. Prevalence and management of inflammatory bowel disease: a cross-sectional study from central England. *European journal of gastroenterology & hepatology*. 2003;15(12):1275-80.
9. Hassan K, Cowan FJ, Jenkins HR. The incidence of childhood inflammatory bowel disease in Wales. *European journal of pediatrics*. 2000;159(4):261-3.
10. Jones G-R, Lyons M, Plevris N, et al. IBD prevalence in Lothian, Scotland, derived by capture–recapture methodology. *Gut*. 2019:gutjnl-2019-318936.
11. digital N. Patients Registered at a GP Practice September 2019 2019 [Available from: https://digital.nhs.uk/data-and-information/publications/statistical/patients-registered-at-a-gp-practice/september-2019.
12. Lewis JD, Brensinger C, Bilker WB, et al. Validity and completeness of the General Practice Research Database for studies of inflammatory bowel disease. *Pharmacoepidemiology and drug safety*. 2002;11(3):211-8.
13. Rubin GP, Hungin AP, Kelly PJ, et al. Inflammatory bowel disease: epidemiology and management in an English general practice population. *Alimentary pharmacology & therapeutics*. 2000;14(12):1553-9.
14. Benson T. The history of the Read Codes: the inaugural James Read Memorial Lecture 2011. *Informatics in primary care*. 2011;19(3):173-82.
15. Blak BT, Thompson M, Dattani H, et al. Generalisability of The Health Improvement Network (THIN) database: demographics, chronic disease prevalence and mortality rates. *Informatics in primary care*. 2011;19(4):251-5.
16. Horsfall L, Walters K, Petersen I. Identifying periods of acceptable computer usage in primary care research databases. *Pharmacoepidemiology and drug safety*. 2013;22(1):64-9.
17. Maguire A, Blak BT, Thompson M. The importance of defining periods of complete mortality reporting for research using automated data from primary care. *Pharmacoepidemiology and drug safety*. 2009;18(1):76-83.
18. Lewis JD, Bilker WB, Weinstein RB, et al. The relationship between time since registration and measured incidence rates in the General Practice Research Database. *Pharmacoepidemiology and drug safety*. 2005;14(7):443-51.
19. Abrahami D, Douros A, Yin H, et al. Dipeptidyl peptidase-4 inhibitors and incidence of inflammatory bowel disease among patients with type 2 diabetes: population based cohort study. *BMJ (Clinical research ed)*. 2018;360:k872.
20. Dave S, Petersen I. Creating medical and drug code lists to identify cases in primary care databases. *Pharmacoepidemiology and drug safety*. 2009;18(8):704-7.
21. Committee JF. British National Formulary. 76 ed. London: Pharmaceutical Press; 2018.
22. Satsangi J, Silverberg MS, Vermeire S, et al. The Montreal classification of inflammatory bowel disease: controversies, consensus, and implications. *Gut*. 2006;55(6):749-53.
23. Levine A, Griffiths A, Markowitz J, et al. Pediatric modification of the Montreal classification for inflammatory bowel disease: the Paris classification. *Inflammatory bowel diseases*. 2011;17(6):1314-21.

For peer review only

24. Townsend P PPBA. Health and deprivation. Inequality and the North. London: Croom Helm. *Health Policy*. 1988;10(2):207-6.
25. Malmborg P, Grahnquist L, Lindholm J, et al. Increasing incidence of paediatric inflammatory bowel disease in northern Stockholm County, 2002-2007. *Journal of pediatric gastroenterology and nutrition*. 2013;57(1):29-34.
26. Benchimol EI, Guttmann A, Griffiths AM, et al. Increasing incidence of paediatric inflammatory bowel disease in Ontario, Canada: evidence from health administrative data. *Gut*. 2009;58(11):1490-7.
27. Perminow G, Brackmann S, Lyckander LG, et al. A characterization in childhood inflammatory bowel disease, a new population-based inception cohort from South-Eastern Norway, 2005-07, showing increased incidence in Crohn's disease. *Scandinavian journal of gastroenterology*. 2009;44(4):446-56.
28. Burisch J, Pedersen N, Cukovic-Cavka S, et al. East-West gradient in the incidence of inflammatory bowel disease in Europe: the ECCO-EpiCom inception cohort. *Gut*. 2014;63(4):588-97.
29. Statistics OfN. Adult smoking habits in the UK 2018 [Available from: https://www.ons.gov.uk/peoplepopulationandcommunity/healthandsocialcare/healthandlifeexpectancies/bulletins/adultsmokinghabitsingreatbritain/2018.
30. Norgard BM, Nielsen J, Fonager K, et al. The incidence of ulcerative colitis (1995-2011) and Crohn's disease (1995-2012) - based on nationwide Danish registry data. *Journal of Crohn's & colitis*. 2014;8(10):1274-80.
31. Benchimol EI, Fortinsky KJ, Gozdyra P, et al. Epidemiology of pediatric inflammatory bowel disease: a systematic review of international trends. *Inflammatory bowel diseases*. 2011;17(1):423-39.
32. Ashton JJ, Cullen M, Afzal NA, et al. Is the incidence of paediatric inflammatory bowel disease still increasing? *Archives of disease in childhood*. 2018;103(11):1093-4.
33. Henderson P, Hansen R, Cameron FL, et al. Rising incidence of pediatric inflammatory bowel disease in Scotland. *Inflammatory bowel diseases*. 2012;18(6):999-1005.
34. Kaplan GG, Bernstein CN, Coward S, et al. The Impact of Inflammatory Bowel Disease in Canada 2018: Epidemiology. *Journal of the Canadian Association of Gastroenterology*. 2019;2(Suppl 1):S6-s16.

For peer review only

**FUNDING:**

This work was supported by The Charles Wolfson Charitable Trust [grant number 539234] and The Harbour Foundation [grant number 549321]. Laura Horsfall is supported by a Wellcome Trust Fellowship [grant number 209207/Z/17/Z]. No funder had any role in study design; in the collection, analysis, and interpretation of data; in the writing of the report; and in the decision to submit the article for publication. The lead author confirms the independence of researchers from funders and that all authors, external and internal, had full access to all of the data (including statistical reports and tables) in the study and can take responsibility for the integrity of the data and the accuracy of the data analysis.

**TRANSPARENCY STATEMENT:**

The lead author affirms that this manuscript is an honest, accurate, and transparent account of the study being reported; that no important aspects of the study have been omitted; and that any discrepancies from the study as originally planned have been explained.

**COMPETING INTERESTS DECLARATION:**

All authors have completed the ICMJE uniform disclosure form at www.icmje.org/coi_disclosure.pdf and declare: TJP and AWS report research grants from The Charles Wolfson Charitable Trust and The Harbour Foundation for the submitted work; no financial relationships with any organisations that might have an interest in the submitted work in the previous three years; no other relationships or activities that could appear to have influenced the submitted work.

**CONTRIBUTOR AND GUARANTOR INFORMATION:**

**T J Pasvol** - study concept and design; analysis and interpretation of data; drafting of the manuscript; statistical analysis; obtained funding.

**L Horsfall** - study concept and design; analysis and interpretation of data; critical revision of the manuscript for important intellectual content; statistical analysis.

**S Bloom** - study concept and design; critical revision of the manuscript for important intellectual content.

**A W Segal** - study concept and design; critical revision of the manuscript for important intellectual content; obtained funding.

**C A Sabin** - study concept and design; critical revision of the manuscript for important intellectual content; statistical analysis.

**N Field** - study concept and design; critical revision of the manuscript for important intellectual content.

**G Rait** - study concept and design; analysis and interpretation of data; critical revision of the manuscript for important intellectual content; study supervision.

The corresponding author attests that all listed authors meet authorship criteria and that no others meeting the criteria have been omitted.

**COPYRIGHT/LICENSE FOR PUBLICATION:**

The Corresponding Author has the right to grant on behalf of all authors and does grant on behalf of all authors, a worldwide licence to the Publishers and its licensees in perpetuity, in all forms, formats and media (whether known now or created in the future), to i) publish, reproduce, distribute, display and store the Contribution, ii) translate the Contribution into other languages, create adaptations, reprints, include within collections and create summaries, extracts and/or, abstracts of the Contribution, iii) create any other derivative work(s) based on the Contribution, iv) to exploit all subsidiary rights in the Contribution, v) the inclusion of electronic links from the Contribution to third party material where-ever it may be located; and, vi) licence any third party to do any or all of the above.

**DATA SHARING:**

Due to licence agreements with IQVIA, we are unable to share patient level data from the IQVIA Medical Research Database. However, we are happy to share our data extractions upon reasonable request. Data requesters should email the corresponding author to request the relevant data.

14

429

For peer review only

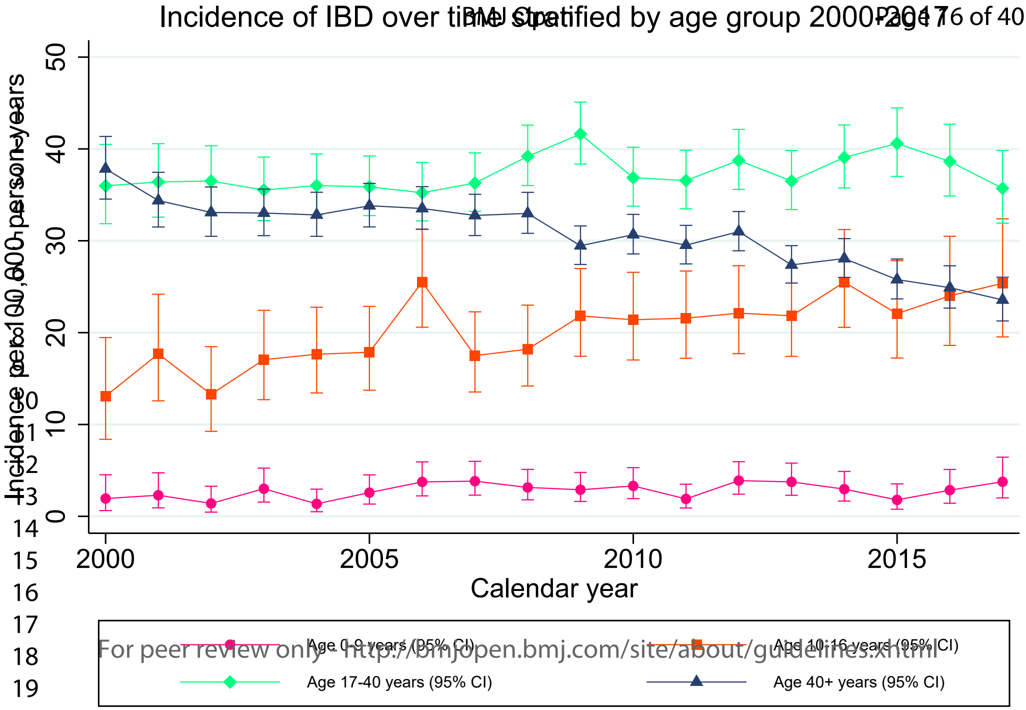


For peer review only - http://bmjopen.bmj.com/site/about/guidelines.xhtml

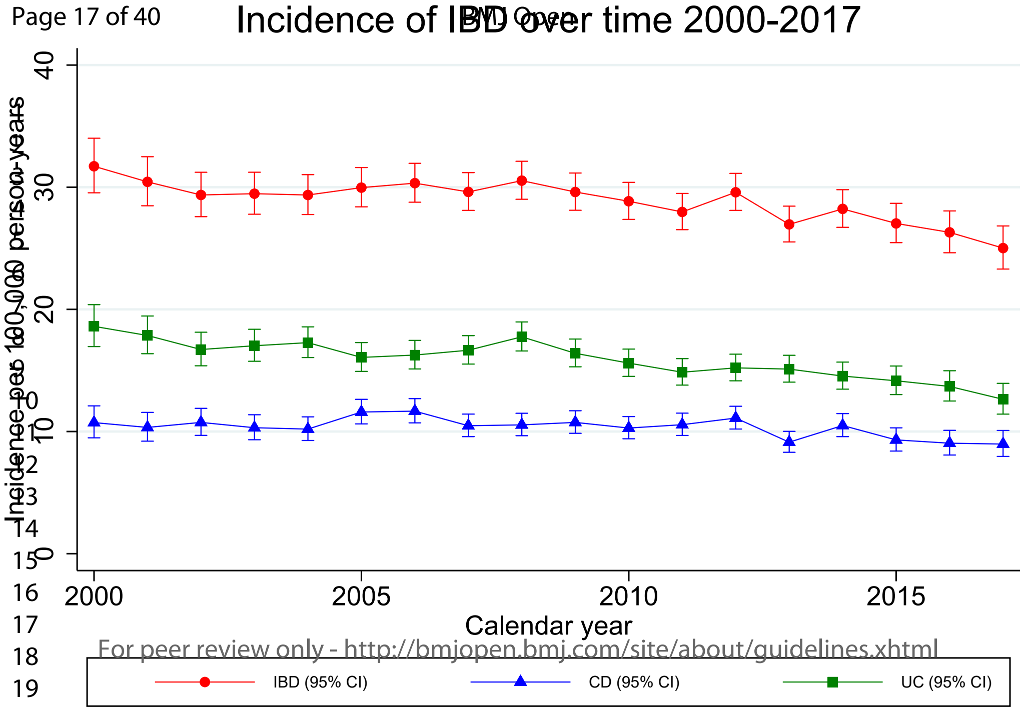


For peer review only - http://bmjopen.bmj.com/site/about/guidelines.xhtml

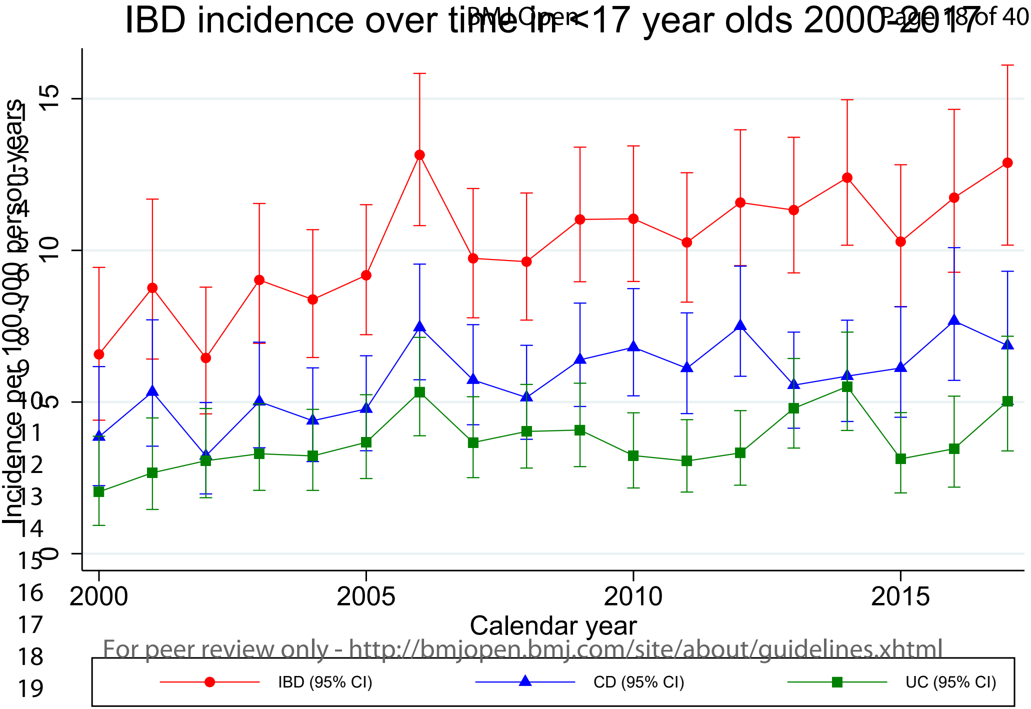


For peer review only - http://bmjopen.bmj.com/site/about/guidelines.xhtml

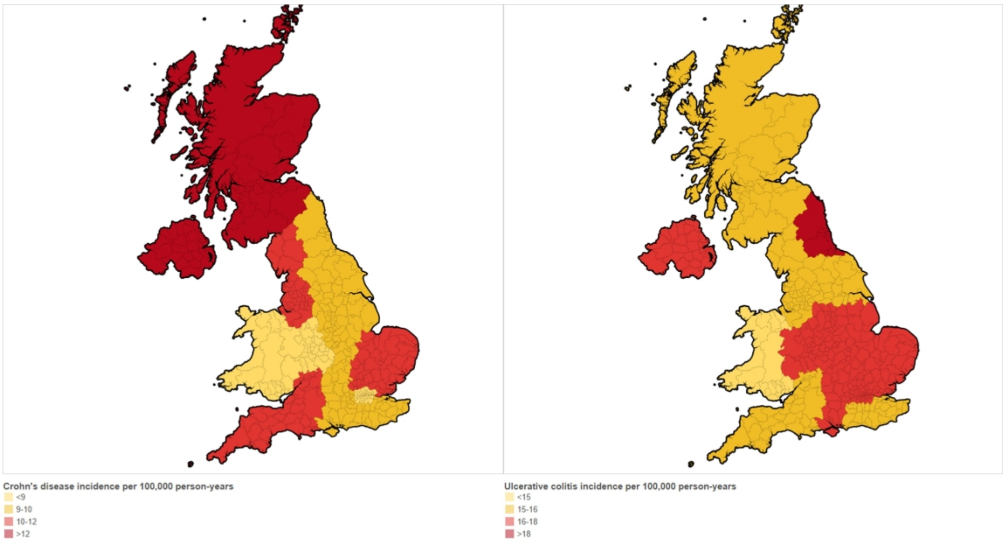


Fig.4 Map showing overall crude incidence of CD and UC stratified by geographical region

338x190mm (300 x 300 DPI)

**APPENDIX:**

| *Appendix table.1 Interaction coefficients (adjusted IRR*) for age-time interactions* | | | |
|---|---|---|---|
| ageband (years) | Any IBD (95% CI) | CD (95% CI) | UC (95% CI) |
| 0-9 | 1.019 (0.993-1.048) | 1.032 (0.996-1.070) | 1.017 (0.973-1.063) |
| 10-16 | 1.027 (1.015-1.039) | 1.025 (1.009-1.041) | 1.024 (1.004-1.044) |
| 17-40 | 1.003 (0.999-1.008) | 0.992 (0.984-0.999) | 1.005 (0.998-1.012) |
| 40+ | 0.978 (0.974-0.982) | 0.980 (0.972-0.987) | 0.970 (0.965-0.976) |
| *Adjusted for other variables considered; Sex, ageband, year, region, Townsend quintile, respectively | | | |

*Appendix fig.1 Crude incidence estimates of IBD, stratified by calendar year, over the period 2000-2017 (sensitivity analysis)*

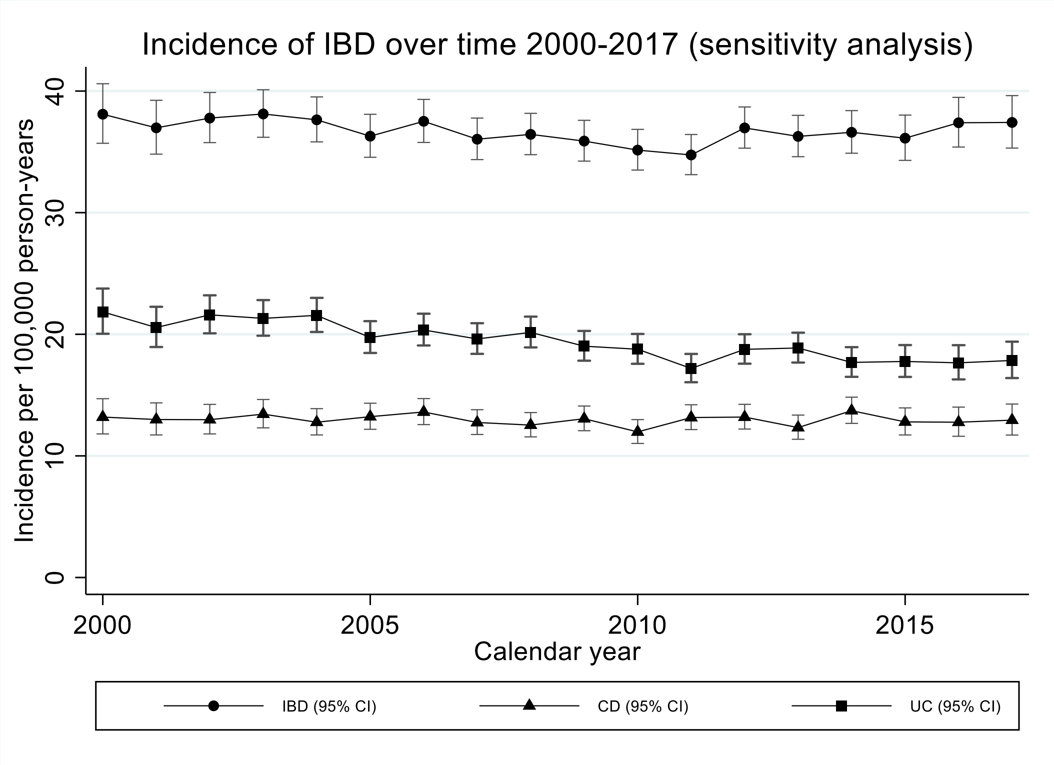


For peer review

*Appendix fig.2 Crude incidence estimates of IBD by five year age bands (primary analysis)*

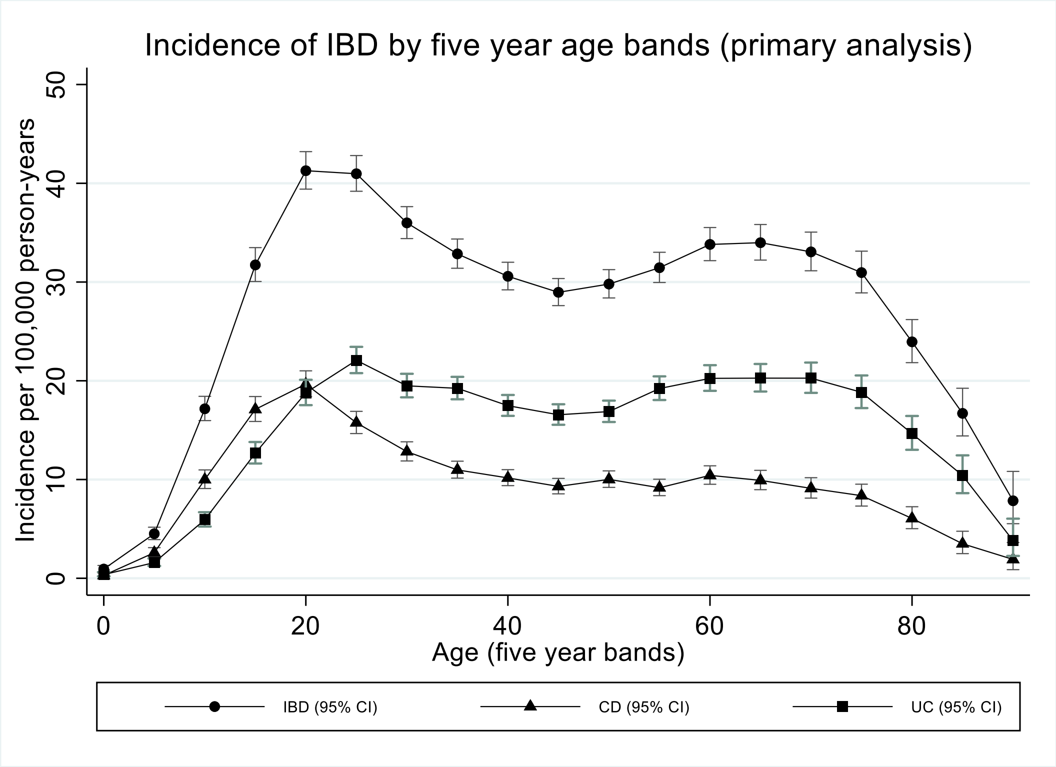


*Appendix fig.3 Crude incidence estimates of IBD by five year age bands (sensitivity analysis)*

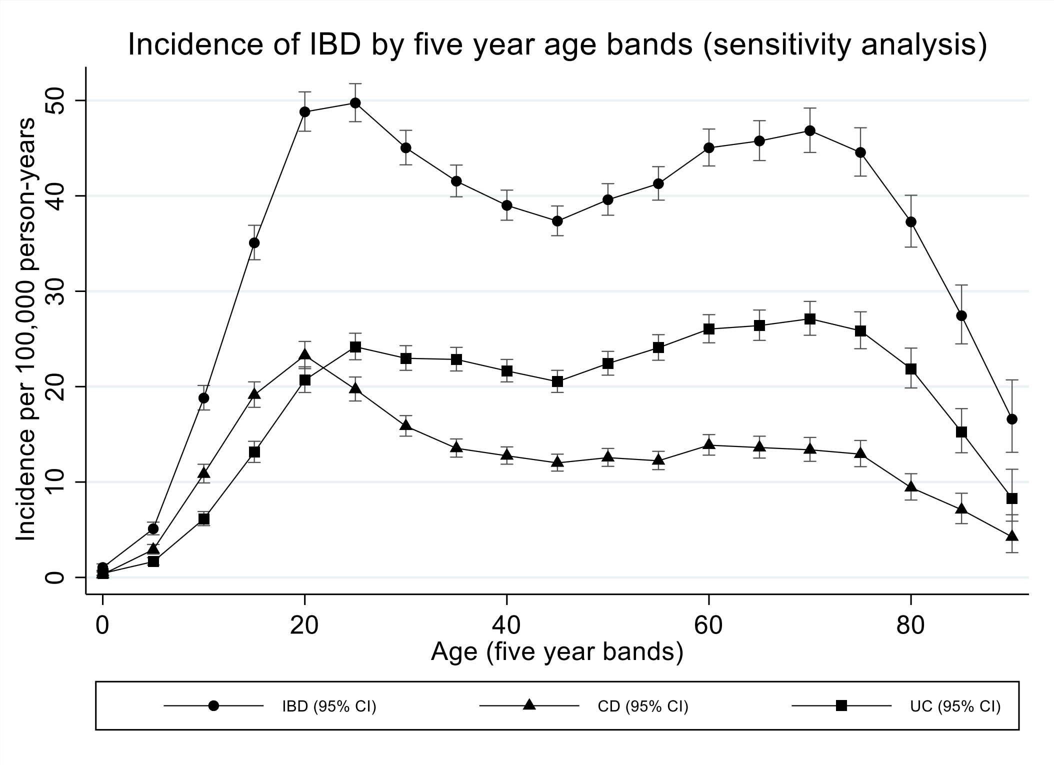


Read code lists:

*Any IBD*

| medcode | description |
|---|---|
| 14C4.11 | H/O: ulcerative colitis |
| 8Cc5.00 | Management of inflammatory bowel disease |
| 8Cc5.11 | Management of IBD (inflammatory bowel disease) |
| J08z900 | Orofacial Crohn's disease |
| J4...12 | Inflammatory bowel disease |
| J40..00 | Regional enteritis - Crohn's disease |
| J40..11 | Crohn's disease |
| J400.00 | Regional enteritis of the small bowel |
| J400000 | Regional enteritis of the duodenum |
| J400100 | Regional enteritis of the jejunum |
| J400200 | Crohn's disease of the terminal ileum |
| J400300 | Crohn's disease of the ileum unspecified |
| J400400 | Crohn's disease of the ileum NOS |
| J400500 | Exacerbation of Crohn's disease of small intestine |

For peer review only

| | |
|---|---|
| J400z00 | Crohn's disease of the small bowel NOS |
| J401.00 | Regional enteritis of the large bowel |
| J401000 | Regional enteritis of the colon |
| J401100 | Regional enteritis of the rectum |
| J401200 | Exacerbation of Crohn's disease of large intestine |
| J401z00 | Crohn's disease of the large bowel NOS |
| J401z11 | Crohn's colitis |
| J402.00 | Regional ileocolitis |
| J40z.00 | Regional enteritis NOS |
| J40z.11 | Crohn's disease NOS |
| J41..12 | Ulcerative colitis and/or proctitis |
| J410.00 | Ulcerative proctocolitis |
| J410100 | Ulcerative colitis |
| J410200 | Ulcerative rectosigmoiditis |
| J410300 | Ulcerative proctitis |
| J410400 | Exacerbation of ulcerative colitis |
| J410z00 | Ulcerative proctocolitis NOS |
| J413.00 | Ulcerative pancolitis |
| J41y.00 | Other idiopathic proctocolitis |
| J41yz00 | Other idiopathic proctocolitis NOS |
| J41z.00 | Idiopathic proctocolitis NOS |
| J4z6.00 | Indeterminate colitis |
| Jyu4000 | [X]Other Crohn's disease |
| Jyu4100 | [X]Other ulcerative colitis |

*CD*

| medcode | description |
|---|---|
| J08z900 | Orofacial Crohn's disease |
| J40..00 | Regional enteritis - Crohn's disease |
| J40..11 | Crohn's disease |
| J400.00 | Regional enteritis of the small bowel |

For peer review only

| | |
|---|---|
| J400000 | Regional enteritis of the duodenum |
| J400100 | Regional enteritis of the jejunum |
| J400200 | Crohn's disease of the terminal ileum |
| J400300 | Crohn's disease of the ileum unspecified |
| J400400 | Crohn's disease of the ileum NOS |
| J400500 | Exacerbation of Crohn's disease of small intestine |
| J400z00 | Crohn's disease of the small bowel NOS |
| J401.00 | Regional enteritis of the large bowel |
| J401000 | Regional enteritis of the colon |
| J401100 | Regional enteritis of the rectum |
| J401200 | Exacerbation of Crohn's disease of large intestine |
| J401z00 | Crohn's disease of the large bowel NOS |
| J401z11 | Crohn's colitis |
| J402.00 | Regional ileocolitis |
| J40z.00 | Regional enteritis NOS |
| J40z.11 | Crohn's disease NOS |
| Jyu4000 | [X]Other Crohn's disease |

*UC*

| **medcode** | **description** |
|---|---|
| 14C4.11 | H/O: ulcerative colitis |
| J41..12 | Ulcerative colitis and/or proctitis |
| J410.00 | Ulcerative proctocolitis |
| J410100 | Ulcerative colitis |
| J410200 | Ulcerative rectosigmoiditis |
| J410300 | Ulcerative proctitis |
| J410400 | Exacerbation of ulcerative colitis |
| J410z00 | Ulcerative proctocolitis NOS |
| J413.00 | Ulcerative pancolitis |
| J41y.00 | Other idiopathic proctocolitis |
| J41yz00 | Other idiopathic proctocolitis NOS |

For peer review only

| | |
|---|---|
| J41z.00 | Idiopathic proctocolitis NOS |
| Jyu4100 | [X]Other ulcerative colitis |

*Drugs related to IBD*

| **drugcode** | **genericname** |
|---|---|
| 30104978 | Adalimumab 40mg/0.4ml solution for injection pre-filled syringes |
| 30105978 | Adalimumab 40mg/0.4ml solution for injection pre-filled syringes |
| 30106978 | Adalimumab 40mg/0.4ml solution for injection pre-filled disposable devices |
| 53189979 | Ciclosporin 50mg capsules |
| 53190979 | Ciclosporin 100mg capsules |
| 53191979 | Ciclosporin 25mg capsules |
| 53913979 | Mesalazine 800mg gastro-resistant tablets |
| 54552979 | Mesalazine 400mg gastro-resistant tablets |
| 55164978 | Mesalazine 4g modified-release granules sachets sugar free |
| 55165978 | Mesalazine 4g modified-release granules sachets sugar free |
| 55193978 | Mercaptopurine 75mg tablets |
| 55575979 | Ciclosporin 50mg capsules |
| 55576979 | Ciclosporin 100mg capsules |
| 55577979 | Ciclosporin 25mg capsules |
| 55799978 | Mercaptopurine 30mg capsules |
| 56901978 | Ciclosporin 100mg capsules |
| 56902978 | Ciclosporin 25mg capsules |
| 58121979 | Mercaptopurine 25mg tablets |
| 58800979 | Mesalazine 1.2g gastro-resistant modified-release tablets |
| 60097979 | Prednisolone 20mg/application foam enema |
| 60124979 | Ciclosporin 50mg capsules |
| 60584979 | Mesalazine 3g gastro-resistant modified-release granules sachets sugar free |
| 60585979 | Mesalazine 3g gastro-resistant modified-release granules sachets sugar free |
| 61544979 | Adalimumab 40mg/0.8ml solution for injection vials |
| 61545979 | Adalimumab 40mg/0.8ml solution for injection vials |
| 62953979 | Sulfasalazine 250mg/5ml oral suspension sugar free |

For peer review only

| | |
|---|---|
| 64115979 | Methotrexate 25mg/3ml solution for injection pre-filled syringes |
| 64868979 | Mesalazine 2g modified-release granules sachets sugar free |
| 65098979 | Sulfasalazine 500mg/5ml oral suspension |
| 67089979 | Mesalazine 1.2g gastro-resistant modified-release tablets |
| 69316979 | Azathioprine 125mg/5ml oral suspension |
| 70274978 | Azathioprine 50mg tablets |
| 70879979 | Adalimumab 40mg prefilled pen |
| 70880979 | Adalimumab 40mg/0.8ml solution for injection pre-filled disposable devices |
| 72688978 | Ciclosporin 50mg capsules |
| 73065978 | Methotrexate 7.5mg/0.15ml solution for injection pre-filled disposable devices |
| 73066978 | Methotrexate 7.5mg/0.15ml solution for injection pre-filled disposable devices |
| 73067978 | Methotrexate 30mg/0.6ml solution for injection pre-filled disposable devices |
| 73068978 | Methotrexate 30mg/0.6ml solution for injection pre-filled disposable devices |
| 73069978 | Methotrexate 27.5mg/0.55ml solution for injection pre-filled disposable devices |
| 73071978 | Methotrexate 25mg/0.5ml solution for injection pre-filled disposable devices |
| 73072978 | Methotrexate 25mg/0.5ml solution for injection pre-filled disposable devices |
| 73073978 | Methotrexate 22.5mg/0.45ml solution for injection pre-filled disposable devices |
| 73074978 | Methotrexate 22.5mg/0.45ml solution for injection pre-filled disposable devices |
| 73075978 | Methotrexate 20mg/0.4ml solution for injection pre-filled disposable devices |
| 73076978 | Methotrexate 20mg/0.4ml solution for injection pre-filled disposable devices |
| 73077978 | Methotrexate 17.5mg/0.35ml solution for injection pre-filled disposable devices |
| 73078978 | Methotrexate 17.5mg/0.35ml solution for injection pre-filled disposable devices |
| 73079978 | Methotrexate 15mg/0.3ml solution for injection pre-filled disposable devices |
| 73080978 | Methotrexate 15mg/0.3ml solution for injection pre-filled disposable devices |
| 73081978 | Methotrexate 12.5mg/0.25ml solution for injection pre-filled disposable devices |
| 73082978 | Methotrexate 12.5mg/0.25ml solution for injection pre-filled disposable devices |
| 73083978 | Methotrexate 10mg/0.2ml solution for injection pre-filled disposable devices |
| 73084978 | Methotrexate 10mg/0.2ml solution for injection pre-filled disposable devices |
| 76424978 | Mesalazine 1g modified-release tablets |
| 76878978 | Methotrexate 2.5mg tablets |
| 78442979 | Methotrexate 50mg/2ml solution for injection vials |

For peer review only

| | |
|---|---|
| 78447979 | Methotrexate 25mg/1ml solution for injection pre-filled syringes |
| 78449979 | Methotrexate 20mg/0.8ml solution for injection pre-filled syringes |
| 78452979 | Methotrexate 12.5mg/0.5ml solution for injection pre-filled syringes |
| 79522979 | Mercaptopurine 25mg/5ml oral suspension |
| 79524979 | Mercaptopurine 20mg/ml oral suspension |
| 79739978 | Sulfasalazine 500mg gastro-resistant tablets |
| 79867978 | Mesalazine 400mg gastro-resistant tablets |
| 80925979 | Azathioprine 25mg/5ml oral suspension |
| 80927979 | Azathioprine 25mg/5ml oral solution |
| 80928998 | Mesalazine 3g gastro-resistant modified-release granules sachets sugar free |
| 80929979 | Azathioprine 20mg/5ml oral suspension |
| 80929998 | Mesalazine 3g gastro-resistant modified-release granules sachets sugar free |
| 80961979 | Azathioprine 10mg/5ml oral suspension |
| 81193998 | Mesalazine 1g modified-release tablets |
| 81194998 | Mesalazine 1g modified-release tablets |
| 81282998 | Ciclosporin 100mg capsules |
| 81283998 | Ciclosporin 50mg capsules |
| 81284998 | Ciclosporin 25mg capsules |
| 81490998 | Methotrexate 27.5mg/0.55ml prefilled syringes |
| 81491998 | Methotrexate 22.5mg/0.45ml prefilled syringes |
| 81492998 | Methotrexate 17.5mg/0.35ml prefilled syringes |
| 81493998 | Methotrexate 12.5mg/0.25ml prefilled syringes |
| 81494998 | Methotrexate 27.5mg/0.55ml solution for injection pre-filled syringes |
| 81495998 | Methotrexate 22.5mg/0.45ml solution for injection pre-filled syringes |
| 81496998 | Methotrexate 17.5mg/0.35ml solution for injection pre-filled syringes |
| 81498998 | Methotrexate 12.5mg/0.25ml solution for injection pre-filled syringes |
| 81638998 | Methotrexate 30mg/1.5ml solution for injection pre-filled syringes |
| 81640998 | Methotrexate 25mg/1.25ml solution for injection pre-filled syringes |
| 81642998 | Methotrexate 20mg/1ml solution for injection pre-filled syringes |
| 81643998 | Methotrexate 7.5mg/0.75ml prefilled syringes |
| 81683998 | Mesalazine 1g suppositories |

For peer review only

| | |
|---|---|
| 81689998 | Mesalazine 500mg gastro-resistant tablets |
| 81690998 | Mesalazine 500mg gastro-resistant tablets |
| 81771998 | Ciclosporin 25mg capsules |
| 81772998 | Mesalazine 800mg gastro-resistant tablets |
| 81773998 | Ciclosporin 100mg capsules |
| 81774998 | Ciclosporin 50mg capsules |
| 81815998 | Methotrexate 30mg/0.6ml prefilled syringes |
| 81816998 | Methotrexate 30mg/0.6ml solution for injection pre-filled syringes |
| 81868998 | Mesalazine 400mg gastro-resistant tablets |
| 82203998 | Azathioprine 50mg tablets |
| 82204998 | Azathioprine 25mg tablets |
| 82480998 | Ciclosporin 100mg capsules |
| 82481998 | Ciclosporin 50mg capsules |
| 82482998 | Ciclosporin 25mg capsules |
| 82840998 | Methotrexate 25mg/0.5ml prefilled syringes |
| 82841998 | Methotrexate 20mg/0.4ml prefilled syringes |
| 82842998 | Methotrexate 15mg/0.3ml prefilled syringes |
| 82843998 | Methotrexate 10mg/0.2ml prefilled syringes |
| 82844998 | Methotrexate 7.5mg/0.15ml prefilled syringes |
| 82845998 | Methotrexate 25mg/0.5ml solution for injection pre-filled syringes |
| 82846998 | Methotrexate 20mg/0.4ml solution for injection pre-filled syringes |
| 82847998 | Methotrexate 15mg/0.3ml solution for injection pre-filled syringes |
| 82848998 | Methotrexate 10mg/0.2ml solution for injection pre-filled syringes |
| 82849998 | Methotrexate 7.5mg/0.15ml solution for injection pre-filled syringes |
| 83228978 | Ciclosporin 50mg capsules |
| 83229978 | Ciclosporin 100mg capsules |
| 83485998 | Azathioprine 10mg capsules |
| 83503998 | Mesalazine 1.5g gastro-resistant modified-release granules sachets sugar free |
| 83504998 | Mesalazine 1.5g gastro-resistant modified-release granules sachets sugar free |
| 83559978 | Mercaptopurine 50mg tablets |
| 83743998 | Mesalazine 2g modified-release granules sachets sugar free |

For peer review only

| | |
|---|---|
| 83769998 | Sulfasalazine 250mg/5ml oral suspension sugar free |
| 83987998 | Mesalazine 2g modified-release granules sachets sugar free |
| 84059998 | Mesalazine 1g modified-release granules sachets sugar free |
| 84209998 | Mesalazine 800mg gastro-resistant tablets |
| 84290998 | Mesalazine 1.2g gastro-resistant modified-release tablets |
| 84291998 | Mesalazine 1.2g gastro-resistant modified-release tablets |
| 84438998 | Methotrexate 2mg/ml oral solution sugar free |
| 84439998 | Methotrexate 10mg/5ml oral suspension |
| 84636998 | Budesonide 2mg foam enema |
| 84637998 | Budesonide 2mg foam enema |
| 84741998 | Prednisolone 40mg/100ml enema |
| 84920998 | Ciclosporin 250mg/5ml solution for infusion ampoules |
| 84921998 | Ciclosporin 50mg/1ml solution for infusion ampoules |
| 84927998 | Ciclosporin 250mg/5ml solution for infusion ampoules |
| 84928998 | Ciclosporin 50mg/1ml solution for infusion ampoules |
| 85097998 | Methotrexate 1g/40ml solution for injection vials |
| 85100998 | Methotrexate 7.5mg/5ml oral suspension |
| 85188978 | Methotrexate 2.5mg tablets |
| 85252998 | Azathioprine 50mg/5ml oral suspension |
| 85556998 | Mercaptopurine oral solution |
| 85560998 | Mesalazine 800mg gastro-resistant tablets |
| 85639998 | Methotrexate 7.5mg/0.75ml prefilled syringes |
| 85640998 | Methotrexate 10mg/ml prefilled syringes |
| 85641998 | Methotrexate 15mg/1.5ml prefilled syringes |
| 85642998 | Methotrexate 20mg/2ml prefilled syringes |
| 85643998 | Methotrexate 25mg/2.5ml prefilled syringes |
| 85644998 | Methotrexate 7.5mg/0.75ml solution for injection pre-filled syringes |
| 85645998 | Methotrexate 10mg/1ml solution for injection pre-filled syringes |
| 85646998 | Methotrexate 15mg/1.5ml solution for injection pre-filled syringes |
| 85648998 | Methotrexate 20mg/2ml solution for injection pre-filled syringes |
| 85650998 | Methotrexate 25mg/2.5ml solution for injection pre-filled syringes |

For peer review only

| | |
|---|---|
| 85737998 | Methotrexate 5g/50ml solution for infusion vials |
| 85738998 | Methotrexate 1g/10ml solution for injection vials |
| 85776998 | Methotrexate oral solution |
| 85777998 | Methotrexate 12.5mg/5ml oral suspension |
| 86327998 | Methotrexate 2.5mg/5ml oral suspension |
| 86339998 | Methotrexate 5mg/0.2ml solution for injection pre-filled syringes |
| 86342998 | Methotrexate 22.5mg/0.9ml solution for injection pre-filled syringes |
| 86343998 | Methotrexate 15mg/0.6ml solution for injection pre-filled syringes |
| 86344998 | Methotrexate 12.5mg/0.5ml solution for injection pre-filled syringes |
| 86345998 | Methotrexate 10mg/0.4ml solution for injection pre-filled syringes |
| 86427998 | Methotrexate 17.5mg/0.7ml solution for injection pre-filled syringes |
| 86434998 | Methotrexate 20mg/0.8ml solution for injection pre-filled syringes |
| 86435998 | Methotrexate 25mg/1ml solution for injection pre-filled syringes |
| 86436998 | Methotrexate 500mg/20ml solution for injection vials |
| 86437998 | Methotrexate 50mg/2ml solution for injection vials |
| 86438998 | Methotrexate 7.5mg/0.3ml solution for injection pre-filled syringes |
| 86439998 | Methotrexate 30mg/1.2ml solution for injection pre-filled syringes |
| 86440998 | Methotrexate 27.5mg/1.1ml solution for injection pre-filled syringes |
| 86470998 | Azathioprine 250mg/5ml oral solution |
| 86519998 | Azathioprine 50mg/5ml oral solution |
| 87675998 | Mercaptopurine 10mg capsules |
| 87757998 | Mercaptopurine 10mg tablets |
| 87761998 | Mesalazine 400mg gastro-resistant tablets |
| 87859998 | Adalimumab 40mg prefilled syringes |
| 87862998 | Adalimumab 40mg injection |
| 87909998 | Mesalazine 1g gastro-resistant modified-release granules sachets sugar free |
| 87910998 | Mesalazine 500mg gastro-resistant modified-release granules sachets sugar free |
| 87911998 | Mesalazine 500mg gastro-resistant modified-release granules sachets sugar free |
| 88489998 | Balsalazide 750mg capsules |
| 88492998 | Balsalazide 750mg capsules |
| 88517998 | Mesalazine 400mg gastro-resistant tablets |

For peer review only

| | |
|---|---|
| 88519998 | Sulfasalazine 500mg gastro-resistant tablets |
| 89238998 | Budesonide 2mg/100ml enema |
| 89239998 | Budesonide 2mg/100ml enema |
| 89244979 | Adalimumab 40mg prefilled syringes |
| 89245979 | Adalimumab 40mg/0.8ml solution for injection pre-filled syringes |
| 89460998 | Hydrocortisone 1% / Pramocaine 1% foam enema |
| 89598997 | Sulfasalazine 3g/100ml retention enema |
| 89598998 | Sulfasalazine 500mg suppositories |
| 89604997 | Sulfasalazine 3g/100ml retention enema |
| 89604998 | Sulfasalazine 500mg suppositories |
| 89610997 | Sulfasalazine 250mg/5ml oral suspension |
| 89610998 | Sulfasalazine 500mg tablets |
| 89616997 | Sulfasalazine 250mg/5ml oral suspension sugar free |
| 89616998 | Sulfasalazine 500mg gastro-resistant tablets |
| 89651998 | Infliximab 100mg powder for solution for infusion vials |
| 89992997 | Mesalazine 2g/59ml enema |
| 89992998 | Mesalazine 500mg suppositories |
| 90310979 | Prednisolone 20mg/application foam enema |
| 91215998 | Azathioprine 50mg tablets |
| 91309998 | Azathioprine capsules |
| 91373997 | Ciclosporin 10mg capsules |
| 91373998 | Ciclosporin 100mg/ml oral solution sugar free |
| 91601979 | Sulfasalazine 500mg gastro-resistant tablets |
| 92244990 | Ciclosporin 100mg capsules |
| 92245990 | Ciclosporin 50mg capsules |
| 92246990 | Ciclosporin 25mg capsules |
| 92346998 | Mesalazine 1g/application foam enema |
| 92347998 | Mesalazine 400mg gastro-resistant tablets |
| 92400998 | Olsalazine 500mg tablets |
| 92401998 | Olsalazine 250mg capsules |
| 92488997 | Methotrexate 2.5mg tablets |

For peer review only

| | |
|---|---|
| 92488998 | Methotrexate 5g/200ml solution for infusion vials |
| 92511998 | Infliximab 100mg powder for solution for infusion vials |
| 92544979 | Ciclosporin 50mg capsules |
| 92552979 | Ciclosporin 100mg capsules |
| 92555979 | Ciclosporin 100mg capsules |
| 92558979 | Ciclosporin 25mg capsules |
| 92559979 | Ciclosporin 25mg capsules |
| 92566979 | Azathioprine 25mg tablets |
| 92570979 | Azathioprine 25mg tablets |
| 92571979 | Azathioprine 25mg tablets |
| 92579979 | Azathioprine 50mg tablets |
| 92639979 | Methotrexate 5g/200ml solution for infusion vials |
| 92650979 | Methotrexate 200mg/8ml solution for injection vials |
| 92655979 | Methotrexate 50mg/2ml solution for injection vials |
| 92764997 | Mesalazine 1g modified-release granules sachets sugar free |
| 92764998 | Mesalazine 500mg modified-release tablets |
| 92930998 | Azathioprine 50mg tablets |
| 92989996 | Ciclosporin 100mg capsules |
| 92989997 | Ciclosporin 50mg capsules |
| 92989998 | Ciclosporin 25mg capsules |
| 93074990 | Methotrexate 2.5mg tablets |
| 93623996 | Mesalazine 250mg suppositories |
| 93623997 | Mesalazine 1g/application foam enema |
| 93623998 | Mesalazine 1g/100ml enema |
| 93624996 | Mesalazine 1g suppositories |
| 93624997 | Mesalazine 250mg modified release tablets |
| 93624998 | Mesalazine 1g/100ml enema |
| 93728992 | Mesalazine 500mg modified-release tablets |
| 94042990 | Sulfasalazine 500mg gastro-resistant tablets |
| 94078992 | Azathioprine 100 mg tab |
| 94153998 | Hydrocortisone 10% foam aerosol enema |

For peer review only

| | |
|---|---|
| 94155997 | Hydrocortisone retention enema |
| 94155998 | Hydrocortisone 10% foam aerosol enema |
| 94308990 | Azathioprine 50mg tablets |
| 94417992 | Azathioprine 50 mg sus |
| 94437997 | Olsalazine 500mg tablets |
| 94437998 | Olsalazine 250mg capsules |
| 94438997 | Olsalazine 500mg tablets |
| 94438998 | Olsalazine 250mg capsules |
| 94451998 | Prednisolone 20mg/application foam enema |
| 94452998 | Prednisolone 20mg/application foam enema |
| 94564992 | Mesalazine 500mg modified-release tablets |
| 94593997 | Ciclosporin 10mg capsules |
| 94593998 | Ciclosporin 50mg capsules |
| 94600990 | Methotrexate 20mg/0.8ml solution for injection pre-filled syringes |
| 94690992 | Azathioprine 125 mg tab |
| 94691992 | Azathioprine 10 mg tab |
| 94697998 | Azathioprine 50mg tablets |
| 94818998 | Sulfasalazine 250mg/5ml oral suspension |
| 95041990 | Mesalazine 400mg gastro-resistant tablets |
| 95153992 | Azathioprine 10mg tablets |
| 95252990 | Azathioprine 50mg tablets |
| 95255998 | Sulfasalazine 500mg gastro-resistant tablets |
| 95256996 | Sulfasalazine 3g/100ml enema |
| 95256997 | Sulfasalazine 500mg suppositories |
| 95256998 | Sulfasalazine 500mg gastro-resistant tablets |
| 95589998 | Sulfasalazine 250mg/5ml oral suspension |
| 95725990 | Azathioprine 50mg tablets |
| 95866998 | Methotrexate 25mg/ml injection |
| 95867996 | Methotrexate 5mg/2ml solution for injection vials |
| 95867997 | Methotrexate 10mg tablets |
| 95867998 | Methotrexate 2.5mg tablets |

For peer review only

| | |
|---|---|
| 95868996 | Methotrexate 50mg/3ml Injection |
| 95868997 | Methotrexate 5g/50ml solution for infusion vials |
| 95868998 | Methotrexate 200mg/8ml solution for injection vials |
| 95869997 | Methotrexate 10mg tablets |
| 95869998 | Methotrexate 2.5mg tablets |
| 95888997 | Mesalazine 250mg gastro-resistant tablets |
| 95888998 | Mesalazine 400mg gastro-resistant tablets |
| 95890998 | Mercaptopurine 50mg tablets |
| 95891998 | Mercaptopurine 50mg tablets |
| 96177998 | Hydrocortisone 1% / pramocaine 1% foam enema |
| 96199990 | Azathioprine 50mg tablets |
| 96279990 | Methotrexate 2.5mg tablets |
| 96580998 | Ciclosporin 50mg/1ml solution for infusion ampoules |
| 96581996 | Ciclosporin 100mg capsules |
| 96581997 | Ciclosporin 25mg capsules |
| 96581998 | Ciclosporin 100mg/ml oral solution sugar free |
| 96608996 | Mesalazine 2g/59ml enema |
| 96608997 | Mesalazine 1g suppositories |
| 96608998 | Mesalazine 500mg suppositories |
| 96659996 | Mesalazine 1g/application foam enema |
| 96659997 | Mesalazine 500mg suppositories |
| 96659998 | Mesalazine 250mg suppositories |
| 96752989 | Methotrexate 10mg tablets |
| 96752990 | Methotrexate 2.5mg tablets |
| 96803990 | Sulfasalazine 500mg gastro-resistant tablets |
| 96820988 | Methotrexate 5mg/2ml solution for injection vials |
| 96883990 | Mesalazine 400mg gastro-resistant tablets |
| 96916992 | Mesalazine 500mg modified-release tablets |
| 96922989 | Azathioprine 50mg tablets |
| 96922990 | Azathioprine 25mg tablets |
| 96932998 | Azathioprine 50mg powder for solution for injection vials |

For peer review only

| | |
|---|---|
| 96933998 | Azathioprine 50mg tablets |
| 96934997 | Azathioprine 50mg powder for solution for injection vials |
| 96934998 | Azathioprine 25mg tablets |
| 97036997 | Azathioprine 10mg tablets |
| 97036998 | Azathioprine 50mg tablets |
| 97280998 | Sulfasalazine 500mg gastro-resistant tablets |
| 97281996 | Sulfasalazine 3g/100ml retention enema |
| 97281997 | Sulfasalazine 500mg suppositories |
| 97281998 | Sulfasalazine 500mg tablets |
| 97287998 | Hydrocortisone acetate & pramocaine foam |
| 97362990 | Sulfasalazine 500mg gastro-resistant tablets |
| 97363990 | Sulfasalazine 500mg gastro-resistant tablets |
| 97381998 | Mesalazine 400mg gastro-resistant tablets |
| 97719989 | Sulfasalazine 500mg gastro-resistant tablets |
| 97719990 | Sulfasalazine 500mg gastro-resistant tablets |
| 97764998 | Mesalazine 250mg gastro-resistant tablets |
| 97785990 | Azathioprine 50mg tablets |
| 98001992 | Mesalazine 250mg gastro-resistant tablets |
| 98013988 | Methotrexate 5g/50ml solution for infusion vials |
| 98211990 | Azathioprine 50mg tablets |
| 98238996 | Ciclosporin 25mg capsules |
| 98238998 | Ciclosporin 100mg/ml oral solution sugar free |
| 98365990 | Sulfasalazine 500mg gastro-resistant tablets |
| 98639990 | Azathioprine 50mg tablets |
| 98640990 | Azathioprine 50mg tablets |
| 98950997 | Ciclosporin 50mg capsules |
| 98950998 | Ciclosporin 100mg capsules |
| 98958988 | Methotrexate 2.5mg tablets |
| 98959989 | Methotrexate 10mg tablets |
| 98959990 | Methotrexate 2.5mg tablets |
| 99374990 | Sulfasalazine 500mg gastro-resistant tablets |

For peer review only

| | |
|---|---|
| 99394979 | Hydrocortisone 10% foam aerosol enema |
| 99472979 | Sulfasalazine 500mg gastro-resistant tablets |
| 99486979 | Mesalazine 400mg gastro-resistant tablets |
| 99487979 | Mesalazine 400mg gastro-resistant tablets |
| 99488979 | Mesalazine 400mg gastro-resistant tablets |
| 99490979 | Mesalazine 1g modified-release granules sachets sugar free |
| 99492979 | Mesalazine 1g suppositories |
| 99494979 | Mesalazine 1g suppositories |
| 99495979 | Mesalazine 500mg modified-release tablets |
| 99498979 | Mesalazine 500mg modified-release tablets |
| 99583996 | Mesalazine 1g modified-release granules sachets sugar free |
| 99583997 | Mesalazine 500mg modified-release tablets |
| 99583998 | Mesalazine 250mg modified-release tablet |
| 99797989 | Azathioprine 25mg tablets |
| 99797990 | Azathioprine 50mg tablets |
| 99798990 | Azathioprine 50mg tablets |
| 99799990 | Azathioprine 50mg tablets |
| 99956998 | Methotrexate 5mg/2ml solution for injection vials |

*IBD symptoms*

| **medcode** | **description** |
|---|---|
| 1625.00 | Abnormal weight loss |
| 1625.11 | Abnormal weight loss - symptom |
| 1627.00 | Unintentional weight loss |
| 196..11 | Abdominal pain type |
| 1962.00 | Colicky abdominal pain |
| 1963.00 | Non-colicky abdominal pain |
| 1969.00 | Abdominal pain |
| 196B.00 | Painful rectal bleeding |
| 196C.00 | Painless rectal bleeding |
| 197..13 | Site of abdominal pain |

For peer review only

| | |
|---|---|
| 1971.00 | Central abdominal pain |
| 197A.00 | Generalised abdominal pain |
| 197A.11 | General abdominal pain-symptom |
| 197B.00 | Upper abdominal pain |
| 197C.00 | Lower abdominal pain |
| 19E6.00 | Blood in faeces |
| 19E6.11 | Blood in faeces symptom |
| 19EH.00 | Mucus in faeces |
| 19F..00 | Diarrhoea symptoms |
| 19F..11 | Diarrhoea |
| 19F..12 | Loose stools |
| 19F2.00 | Diarrhoea |
| 19F3.00 | Spurious (overflow) diarrhoea |
| 19F4.00 | Toddlers diarrhoea |
| 19FZ.00 | Diarrhoea symptom NOS |
| 19FZ.11 | Diarrhoea & vomiting, symptom |
| 19G..00 | Diarrhoea and vomiting |
| 1D1A.00 | Complaining of weight loss |
| 22A8.00 | Weight loss from baseline weight |
| 4737.11 | Melaena - O/E of faeces |
| 4762.00 | Faeces: fresh blood present |
| 4762.11 | Blood in faeces |
| 4763.00 | Faeces: mucous present |
| 4764.11 | Pus in faeces |
| A076.11 | Viral diarrhoea |
| A082.00 | Infectious diarrhoea |
| A082.11 | Travellers' diarrhoea |
| A082000 | Dysenteric diarrhoea |
| A082100 | Epidemic diarrhoea |
| A082z00 | Infectious diarrhoea NOS |
| A083.00 | Diarrhoea of presumed infectious origin |

For peer review only

| | |
|---|---|
| A083.11 | Diarrhoea & vomiting -? infect |
| Ayu0H00 | [X]Diarrhoea+gastroenteritis of presumed infectious origin |
| E264300 | Psychogenic diarrhoea |
| E264311 | Spurious diarrhoea |
| Eu45317 | [X]Psychogenic diarrhoea |
| J4...13 | Noninfective diarrhoea |
| J432.11 | Allergic diarrhoea |
| J433.11 | Dietetic diarrhoea |
| J43z.11 | Chronic diarrhoea |
| J4z..11 | Presumed noninfectious diarrhoea |
| J4zz.11 | Diarrhoea - presumed non-infectious |
| J521000 | Irritable bowel syndrome with diarrhoea |
| J524000 | Diarrhoea after gastrointestinal tract surgery |
| J525.00 | Functional diarrhoea |
| J573011 | Rectal bleeding |
| J573012 | PRB - Rectal bleeding |
| J681.11 | Blood in stool |
| J681.12 | Altered blood in stools |
| J681.13 | Blood in stools altered |
| L16y500 | Abdominal pain in pregnancy |
| Q46y100 | Neonatal diarrhoea |
| R077100 | [D] Stools loose |
| R090.00 | [D]Abdominal pain |
| R090400 | [D]Abdominal cramps |
| R090E00 | [D]Recurrent acute abdominal pain |
| R090H00 | [D]Upper abdominal pain |
| R090N00 | [D]Nonspecific abdominal pain |
| R090P00 | [D]Functional abdominal pain syndrome |
| R090y00 | [D]Other specified abdominal pain |
| R090z00 | [D]Abdominal pain NOS |
| R121200 | [D]Mucus in stool |

For peer review only

| | |
|---|---|
| R121300 | [D]Pus in stool |
| Ryu1100 | [X]Other and unspecified abdominal pain |
| 1623.00 | Weight decreasing |
| 197..11 | Flank pain |
| 197..12 | Iliac fossa pain |
| 197..14 | Subcostal pain |
| 1972.00 | Epigastric pain |
| 1973.00 | Left subcostal pain |
| 1974.00 | Right subcostal pain |
| 1975.00 | Left flank pain |
| 1976.00 | Right flank pain |
| 1977.00 | Right iliac fossa pain |
| 1978.00 | Left iliac fossa pain |
| 1979.00 | Suprapubic pain |
| 197D.00 | Right upper quadrant pain |
| 19E4.12 | C/O - melaena |
| R090000 | [D]Abdominal tenderness |
| R090500 | [D]Epigastric pain |
| R090600 | [D]Umbilical pain |
| R090700 | [D]Hypochondrial pain |
| R090800 | [D]Suprapubic pain |
| R090900 | [D]Pain in right iliac fossa |
| R090A00 | [D]Pain in left iliac fossa |
| R090J00 | [D]Right upper quadrant pain |
| R090K00 | [D]Left upper quadrant pain |
| R090L00 | [D]Left lower quadrant pain |
| R090M00 | [D]Right lower quadrant pain |

For peer review only

STROBE Statement—Checklist of items that should be included in reports of ***cohort studies***

| | Item No | Recommendation | Page No |
|---|---|---|---|
| **Title and abstract** | 1 | (*a*) Indicate the study's design with a commonly used term in the title or the abstract<br>(*b*) Provide in the abstract an informative and balanced summary of what was done and what was found | 1,5 |
| **Introduction** | | | |
| Background/rationale | 2 | Explain the scientific background and rationale for the investigation being reported | 6 |
| Objectives | 3 | State specific objectives, including any prespecified hypotheses | 6 |
| **Methods** | | | |
| Study design | 4 | Present key elements of study design early in the paper | 7 |
| Setting | 5 | Describe the setting, locations, and relevant dates, including periods of recruitment, exposure, follow-up, and data collection | 7 |
| Participants | 6 | (*a*) Give the eligibility criteria, and the sources and methods of selection of participants. Describe methods of follow-up<br>(*b*) For matched studies, give matching criteria and number of exposed and unexposed | 7 |
| Variables | 7 | Clearly define all outcomes, exposures, predictors, potential confounders, and effect modifiers. Give diagnostic criteria, if applicable | 7,8 |
| Data sources/ measurement | 8* | For each variable of interest, give sources of data and details of methods of assessment (measurement). Describe comparability of assessment methods if there is more than one group | 7 |
| Bias | 9 | Describe any efforts to address potential sources of bias | 8 |
| Study size | 10 | Explain how the study size was arrived at | 7 |
| Quantitative variables | 11 | Explain how quantitative variables were handled in the analyses. If applicable, describe which groupings were chosen and why | 8 |
| Statistical methods | 12 | (*a*) Describe all statistical methods, including those used to control for confounding<br>(*b*) Describe any methods used to examine subgroups and interactions<br>(*c*) Explain how missing data were addressed<br>(*d*) If applicable, explain how loss to follow-up was addressed<br>(*e*) Describe any sensitivity analyses | 8 |
| **Results** | | | |
| Participants | 13* | (a) Report numbers of individuals at each stage of study—eg numbers potentially eligible, examined for eligibility, confirmed eligible, included in the study, completing follow-up, and analysed<br>(b) Give reasons for non-participation at each stage<br>(c) Consider use of a flow diagram | 9 |
| Descriptive data | 14* | (a) Give characteristics of study participants (eg demographic, clinical, social) and information on exposures and potential confounders<br>(b) Indicate number of participants with missing data for each variable of interest<br>(c) Summarise follow-up time (eg, average and total amount) | 9 |
| Outcome data | 15* | Report numbers of outcome events or summary measures over time | 9,10,11 |


For peer review only - http://bmjopen.bmj.com/site/about/guidelines.xhtml

| | | | |
|---|---|---|---|
| Main results | 16 | (*a*) Give unadjusted estimates and, if applicable, confounder-adjusted estimates and their precision (eg, 95% confidence interval). Make clear which confounders were adjusted for and why they were included<br>(*b*) Report category boundaries when continuous variables were categorized<br>(*c*) If relevant, consider translating estimates of relative risk into absolute risk for a meaningful time period | 9,10,11 |
| Other analyses | 17 | Report other analyses done—eg analyses of subgroups and interactions, and sensitivity analyses | 11 |
| **Discussion** | | | |
| Key results | 18 | Summarise key results with reference to study objectives | 12 |
| Limitations | 19 | Discuss limitations of the study, taking into account sources of potential bias or imprecision. Discuss both direction and magnitude of any potential bias | 12 |
| Interpretation | 20 | Give a cautious overall interpretation of results considering objectives, limitations, multiplicity of analyses, results from similar studies, and other relevant evidence | 12,13 |
| Generalisability | 21 | Discuss the generalisability (external validity) of the study results | 12 |
| **Other information** | | | |
| Funding | 22 | Give the source of funding and the role of the funders for the present study and, if applicable, for the original study on which the present article is based | 14 |

*Give information separately for exposed and unexposed groups.

**Note:** An Explanation and Elaboration article discusses each checklist item and gives methodological background and published examples of transparent reporting. The STROBE checklist is best used in conjunction with this article (freely available on the Web sites of PLoS Medicine at http://www.plosmedicine.org/, Annals of Internal Medicine at http://www.annals.org/, and Epidemiology at http://www.epidem.com/). Information on the STROBE Initiative is available at http://www.strobe-statement.org.

For peer review only



For peer review only - http://bmjopen.bmj.com/site/about/guidelines.xhtml
